# A new goniopholidid crocodyliform, *Hulkepholis rori* sp. nov. from the Camarillas Formation (early Barremian) in Galve, Spain)

**DOI:** 10.7717/peerj.7911

**Published:** 2019-10-31

**Authors:** Ignacio Arribas, Angela D. Buscalioni, Rafael Royo Torres, Eduardo Espílez, Luis Mampel, Luis Alcalá

**Affiliations:** 1 Departamento de Biología, Paleontología, Universidad Autónoma de Madrid, Cantoblanco, Madrid, Spain; 2 Museo Aragonés de Paleontología, Fundación Conjunto Paleontológico de Teruel-Dinopolis, Teruel, Aragón, Spain

**Keywords:** Crocodyliform, Goniopholididae, Systematics, Palatogenesis, Postrostral module, Cretaceous, Maestrazgo Basin

## Abstract

**Background:**

The neosuchian crocodyliform genus *Hulkepholis* constitutes the longirostral lineage of the European Goniopholididae. It comprises two species ranging from the Valanginian of southern England to the lower Albian of the northern Teruel (Spain). A new species of *Hulkepholis* is described based on a partially complete skull from the lower Barremian Camarillas Formation. We investigate its phylogenetic position and the palatal patterns among members of Goniopholididae and the closely related Thalattosuchia and Tethysuchia.

**Methods:**

Phylogenetic relationships were investigated with two matrices using a previously published dataset as the basis: the first differed only by the addition of the new species, the second had newly discovered states for 11 characters, the new species plus several additional specimens of *Hulkepholis* and *Anteophthalmosuchus*. Both matrices were processed using TNT v. 1.1, in a heuristic analysis of maximum parsimony, with tree bisection and reconnection 1,000 random addition replicates and saving the 10 most parsimonious trees per replicate, and up to 10 suboptimal trees to calculate Bremer supports. The skull geometry of nine species from Thalattosuchia, Tethysuchia and Goniopholididae was explored to test shape variation between the rostral and postrostral modules, and to visualize the differences on the secondary palate. A set of 18 landmarks was used to delimit significant anatomical features, and the skulls were isotropically scaled using Adobe Illustrator, with the longest skull (*Sarcosuchus imperator*) as the baseline for comparison.

**Results:**

The European lineages of goniopholidids are two clades (*Nannosuchus* + *Goniopholis*) plus (*Hulkepholis* + *Anteophthalmosuchus*). The new species, *Hulkepholis rori* sp. nov, shares with the latter clade the following apormorphies: a long anterolateral postorbital process, postorbital process almost reaching the anterior jugal ramus, and basioccipital tubera with lateral edges turned posteriorly. *Anteophthalmosuchus* was found to be monophyletic, and *Hulkepholis* paraphyletic due to the poor preservation of *H. willetti*. *Hulkepholis rori* is distinguished by having vascular fossae and a mid-protuberance on the ventral surface of the basioccipital, and wide internal fossae in the quadrate. Among Goniopholididae differences on the secondary palate are the presence of a palatal cleft, the narrowness of the secondary choana, and a wide foramen of the median pharyngeal tube.

**Conclusions:**

The new species is the earliest *Hulkepholis* from the Iberian Peninsula. New characters have been recognized in the organization of the palate and in the occipital region raising unexpected questions on the evolution of Goniopholididae. The set of palatal characters is discussed as part of a singular palatogenesis in Goniopholididae. The protruding occipital areas suggest that the longirostral *Hulkepholis* would have had an aquatic lifestyle with particular neck and skull movements.

## Introduction

Goniopholididae is a well-known extinct family of neosuchian crocodyliforms. They have a Jurassic origin, with *Calsoyasuchus valliceps* (Tykoski et al., 2002) from North America, probably one of its earliest known members (Sinemurian-Pliensbachian) (Wilberg, Turner & Brochu, 2019). Some authors consider that the habitat of goniopholidids is analogous to that of lacustrine and estuarine modern Crocodylians because of their skull shape (i.e., platyrostry, with heterodonty, unique dorsal narial aperture, and jaw festooning (Buffetaut, 1982; Averianov, 2000; Schwarz, 2002; Tykoski et al., 2002; Salisbury & Naish, 2011). However, the family also possesses a set of primitive features (e.g., palatines participating in the choana, amphicoelous vertebrae, two rows of paravertebral osteoderms) together with several characteristic traits, such as an extremely flattened rostrum, maxillary depressions, two parasagittal palatal fossae, and an open cranioquadrate passage (Steel, 1973; Buffetaut, 1982; De Andrade et al., 2011; Adams, 2013). Despite their abundant and diverse fossil record, the phenotypic variability and functionality of goniopholidid features are not yet fully understood, which is corroborated by the incongruence between taxonomy and phylogeny (De Andrade et al., 2011; Allen, 2012; Pritchard et al., 2013; Adams, 2013; Puértolas-Pascual, Canudo & Sender, 2015; Martin, Delfino & Smith, 2016; Ristevski et al., 2018). Recent studies providing better and more comprehensive anatomical descriptions are correcting former misconceptions, and providing evidence of their extraordinary adaptations and diversity during the Mesozoic (De Andrade & Hornung, 2011; De Andrade et al., 2011; Salisbury & Naish, 2011; Pritchard et al., 2013; Ristevski et al., 2018).

**Figure 1:**
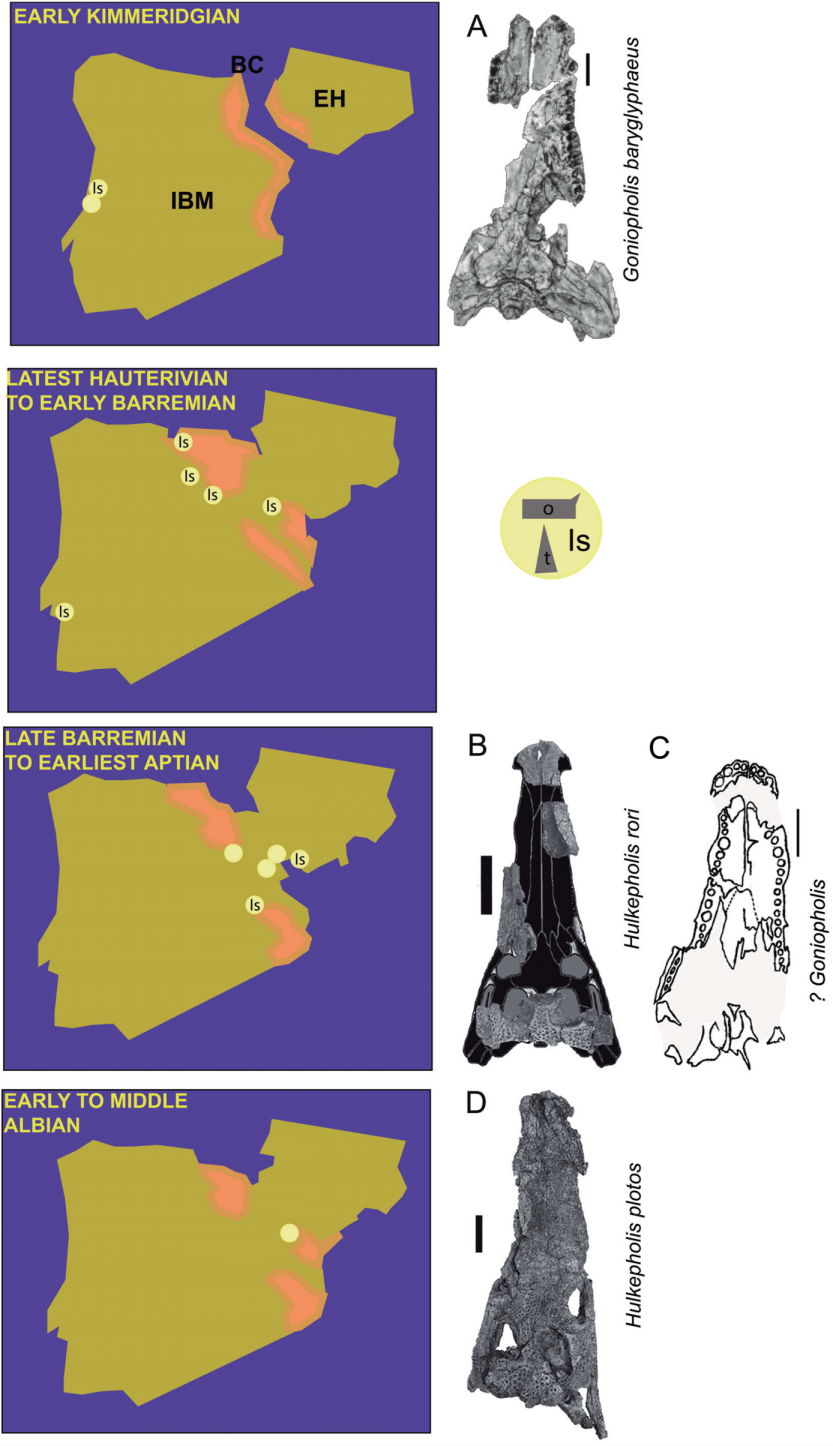
Paleogeographic maps of the Iberia Peninsula and Goniopholididae fossil record. Paleogeographic maps of the Iberia Peninsula showing the evolution of the Iberian rift system from the Kimmeridgian to mid Albian (based on the maps by Martín-Chivelet (2002)). The localities where goniopholidids have been reported are mostly located along different domains of the Iberian Basin in Spain. Kimmeridgian: Lusitania Basin, Alcobaça and Lourinhã Formations in Portugal; Hauterivian-Barremian: Iberian Basin, (A) Maestrazgo sub-basin, El Castellar, Camarillas and Artoles Formations, (B) Cameros sub-basin, Urbión, Golmayo, Castrillo de la Reina and Pinilla de los Moros Formations; Basque-Cantabrian Basin, Vega del Pas Formation, and Lusitania Basin, Papo-Seco Formation in Portugal; late Barremian to earliest Aptian: Iberian Basin, (A) south Iberian sub-basin, La Huérguina Formation; (B) Maestrazgo basin, Arcillas de Morella and Forcall Formations; and Albian: Iberian Basin, (A) Maestrazgo sub-basin, Escucha Formation. Data source: Brinkmann (1989), Buscalioni (1986a, 1986b), Buscalioni & Sanz (1987), Buscalioni et al. (2008, 2013), Canudo et al. (2008), Cuenca-Bescós et al. (1999), Fuentes-Vidarte et al. (2003), Figueiredo, Rosinal & Figuti (2015), Ortega et al. (1996), Puértolas-Pascual, Canudo & Sender (2015), Ruiz-Omeñaca & Canudo (2001), Ruiz-Omeñaca et al. (2004), Sánchez-Hernández, Benton & Naish (2007), Sastre García (2007), Schwarz (2002). Most representative fossils per time: (A) *Goniopholis baryglyphaeus* from Alcobaça Formation; scale = 1 cm. Figure modified from Schwarz (2002). (B) *Hulkepholis rori* from Camarillas Formation; scale bar = 5 cm. Skull outline modified from figures 3E and 7C of De Andrade et al. (2011) and De Andrade & Hornung (2011), respectively. (C) ?*Goniopholis* from Urbion D Formation; scale bar = 5 cm. Figure modified from Ortega et al. (1996). (D) *Hulkepholis plotos*, Albian, Escucha Formation; scale bar = 5 cm. Photograph source credit: Luis Alcalá. Abbreviations: IBM, Iberian Meseta; EH, Ebro high; BC, Basque-Cantabrian Basin; Is, isolated material (o, osteoderms; t, teeth). Areas in pink mark coastal and continental environments (Martín-Chivelet, 2002).

The four European goniopholidid genera, ranging from the Kimmeridgian to the Albian, include *Goniopholis* (Owen, 1841), *Anteophthalmosuchus* (Salisbury & Naish, 2011), *Hulkepholis* (Buscalioni et al., 2013) and the monospecific genus *Nannosuchus gracilidens* (Owen, 1879). Their detailed descriptions by De Andrade et al. (2011) have become invaluable to understanding the evolution of European goniopholidids. The Iberian goniopholidid fossil record, which ranges from the Kimmeridgian to the early Albian (Fig. 1), is generally composed of fragmentary and non-diagnostic elements (teeth and osteoderms; Buscalioni, 1986b; Buscalioni et al., 2013), but there are some specimens complete enough to allow diagnosis: *Goniopholis baryglyphaeus* (Schwarz, 2002) from Guimarota (Portugal), and *Hulkepholis plotos* and *Anteophthalmosuchus escuchae* (Buscalioni et al., 2013) from Ariño (Spain). Other specimens partially preserved were assigned to *Goniopholis* sp. (Ortega et al., 1996), *Goniopholis* cf. *simus* (Buscalioni, 1986a, 1986b), *Goniopholis* cf. *crassidens* (Buscalioni, 1986b; Buscalioni & Sanz, 1987), Goniopholididae indet. (Buscalioni et al., 2013), and *Anteophthalmosuchus* cf. *escuchae* (Puértolas-Pascual, Canudo & Sender, 2015).

This contribution describes a new species, *H. rori* sp. nov., based on a partial skull from Galve (Teruel, Spain) discovered at the locality of Cabezo Santa Bárbara 2 (Camarillas Formation, lower Barremian; Díaz-Molina & Yébenes, 1987; Soria de Miguel, 1997), and preliminarily reported by Buscalioni & Sanz (1987). The Galve specimen enriches the anatomical information on the goniopholidid basicranium, palate and quadrate. We also explore the early evolution of the Iberian species belonging to the genera *Anteophthalmosuchus* and *Hulkepholis*. The Galve specimen is compared with the goniopholidids from Ariño (Escucha Formation, lower Albian), including recently prepared specimens from this locality, with new relevant anatomical information. We present a phylogenetic analysis based on characters defined and described in Ristevski et al. (2018) in order to confirm the species composition of Goniopholididae (De Andrade et al., 2011; Pritchard et al., 2013; Adams, 2013; Ristevski et al., 2018). The study aims to verify the phylogenetic position of the newly described goniopholidid from Galve, and to specify the characteristics of the two erected *Hulkepholis* and *Anteophthalmosuchus* species from Ariño (*H. plotos* and *Anteophthalmosuchus escuchae*) (Buscalioni et al., 2013). The palatal patterns among members of the family Goniopholididae are also discussed in comparison to species of Thalattosuchia and Tethysuchia.

## Materials and Methods

### Specimens, characters and coding

The specimen of the new species was found at Cabezo Santa Bárbara (CB2) in Galve, province of Teruel (Spain). It comprises an incomplete skull, isolated osteoderms and teeth. The specimen is temporarily deposited in the CBP collection of the Universidad Autónoma de Madrid (UAM) and it will be permanently housed at the AR. Since all the fragments were found articulated and there is correlation in size and morphology among them, we can establish that they belong to a single individual (Fig. 1B).

**Figure 2:**
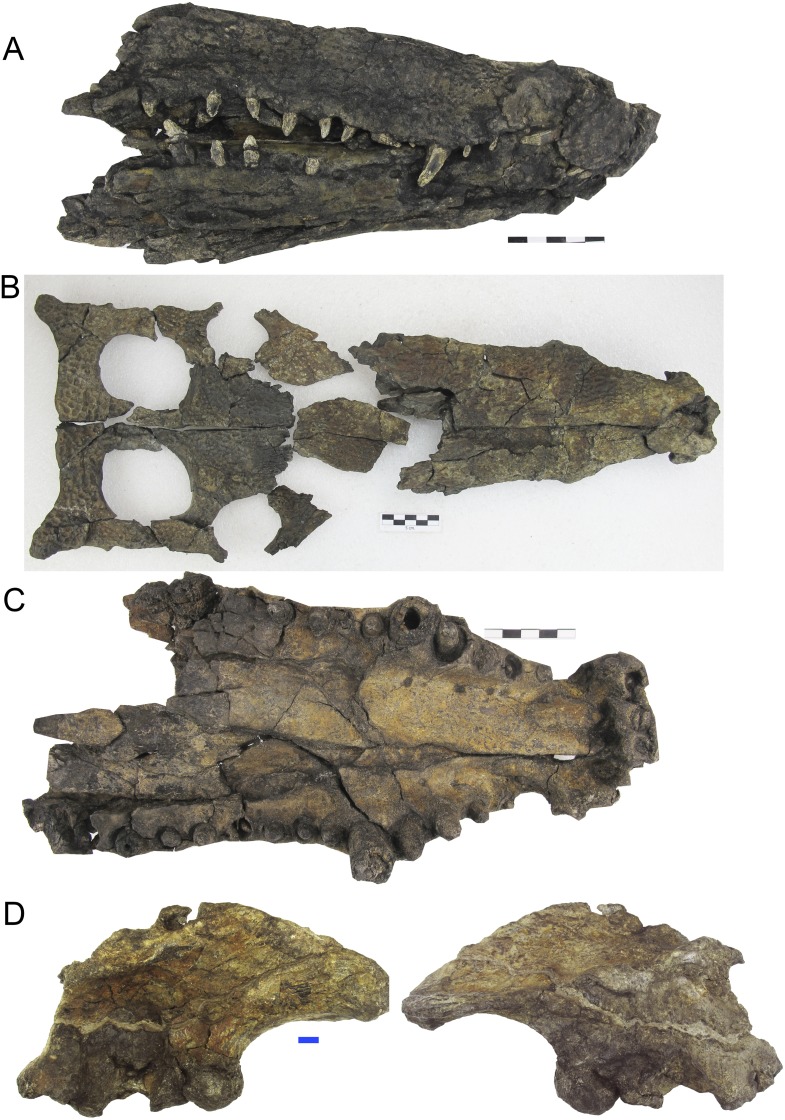
Skulls of *Hulkepholis plotos* from Ariño (Teruel, Spain). New specimens attributed to *Hulkepholis plotos* from Ariño (Escucha Formation). (A) AR-1-1625 snout and mandible. (B) AR-1-5762, dorsal skull bones. (C) Palatal view of the specimen AR-1-5762, showing the disposition of the large maxillary teeth (the sixth is smaller than the third, and closely set to the fifth). Scale bars five cm. See also Fig. 3 for comparison with *Anteophthalmosuchus escuchae*. (D) Right ilium (AR-1-5652) associated to cranial material of *Hulkepholis plotos* in lateral and medial views. Scale bar one cm. Photographs Jorge Escudero.

The *Hulkepholis* and *Anteophthalmosuchus* specimens from the AR show differences in the volume of the bones due to local fossilization processes that lead to preservation in lignite, especially when they contain pyrite (Newman, 1998; D’Anastassio, Capasso & Pallozzi, 2014). However, the locality has yielded exceptionally abundant monotaxic concentrations of crocodyliform bones (87 identified to date). The following individuals of *H. plotos* of different ontogenetic ages (Fig. 2) were compared: AR-1-2045, the holotype; AR-1-5762, dorsal skull bones (excluding neurocranium and mandibles) and isolated postcranial elements; and AR-1-1625, snout and mandibles with isolated postcranial bones. *Anteophthalmosuchus escuchae* is represented by the holotype AR-1-1097 and by the small specimen (AR-1-3422) formerly named “Ariño Goniopholididae indet.” in Buscalioni et al. (2013). Herein, we revise their corresponding morphologies, the coding of their phylogenetic characters, and correct some aspects of their earlier description by Buscalioni et al. (2013).

**Figure 3:**
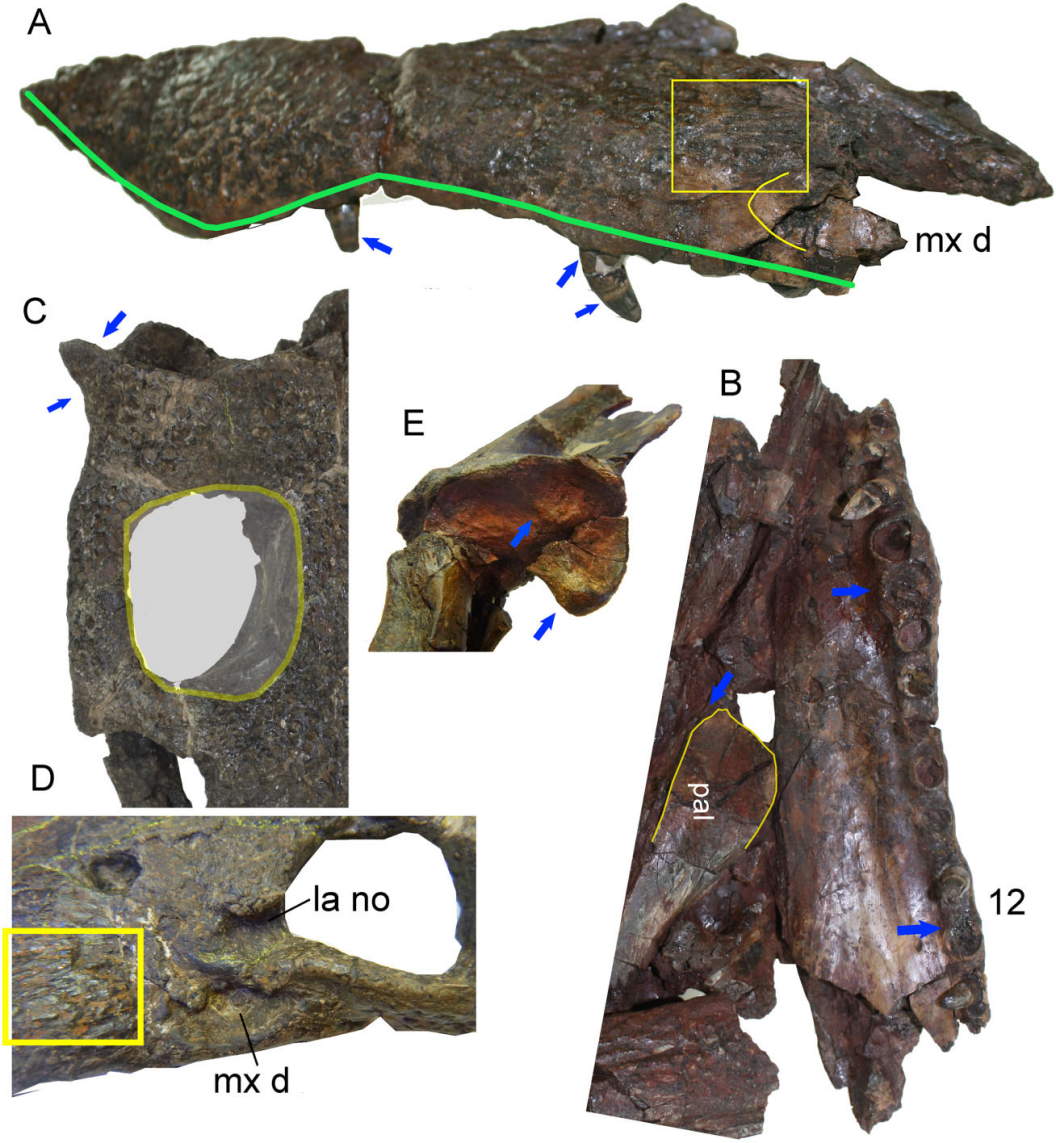
Skull details of *Anteophthalmosuchus escuchae*. (A) Lateral view of the holotype (AR-1-1097) showing the maxillary contour (note a second posterior festooning); the box encloses the radiating ornamentation in front of the maxillary depressions; the arrows point the extruded root and the enamel rings. (B) Palatal view of the holotype showing the distribution of the enlarged maxillary teeth; the palate anterior process has a mid-notch at the sagittal suture. (C) Skull table of the subadult specimen (AR-1-3422) showing the pointed and smooth squamosal prong, and the subrectangular fossa with straight lateral and medial margins. (D) Detail of the orbit of the subadult specimen, showing the radiating ornamentation (in box) and the lacrimal notch at the orbit. (E) Glenoid area of the holotype showing the oblique mid crest and the anteromedial protuberance. Abbreviations: la no, lacrimal notch; mx d, maxillary depressions; pal, palatine. Photographs Jorge Escudero.

The study also revises the descriptions of other goniopholidids: *Amphicotylus lucasii*, *Amphicotylus stovalli* (Mook, 1942; Allen, 2012) and *Eutretauranosuchus delfsi* (Mook, 1967; Smith et al., 2010; Pritchard et al., 2013), to test character definitions and coding for the phylogenetic analysis. We followed Montefeltro, Andrade & Larsson (2016), Young & Bierman (2019), and Dufeau & Witmer (2015) for anatomical terms related to external and middle ear sinuses.

### Phylogenetic methods

The phylogenetic analysis is based on characters defined and described in Ristevski et al. (2018), which center on confirmed species belonging to Goniopholididae, but exclude the terminal taxa PIN 4174-1 and *Kansajsuchus extensus* (Ristevski et al., 2018). The first phylogenetic analysis was based on the dataset of Ristevski et al. (2018) to which only the species *H. rori* sp. nov. was added (Dataset S1). The second analysis, also based on Ristevski’s dataset, includes new states for the characters: 66, 101, 111, 139, 141, 151, 155, 221, 233, 247 and 288 (Dataset S2), as described in the section List of Characters (Characters S1). Dataset S2 incorporates several specimens referable to the same species of *H. plotos* and *Anteophthalmosuchus escuchae* (Table 1). This procedure, based on the exemplary method (Prendini, 2001; Schuh & Browe, 2009), seeks parsimonious estimates of the clades considering interspecific variation and missing entries of the analyzed specimens. The inclusion of data from different studies permitted the deciphering of major discrepancies in the characters among operational taxonomic unit (OTUs) due to poor preservation, morphological divergences, and even to characters ambiguously described. Phylogenies were inferred using TNT v. 1.1 (Goloboff, Farris & Nixon, 2008). All characters were equally weighted except for 26 characters that were treated as ordered in Ristevski’s dataset. A heuristic analysis of maximum parsimony, with tree bisection and reconnection was conducted with 1,000 random addition replicates and saving the 10 most parsimonious trees per replicate. Nodal support was assessed by performing Bremer support for up to 10 suboptimal trees, and bootstrap analyses set to 1,000 random replicates.

**Table 1:** List of *Hulkepholis* and *Anteophthalmosuchus* OTUs used in the cladistics analysis.

OTU	Specimen signature	Source of coding
*Hulkepholis willetti*	BMNHB 001876 (Booth Museum of Natural History collections in Brighton, UK)	Ristevski et al. (2018) (the taxon name is misspelled as *Hulkepholis*) and Arribas et al. (present contribution)
*Hulkepholis plotos*	Holotype, monotaxic concentration AR-1/56 (Museo Aragonés de Paleontología, Fundación Dinópolis, Teruel, Spain)	Ristevski et al. (2018) and Arribas et al. (present contribution)
*Hulkepholis plotos*	Monotaxic concentration AR-1/2 and AR-1/104 (Museo Aragonés de Paleontología, Fundación Dinópolis, Teruel, Spain)	Arribas et al. (present contribution)
*Hulkepholis rori*	Holotype CPB830-CPB8311 (Unidad de Paleontología, Universidad Autónoma de Madrid, Spain)	Arribas et al. (present contribution)
*Anteophthalmosuchus epikrato*r	IRSNB R47 (Institut Royal des Sciences Naturelles Bruxelles, Belgium)	Ristevski et al. (2018) The specimen was attributed to *A. hooleyi* by Martin, Delfino & Smith (2016)
*Anteophthalmosuchus epikrator*	Holotype IWCMS 2001.446 and IWCMS 2005.127 (Isle of Wight County Museums Services; Dinosaur Isle Museum and visitor attraction, Sandown, UK)	Ristevski et al. (2018)
*Anteophthalmosuchus hooleyi*	Holotype NHMUK PV R 3876 (Vertebrate paleontology collection of the Natural History Museum London, UK)	De Andrade et al. (2011) and Ristevski et al. (2018).
*Anteophthalmosuchus escuchae*	Holotype, monotaxic concentration AR-1/37 (Museo Aragonés de Paleontología, Fundación Dinópolis, Teruel, Spain)	Arribas et al. (present contribution) and Ristevski et al. (2018)
*Anteophthalmosuchus escuchae* (subadult)	Monotaxic concentration AR-1/62 (Museo Aragonés de Paleontología, Fundación Dinópolis, Teruel, Spain)	Arribas et al. (present contribution)

### Nomenclature

The nomenclatural act describing the species *H. rori* sp. nov. is presented below. The electronic version of this article in Portable Document Format represents a published work according to the International Commission on Zoological Nomenclature (ICZN), and hence the new names contained in the electronic version are effectively published under the ICZN from the electronic edition alone. This published work and the nomenclatural acts it contains have been registered in ZooBank, the online registration system for the ICZN. The ZooBank Life Science Identifiers (LSIDs) can be resolved and the associated information viewed through any standard web browser by appending the LSID to the prefix http://zoobank.org/. The LSID for this publication is: urn:lsid:zoobank.org:pub:A8446014-A73D-4D47-88B7-2EADECC81F32. The online version of this work is archived and available from the following digital repositories: PeerJ, PubMed Central and CLOCKSS.

### Shape variation

The skull geometry of nine species from the clade Thalattosuchia, Tethysuchia and Goniopholididae (Ristevski et al., 2018) was explored to test shape variation and proportions between the rostral and postrostral modules, and to visualize the differences on the secondary palate of the selected taxa. The sample includes the thalattosuchian *Pelagosaurus typus* (based on the reconstruction of Nr 2744 by Pierce & Benton (2006)), the pholidosaurs *Sarcosuchus imperator* (based on the reconstruction of MNN 604 by Sereno, Larsson & Sidor, 2001), *Elosuchus cherifiensis* (based on the specimen MNHN SAM 129, by De Broin (2002)) and the following Goniopholididae: *Sunosuchus junggarensis* (reconstruction of IVPP V10606 by Wu, Brinkman & Russell (1996)), *Eutetrauranosuchus delfsi* (CMNH 8028 by Pritchard et al. (2013)), *Amphicotylus stovalli* (OMNH 2392 by Allen (2012)), *G. kiplingi* (DORCM 12154 by De Andrade et al. (2011)), *H. willetti* (BMNHB01876, by Salisbury & Naish (2011)), *Anteophthalmosuchus epikrator* (IWCMS 2001.446 by Ristevski et al. (2018)), and *Anteophthalmosuchus hooleyi* (NHMUK PV R 3876, by Ristevski et al. (2018)). A set of landmarks was used to delimit significant anatomical features of the rostral and postrostral modules (Piras et al., 2013).The landmarks in dorsal view are: (1) premaxillary tip; (2) lateralmost premaxillary edge; (3) maxillary edge at 5th tooth; (4) edge posterior to jugal bar; (5) lateralmost quadratojugal edge; (6) quadratojugal-quadrate suture; (7) quadrate condyle; (8) medial edge of the quadrate; (9) squamosal tip; (10) parietal margin; (11) skull table posterior to orbital edge. A partial clipping of the ventral aspect of the skull has been also depicted and includes the ventral fenestrae and openings. The selected landmarks for the ventral skull are: (12) maxilla-palatine suture at the palate; (13) maxillary orthogonal edge; (14) end of the maxillary dental series; (15) pterygoid lateral and posteriormost tip; (16) palatine-pterygoid suture; (17) palatine-pterygoid suture at the suborbital fenestra; (18) posterior tip at the basioccipital ventral edge.

All skulls were scaled by applying an isotropic scaling, which applies a linear transformation enlarging or shrinking the skull by a scale factor that is the same in all directions. The uniform scaling was graphically performed using Adobe Illustrator (ver. 14.0) using the longest skull (*Sarcosuchus imperator*) as the baseline for comparison (i.e., scaling by the length of a baseline; Lele & Cole, 1996). The total cranial length of each specimen was calculated in relation to the baseline. To test the shape variation and proportions of the two modules all the skull contours were adjusted to a line that crossed the orbital center. This criterion of comparison is based on biological evidence that the eye diameter enlarges slowly as body mass increases, and that eye growth is dependent on the central nervous system (Ngwenya et al., 2013). The eye is a conservative area suitable for unveiling divergences in the proportion between the longitudinal and lateral expansions of the skull among species.

## Results

### Anatomy of the Ariño goniopholidids

The new material prepared from the Ariño coal mine site (AR-1) increases our knowledge of the anatomy of *Anteophthalmosuchus* and *Hulkepholis* goniopholidids, including cranial and postcranial elements. Among the species described from Ariño, *H. plotos* is undoubtedly the most complete. This species is represented by individuals of different ontogenetic sizes, including adults and a hyperadult (with exaggerated adult features; i.e., exhibiting hypermorphosis; Fig. 2). We recognize as polymorphies, and code as such, conditions observed in the hyperadult, such as the presence of a well-defined postnarial fossa with wide pits and a deep sulcus, the greater size of the supratemporal fossa relative to the orbits, the relative increase of the rostral length, the orbital lateral displacement, and the greater ornamentation of the squamosal at the posterolateral lobes.

The holotype of *Anteophthalmosuchus escuchae* is fragmentary and due to its poor preservation, the orbital contour was misinterpreted in Buscalioni et al. (2013). The anterior margin of the supratemporal fossa, which shows an anterior smooth platform, was identified as the right orbital border. The contour of the right orbit is collapsed and is opaque in the holotype. By reconsidering its skull length, the snout of *Anteophthalmosuchus escuchae* is now viewed as moderate, as in AR-1-3422. After coding the phylogenetic characters, the individual AR-1-3422, previously identified as Goniopholididae indet. by Buscalioni et al. (2013), fits the characteristics of *Anteophthalmosuchus escuchae* (Fig. 3). The suggested differences (i.e., squamosal-postorbital extension of the lateral suture, the extension of the ventral quadratojugal suture, and the diameter of the occipital condyle; Buscalioni et al., 2013, p. 119) might be due to its different ontogenetic size—AR-1-3422 is considered here a subadult specimen of *Anteophthalmosuchus escuchae*.

The taxonomic dissimilarities between the Iberian *Hulkepholis* and *Anteophthalmosuchus* are now clearer. These differences include rostral relative length, maxillary shape and ornamentation, maxillary teeth disposition, orbital disposition, the shaping of the supratemporal fenestra, the squamosal lobes, the shape of the quadrate condyles, the mandibular glenoid fossa and the basioccipital tubera (Table 2; Fig. 3). Ristevski et al. (2018) have remarked on a unique combination of characters for *Anteophthalmosuchus* as a genus, questioning the taxonomic status of *Anteophthalmosuchus escuchae* due to its preservation. This unique combination includes the following features:

**Table 2:** Taxonomic dissimilarities of the Iberian Goniopholididae.

Features	*Anteophthalmosuchus escuchae*	*Hulkepholis plotos*	*Hulkepholis rori*
Rostral relative length	Brevi to mesorostral (55–65%)	Sub-longirostral (59–69%)	Sub-longirostral (59–69%)
Lateral surface of maxilla at the posterior part	Narrow and dorsally curved (Fig. 3A)	Wide and vertical (Fig. 2A)	Wide and vertical
Enlarged anterior maxillary teeth	5th ~ 4th and 6th = 3rd (Fig. 3B)	5th > 4th and 6th small (as large as 2nd) (Fig. 2C)	?5th; 6th > 2nd or 3rd
Size of the posterior maxillary alveoli	6th to 11th subequal; 12th and 13th enlarged; and rear teeth subequal but diminished (Fig. 3B)	6th to 11th subequal; 12th and 13th enlarged; and rear teeth subequal but diminished (Fig. 2C)	6th on, subequal
Maxillary teeth	Extruded roots, and enamel with rings (Fig. 3A)	Not extruded root (Fig. 2A)	Not extruded roots
Orbital orientation	Dorsolateral (Fig. 3D)	Mostly lateral	Not preserved
Anterior end of the maxillary depressions	At level to lacrimo-maxillary suture (Fig. 3D)	At level to lacrimo-maxillary suture	At level to lacrimo-maxillary suture
Ornamentation of maxilla in front of the maxillary depressions	With ridges radiating anteromedially (see Fig. 3D)	Smooth maxilla	Smooth maxilla
Shape of supratemporal fossa	Rectangular, with anteroposterior axis prevailing (Fig. 3C)	Square-shaped, with both axes subequal (Fig. 2B)	? Square-shaped
Shape of squamosal prongs	Unornamented, no sulcus, pointed end laterally projected (Fig. 3C)	Ornamented with pits, no sulcus, and roundly end (Fig. 2B)	Corrugated ornamentation, with anterior sulcus, and roundly end
Quadrate medial condyle	Equals to the lateral condyle and separated by an intercondylar groove	Bulging medial condyle	Bulging medial condyle
Exoccipital ventral process	Reaches the base of basioccipital	Does not reach the base of basioccipital	Does not reach the base of basioccipital
Basioccipital tubera	Tubera curved to mid plane, and weakly corrugated margins	Tubera curved to mid plane, and corrugated margins	Tubera curved to mid plane, and corrugated margins
Foramina, ventral to occipital condyle	Absent	Absent	Present
Palatine-pterygoid contact	Deep mid sulcus at palatines	Flat	Not preserved
Palatino-maxillary suture	With a mid-notch (Fig. 3B)	Round	Not preserved
Ornamentation of frontal	Smooth	With a longitudinal intumescence	With a longitudinal intumescence
Glenoid fossa of retroarticular	With an oblique mid crest (Fig. 3E)	With a parasagittal mid-crest	Not preserved
Mid crest at the ventral border of basioccipital	Absent	Absent	Present
Parietal hornlets	Absent	Absent	Present

**Note:**

*Anteophthalmosuchus* and *Hulkepholis* in Figs. 2 and 3. Rostral relative length, from the premaxilla tip to the anterior border of the orbits with respect to the length up to the parietal border.

1. The ornamentation of subcircular pits follows the same pattern in both genera. In *Hulkepholis* and *Anteophthalmosuchus* the pits decrease in diameter anteroposteriorly. However, Ristevski et al. (2018) noticed that the groove-like ornamentation is sparser in *Anteophthalmosuchus* species, suggesting that the set of radiating grooves described in *Anteophthalmosuchus escuchae* should have been due to ontogeny. We have verified the presence of these ridges in the subadult (AR-1-3422) and in the holotype and consider that the character is not related to age, but to a short and bulging area in front of the maxillary depressions, and that this trait is unique to the Iberian *Anteophthalmosuchus* (Figs. 3A and 3D).
2. The presence of maxillary fossae is confirmed for *Anteophthalmosuchus escuchae*; it is fully visible in the subadult (AR-1-3422) (Fig. 3D). A small sector of the anterior maxillary fossae, smooth and shallow, is preserved in the holotype. In *Hulkepholis* and *Anteophthaltomuschus* the maxillary depressions are posteriorly situated compared to *Goniopholis*: the anterior margin is placed at the same level as the lacrimo-maxillary suture, whereas the posterior edge reaches the antorbital depression of the lacrimal.
3. The posterior prefrontal extension is barely visible in the holotype of *Anteophthalmosuchus escuchae*. However, in AR-1-3422, the posterior prefrontal extension is wider and the posterior prefronto-frontal suture is oblique (transverse) at the skull table. The prefrontal of *H. plotos*, by contrast, has narrow and parallel sides and slightly curves posteriorly (Fig. 2).
4. The interorbital crest reaches its maximum development in the genus *Goniopholis* and in *Amphicotylus*, whereas in *Hulkepholis* and *Anteophthalmosuchus* this crest is substantially reduced or absent. The subadult (AR-1-3422) *Anteophthalmosuchus escuchae* confirms the lack of an interorbital crest (not visible in the holotype). The fully adult *Hulkepholis* has a shallow intumescence formed by a distinct configuration of the pitting at the middle of the frontal and between the orbits (Fig. 2). A similar pattern is observed in *H. rori* sp. nov., with a longitudinal intumescence at the medial line of the frontal. Character (#139) proposed by De Andrade et al. (2011) has two states, (0) absent and (1) well defined, and it refers only to the transversal crest. We suggest herein the addition of a new state (2) presence of an interorbital hump on the frontal.
5. The palpebral is fully integrated into the medial orbital edge in AR-1-3422; it has a squared shape as in other *Anteophthalmosuchus* and differs from that of *H. plotos* and *H. willetti* (not preserved in *H. rori* sp. nov.) because it has curved median and lateral contours (Fig. 2).
6. The orbits are poorly preserved in the holotype of *Anteophthalmosuchus escuchae*, but in AR-1-3422 the orbits show a dorsal component, differing clearly from that of *Hulkepholis*, whose orbit faces laterally, almost hidden in dorsal view.
7. In *Goniopholis* the postorbital barely projects anteriorly, whereas in *Anteophthalmosuchus* and *Hulkepholis* the postorbital bears a long anterolateral projection which protects the orbit laterally. The anterolateral process of the postorbital is damaged in AR-1-3422 but it is preserved in the holotype of *Anteophthalmosuchus escuchae*. Ristevski et al. (2018) suggest that the process would constitute a significant portion of the lateral orbital margin in *Anteophthalmosuchus*, but the same condition occurs in *Hulkepholis* (Fig. 2); although in *H. plotos* it is broken, and a fragment of a long process is preserved.
8. The supratemporal fossa is sub-rectangular with a long anteroposterior axis in *Anteophthalmosuchus* but subquadrangular in all *Hulkepholis* species (both axes subequal in length) (Figs. 2 and 3C).
9. In all *Anteophthalmosuchus* species, the shape and ornamentation of the squamosal prongs is unornamented, and not buttressed. In *Anteophthalmosuchus escuchae* the prongs are pointed and laterally projected (Fig. 3C). In *H. plotos* the lobate prongs are rounded and ornamented with pits (Fig. 2B), whereas in *H. rori* sp. nov. they show a corrugated ornamentation and an anterior sulcus.
10. The shape of the quadrate condyles differs between *Anteophthalmosuchus* and *Hulkepholis*. The medial condyle in *H. plotos* is voluminous and dorsoventrally expanded. The asymmetry between the lateral and medial condyles is manifest also at the glenoid fossa. The medial fossa bends ventrally in *H. plotos*; it has a longitudinal mid-crest that divides the glenoid fossa. In the holotype of *Anteophthalmosuchus escuchae* the medial part of the fossa faces dorsally (Fig. 3E) and the crest is stout, displaced obliquely, and directed inward (Buscalioni et al., 2013). One of the features shared by both genera is the development of an anteromedial border at the glenoid area that forms a stout knob for the insertion of the muscle pterygoideus dorsalis. This feature has not been described in *Anteophthalmosuchus escuchae* from CCB-1 (Coco Corta Barrabasa site in Andorra, Teruel; Puértolas-Pascual, Canudo & Sender, 2015), but it is visible in the mandible of the specimen IRSNB R-47, described as the anteriomedial edge of the projected glenoid in Martin, Delfino & Smith (2016, fig. 7).
11. The similar shape of the iliac blade (Fig. 2D) between *Anteophthalmosuchus* and *Hulkepholis* (*H. plotos*). Although the ilia are not preserved in the specimens of *Anteophthalmosuchus escuchae*, *Anteophthalmosuchus hooleyi* (Ristevski et al., 2018, fig. 29) shares with *H. plotos* (Fig. 2D) a contiguous anterior margin of supraacetabular crest with the anterior margin of the ilium, and the absence of constrictions on the dorsal and ventral margins of the terminal part of the postacetabular process. These features are different in *Anteophthalmosuchus epikrator* (Ristevski et al., 2018).

### Geological and paleontological setting of Galve

**Figure 4:**
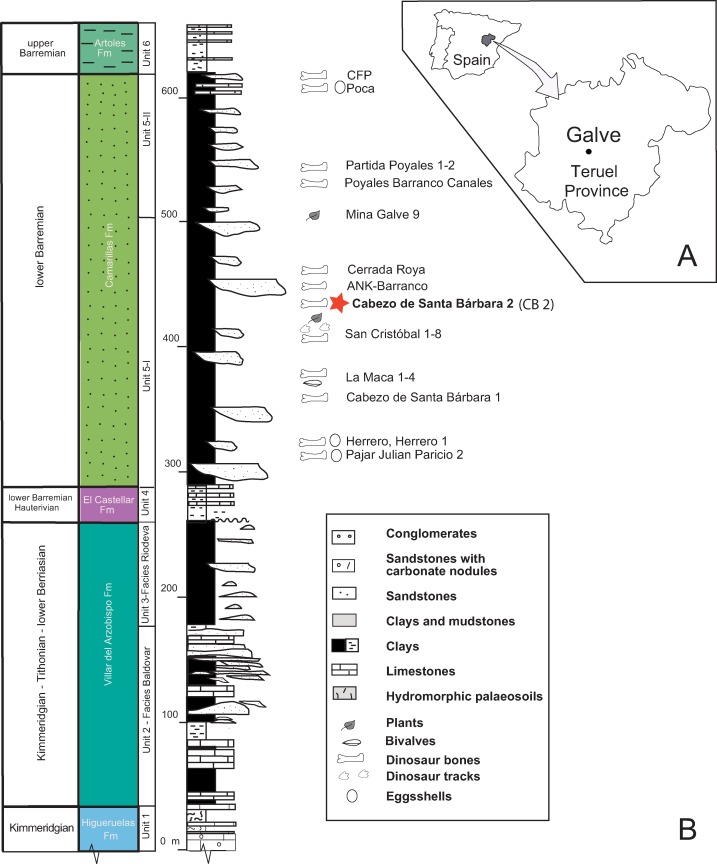
Location and geology of the studied area. (A) Location of Galve in the Teruel province. (B) The general stratigraphic section includes the Upper Jurassic—Lower Cretaceous Formations outcropping in Galve. The paleontological sites of the Camarillas Formation in Galve have been listed with the locations of Cabezo de Santa Bárbara (Coordinates: 40°39′46″N; 0°52′18″W); the star in color depicts Cabezo de Santa Bárbara 2 (CB2) where the described goniopholidid was discovered. The stratigraphic Units are based on Díaz-Molina & Yébenes (1987), and the ages of Higueruelas and Villar del Arzobispo Formations have been revised following data for these formations established in near sub-basins (Campos-Soto et al., 2017). Line drawing: Rafael RoyoTorres with full acknowledgment to the original publication titled “The anatomy, phylogenetic relationships, and stratigraphic position of the Tithonian–Berriasian Spanish sauropod dinosaur Aragosaurus ischiaticus,” p. 628, fig 2 in Zoological Journal of the Linnean Society, by the authors: (Royo-Torres R, Upchurch P, Mannion PD, Mas R, Cobos A, Gascó F, Alcalá L, and Sanz JL and edited by Maarten JM and Christenhusz FLS), year 2014; It is reproduced by permission of Oxford University Press (https://onlinelibrary.wiley.com/doi/10.1111/zoj.12144).

Galve is part of the Comarca Comunidad de Teruel and the Maestrazgo Cultural Park in the province of Teruel (Aragón). Galve lies in the Aragonian Branch of the Iberian Range and it is an important paleontological locality situated in an extraordinary geological area (Aurell et al., 2016; Campos-Soto et al., 2017) (Fig. 4). The paleontological fossil record ranges from the Kimmeridgian to the Barremian (Díaz-Molina & Yébenes, 1987) and the area contains several important vertebrate sites (Sanz et al., 1987; Ruiz-Omeñaca et al., 2004; Verdú et al., 2015). In the 1980s the Instituto de Paleontología Miquel Crusafont de Sabadell and UAM collaborated in the paleontological study of the area; more than 35 vertebrate taxa were determined (Buscalioni & Sanz, 1987), including the first new dinosaur described from Spain, *Aragosaurus ischiaticus* (Sanz et al., 1987; Royo-Torres et al., 2014).

The Cabezo Santa Bárbara 2 site is within the Camarillas Formation in the syncline of Galve (Galve sub-basin in of the Maestrazgo Basin). The Camarillas Formation was defined by Canérot et al. (1982) and Salas (1987) as in between the localities of Aguilar de Alfambra and Camarillas. This Formation reaches a thickness of 300 m at the area of Galve (Díaz-Molina & Yébenes, 1987). Its lower limit rests conformably over the lacustrine facies of the El Castellar Formation, while its top contacts the marine facies of the Artoles Formation (Salas et al., 1995; Soria de Miguel, 1997). The transition between the El Castellar and Camarillas Formations involves a rapid change of lithology, while it is gradual between the Camarillas and Artoles Formations.

The Camarillas Formation is composed of red or variously colored shaley silts and clays, sandstones (non-channelled sediments, overbank deposits), and mainly white sands and gravels (paleochannels), with occasional marl and limestone intercalations (Fig. 4). The depositional environment of the Camarillas Formation in the Galve sub-basin is the result of the activity of a fluvial system dominated at the base by low-sinuosity channels (Díaz-Molina & Yébenes, 1987). The ostracod assemblages composed of *Cypridea tuberculata*, *Timiriasevia* sp., *Paranotacythere galvensis, Fabanella boloniensis*, aff. *Macrodentina*, *Mediostricta*, and *M. gibbera* suggest an early Barremian age (Soria de Miguel, 1997). The charophyte assemblage belongs to the *Triquetra*-*Neimongolensis* biozone (sub-zone *Calcitrapus*; Schudack & Schudack, 2009; Martín-Closas, 1989), and the palynological assemblage of *Cicatricosisporites hughesi*, *Cicatricosisporites shallei* and *Plicatellapar viangulata* (Villanueva-Amadoz et al., 2015) supports an early Barremian age.

The vertebrate assemblage of the Camarillas Formation at Galve consists of sharks, bony fishes, amphibians, squamates, crocodyliforms, turtles, dinosaurs and mammals (Ruiz-Omeñaca et al., 2004, 2012; Badiola, Canudo & Cuenca-Bescós, 2011; Ruiz-Omeñaca, 2011; Pérez-García, Scheyer & Murelaga, 2013; Verdú et al., 2015). Archosaurs are diverse and apart from neosuchian crocodyliforms, include a new iguanodontian genus and species, “*Delapparentia turolensis”* (Ruiz-Omeñaca, 2011), which has been considered as an undetermined species of *Iguanodon* (Verdú et al., 2017), and the species *I. galvensis* (Verdú et al., 2015). Both dinosaurs were described using partially articulated skeletons and, in the case of *I. galvensis*, thirteen perinates were found together, suggesting they remained near their nests for some time, possibly congregated in nursery areas. In addition, ornithopod tracks, probably of an iguanodontian trackmaker, have also been found in the Barremian of Galve (Royo-Torres, Mampel & Alcalá, 2013). The first published reference to the crocodyliforms of Galve by Kühne (1966) described isolated teeth and unidentified osteoderms. Subsequently, Berg & Crusafont (1970) cited the finding of molariform-type teeth attributed to *Allognathosuchus*; later Buffetaut & Ford (1979) assigned them to *Bernissartia* sp. In 1984, more than 25 isolated crocodyliform teeth from La Cuesta de los Corrales site in Galve were described by Buscalioni & Sanz (1984). These authors updated the taxonomic list of mesoeucrocodylians for Galve comprising the families: Goniopholididae (*Goniopholis*), Atoposauridae (*Theriosuchus*) and Bernissartidae (*Bernissartia*) (Buscalioni & Sanz, 1987). Cabezo de Santa Bárbara is a classic outcrop of the Camarillas Formation of the Galve syncline (Díaz-Molina et al., 1984). The main site is Cabezo de Santa Bárbara 1 (CB1), but Ruiz-Omeñaca et al. (2004) referred to two sites, Santa Bárbara Norte and Cabezo de Santa Bárbara 2 (CB2) (Fig. 4). CB1 yielded fossils of *Iguanodon* sp. which were labeled CSBH (Sanz, Casanovas & Santafé, 1984). CB2 yielded the crocodilian fossil material studied here, which was labeled CBP and was attributed to cf. *Goniopholis* sp. (Buscalioni & Sanz, 1987). The fossil was previously prepared at the laboratory of Palaeontology, UAM and recently at the laboratory of the Foundation of Dinopolis in the Museo Aragonés de Paleontología.

## Systematic paleontology

Crocodyliformes Benton & Clark, 1988
Mesoeucrocodylia Whetstone & Whybrow, 1983
Neosuchia Benton & Clark, 1988
Goniopholididae Cope, 1875
*Hulkepholis* Buscalioni, Alcalá, Espílez & Mampel, 2013

**Type species**

*Hulkepholis* (=*Goniopholis*) *willetti* (Salisbury & Naish, 2011; fig. 24.2–24.4)

**Included species**

*Hulkepholis plotos* (Buscalioni et al., 2013), lower Albian, Ariño, Teruel, Spain, and *H. rori* sp. nov., lower Barremian, Galve, Teruel, Spain.

**Stratigraphic distribution**

From Valanginian of the Grinstead Clay Formation, Hastings Group, Wealden Supergroup to lower Albian of the Escucha Formation, Maestrazgo Basin, Eastern branch of the Iberian Range.

**Differential diagnosis (revised)**

*Hulkepholis* is distinguished from *Goniopholis* and *Anteophthalmosuchus* by the following combination of characters: sub-longirostral skull with a relative narrow rostrum with maximum width of maxillary at fifth teeth, 20–25% of maximum rostral length, and with maximum width of maxillary (at fifth maxillary teeth) 50–60% of skull width (between squamosal prongs); orbits facing laterally; fifth premaxillary tooth smallest, second or third largest; posterior part of maxilla facing laterally; incisive foramen slit or closed; two waves of enlarged teeth (3–4–5) at mid rostrum, plus a subtle wave at 12th tooth; maxilla with slight vertical festooning; ample inter-alveolar spaces; anterior border of palatines clearly surpassing anteriormost border of suborbital fenestrae; palatine anterior process as wide as long; interorbital shallow hump; squamosal lobe short and blunted, anteriorly delimited by a slight sulcus; palpebral robust and large, delta-like, postorbital palpebral absent (unknown in *H. rori* sp. nov., see below); exclusion of frontal at orbital contour; lateral processes of frontal arched laterodorsally, palpebral and postorbital curved dorsally; parietals or frontals occasionally unfused (shared with *Amphicotylus stovalli*, OMNH 2392); long rostral process on postorbital bar; supratemporal fossa much larger than orbit, and rounded supratemporal fenestrae (both axes subequal); frontoparietal suture straight and at anterior third of interfenestral bar; posterior nasals transversely widened (comprising more than 50% of rostrum); choana elongated (two times longer than wide) and mid-septate by a narrow vertical bony sheet of rectangular cross-section.

*Hulkepholis willetti* Salisbury & Naish, 2011

**Holotype**

BMNHB 001876, a nearly complete skull (Salisbury & Naish, 2011; fig. 24.2–24.4).

**Species diagnosis**

Lateral border of premaxilla aligned with maxillary contour at fifth tooth; second premaxillary alveolus larger than third; sixth alveolus equal in size to fourth; frontal anterior process wedging nasals, and prefrontals dividing nasals at posterior contact; prefrontal not participating to medial orbital margin; frontopostorbital suture at skull table curvilinear medially convex; choana midway between palatines and pterygoid, and choana posterior border forward of posterior edge of suborbital fenestra.

*Hulkepholis plotos* Buscalioni, Alcalá, Espílez & Mampel, 2013

**Holotype**

**Figure 5:**
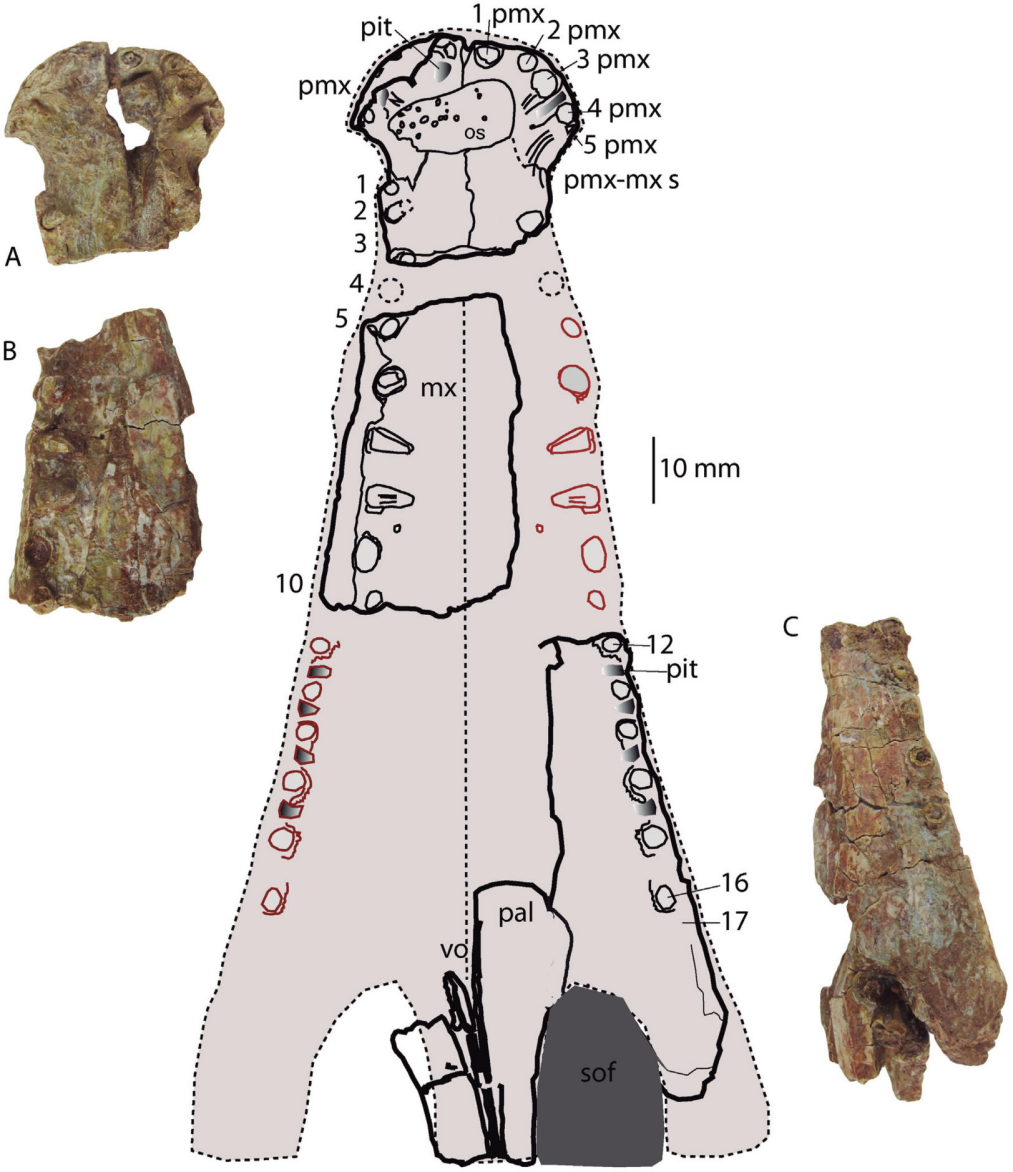
Reconstruction of the palatal region. *Hulkepholis rori* (Camarillas Formation of Galve, Teruel). (A) Fragment of premaxillae (CBP-839). (B) Anterior maxillary fragment (CBP-836). (C) Maxilla with part of the periorbital region (CBP-831). Abbreviations: mx, maxilla; os, osteoderm; pal, palatine; pmx, premaxilla; pmx-mx s, premaxillo-maxillary suture; sof, suborbital fenestra; vo, vomer; the teeth are numbered, and the pits derived from the occlusion of dentary tooth are in gray. Line drawing source credit: Angela D. Buscalioni; photograph souce credit: Ignacio Arribas.

AR-1/56, a partial skeleton comprising: AR-1-2045, an almost complete skull, three vertebrae (AR-1-2048, AR-1-4859-60), a rib (AR-1-2046), a metapodial (AR-1-2048), and three osteoderms (AR-1-2049, AR-1-4861-62) (Buscalioni et al., 2013; Figs. 4 and 5).

**Related material**

AR-1/2, a partial skeleton, and AR-1/104, a partial skeleton.

**Species diagnosis**

Premaxillary second alveolus larger than third; sixth maxillary alveolus set near fifth, and reduced in diameter (1/3 less than fifth); perinarial crest elevated; frontal anterior process triangular and acute with prefrontals dividing nasals at posterior contact; prefrontals barely participating in orbital margin; mid suture of frontals and/or parietals eventually unfused; squamosal lobe discrete with an anterior sulcus and ornamented with pits; frontopostorbital suture at skull table feebly curvilinear and medially concave; choana anterior edge posterior to posterior contour of palatal fenestra, and choanal aperture mostly within pterygoids; basioccipital (ventral to occipital condyle) long with lateral tubera extending slightly ventral to medial pharyngeal tube; postorbital rostral projection with an anterolateral wide lamina reaching mid orbit; exoccipital terminating dorsally to basioccipital tubera; bulged medial quadrate condyle; glenoid fossa of mandible divided by a crest and medial part bending ventrally; iliac blade with anterior margin of supraacetabular crest and anterior margin of ilium fused.

*Hulkepholis rori* sp. nov. Royo-Torres, Espílez, Mampel *&* Alcalá (Figs. 5–12)
urn:lsid:zoobank.org:act:E2EA1AFF-DDA3-42E3-912F-72A633FA8A0A; Arribas, Buscalioni

**Etymology**

The species name *rori* is the Latin word denominating “for the dew”

**Holotype**

Partially complete skull preserved in parts: premaxilla (CBP-839), anterior maxillary fragment (CBP-836), maxilla with part of periorbital region (CBP-831), skull table and occipital area (CBP-835); quadrate condyles (CBP-838); part of left hemimandible (CBP-832); and isolated bone fragments with two teeth (CBP-833), osteoderms (CBP-837), postorbital spine (CBP-830); pterygoid wing (CBP-8310), articular (glenoid fossa) fragment (CBP- 834); undetermined mandibular fragment (CBP-8311). The holotype is temporarily deposited in the collection of the UAM and it will be permanently housed at the AR.

**Type locality, horizon and age**

Cabezo Santa Bárbara 2, Galve, Teruel province, Spain. Material found in red to green-purple clays, located at the upper sedimentary succession of the unit 5-I (Díaz-Molina & Yébenes, 1987) of the Camarillas Formation, lower Barremian (Díaz-Molina & Yébenes, 1987; Villanueva-Amadoz et al., 2015).

**Species diagnosis**

Third premaxillary alveolus largest; sparse maxillary dental series with inter-alveolar spaces longer than alveolar diameter; posterior maxillary teeth set in raised alveoli; parietal with parasagittal hornlets; squamosal lobe with corrugated ornamentation; supraoccipital with a mid-knob; at base of occipital condyle two foramina set in a depression divided by a ridge; mid-crested protuberance backwardly directed at base of basioccipital; choana posterior edge at pterygoid.

### Description

The fossil is partially covered by a crust; in some areas the periosteum is cracked. It is slightly compressed on the right side, and the premaxillary and maxillary dental borders are rather crushed medially. The nasals are displaced and broken, leaving the nasopharyngeal cavity exposed. The maxilla is distorted, the palatine bar collapsed into the nasopharyngeal cavity, uplifting and displacing the frontal and the right prefrontal toward the left side of the skull. The mandible is dorsoventrally compressed, and the dentary is posteriorly displaced. Only the mid-part of the left mandibular ramus, the anterior edge of the internal mandibular fenestra, and a fragment of the posterior region of the dentary are preserved. The skull table, basicranium and occipital area are, otherwise, nicely preserved. The orbital outline is not traceable: a partially complete lacrimal, prefrontals and frontal comprise the periorbital region. The infratemporal area is compressed dorsoventrally. Quadrates are preserved, the left quadrate is articulated, and the right was detached from the skull for its description. Ventrally, the two vomers are exposed. The pterygoids are broken but a fragment of the pterygoid wings is preserved.

### General features

The skull represents a sub-longirostral medium-sized individual (Table 3). According to the reconstruction the estimated skull length is 240 mm. The anterior tip of the skull is round; the premaxilla-maxillary notch is subcircular in dorsal aspect. The contour of the lateral contact between the premaxilla and the maxilla is straight and does not present a strong convex profile. The surface of the skull table is flat. The ornamentation is composed by pits of one to three mm disposed on the skull table, quadratojugal, angular and surangular, whereas on the maxilla, dentary and the anteroventral surface of the angular the sculpture is made of pits and longitudinal ridges to smooth bone surfaces.

**Table 3:** Skull measurements (in mm) of *Hulkepholis rori*.

Skull table width at the posterior edge of the supratemporal fossa	85.7	Foramen magnum width	15.9
Intertemporal bar width at middle	11	Occipital condyle width	10.8
Supratemporal bar width	9.6	Width between quadrates	118.8
Supratemporal fossa width	29	Width between squamosal tips	89.6
Premaxillary width	35	Estimation of rostral length	ca.170
Premaxillary width at notch	26	Estimation of skull length	ca.240
Quadrate condyle width	19.4	Rostral width at 5th maxillary tooth	ca.43.2

**Figure 6:**
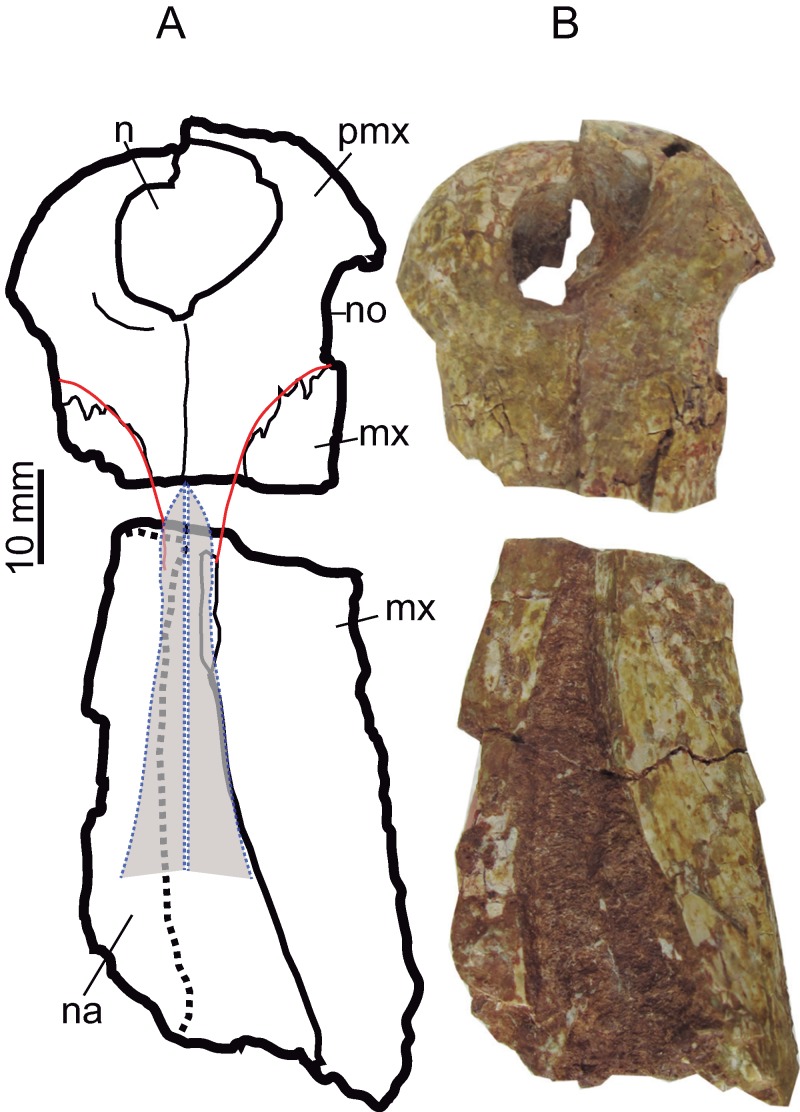
Rostral bones. Premaxillae (CBP-839) and anterior maxillary fragment (CBP-836) of *Hulkepholis rori* from Galve, dorsal view. (A) Line drawing, in which the nasals and the premaxillary-maxillary suture have been colored as interpreted. (B) Photographs. Abbreviations: mx, maxilla; n, naris; na, nasal; no, notch premaxilla-maxilla; pmx, premaxilla. Line drawing source credit: Angela D. Buscalioni; photograph source credit: Ignacio Arribas.

The unique external **naris** is circular in shape and faces dorsally. Its anterior outline is separated by a short premaxillary vertical process from the anterior border of the snout. The anterior and lateral inner walls of the narial fossa are deep and decreasing in height posteriorly. A small dorsoventral embayment may correspond to the post-internarial fossa, according the description by De Andrade et al. (2011; fig. 6, FoPN) for *G. kiplingi*. A slight perinarial crest borders the posterior area of the naris, and the crest is particularly evident at the left side in dorsal aspect. The ventral premaxilla was covered by an osteoderm that was removed to verify the foramen closure. The area is not clear, but the **foramen incisivum** is closed or might be a narrow slit. The foramen incisivum closure is clear in *H. plotos* (AR-1-5762).

The **suborbital fenestra** is not complete, its anterior maxillary contour is ample and the palatine bar long; thus, the suborbital fenestra would be ellipsoidal and longer than wide. The shape of the fenestra has been reconstructed, based on the preserved contact between the left palatine and maxilla, and the suborbital fenestra is as wide as the palatine bar. The anterior border is far posterior to the last preserved maxillary teeth. The anterior edge is formed by the maxilla and the palatines constitute the medial sides.

The **supratemporal fenestrae** are subcircular openings. The fossa is twice the diameter of the fenestra (37 and 19 mm, respectively). The fossae are level with the skull table surface. The parietal lateral descending process is transversely expanded and forms a wide unornamented medial wall at the supratemporal fossae; the anterior border is not preserved. The parietal contacts the quadrate at the ventral edge of the supratemporal fossa; the suture does not surpass anteriorly the dorsal contact between parietal and frontal.

The borders of the **post-temporal fenestra** are identifiable at the supratemporal fossa and posteriorly at the supraoccipital. The fenestra is posteriorly reduced, surrounded by the supraoccipital ventromedially, by the parietal dorsomedially and by the squamosal dorsolaterally. At the supratemporal fossa, the post-temporal fenestra is surrounded by the squamosal that forms a lateral notch, and by the parietal medially. The quadrate does not form the floor of the temporal canal, but it is placed at the anterior and lateroventral part of the fenestra.

The **trigeminal foramen** is externally bordered by the laterosphenoid anteriorly and the pterygoid process of the quadrate posteriorly.

The **cranioquadrate passage** is opened laterally and ventrally, placed between the quadrate and exoccipital. In lateral aspect (at the side in which the quadrate branch is detached in the fossil) a sulcus is observed. The sulcus is ventral to the lateral lamina projected by the squamosal, and dorsal to an inner extension of the medial part of the quadrate posterior branch. The sulcus reaches the otic recess.

The **foramen magnum** is dorsolaterally bounded by the exoccipitals and ventrally by the basioccipital. The shape of the foramen magnum is elliptical, but it has been widened transversely due to taphonomic distortion. The median pharyngeal tube has a huge foramen located between the basisphenoid and the basioccipital on the ventral region of the occipital area. This wide foramen has a vertical orientation. The foramina of the pharyngotympanic tubes are small, inset and dorsally located at the basioccipital tubera. A large foramen of the median pharyngeal tube is common in other goniopholidid genera (*Eutetrauranosuchus*, *Amphicotylus*, *Anteophthalmosuchus*).

The **choana** has a complex shape. It is framed by a bony rim posteriorly and laterally. The rim is formed posteriorly by the fused pterygoids. Sagittally, an acute medial process of the pterygoid (not completely preserved) would form the choanal septum, and at both sides two elongated depressions are exposed.

### Skull osteology

The anterior margin of the **premaxilla** is almost vertical in profile. The premaxilla has an axe-shaped dorsal contour (a wide transverse expansion but short in length), like *Goniopholis, Hulkepholis* and *Amphicotylus*. The premaxilla-maxillary contact has a wide notch, with an ample semicircular contour, to receive enlarged dentary teeth. The anterior edge of the notch (premaxilla) projects laterally (Figs. 5 and 6). The lateral margin of the premaxilla, dorsal to the notched area, is pitted by vascular foramina. The dorsal suture with the maxilla begins at the posterior third of the notch and extends posteromedially toward the midline of the rostrum. The suture has a concave lateral profile. Ventrally, the premaxilla-maxillary suture projects a short transverse process but medially the maxilla anterior process extends rostrally. The anterior contour of the premaxilla lacks a mid-anteronarial notch, which is unlike *G. kiplingi*. The nasals are excluded from the naris. The posterior dorsal mid process of the premaxilla reaches the third maxillary tooth although this part is not clearly preserved (Fig. 6).

The **maxilla** is preserved as three fragments. The anteriormost part contacts the premaxilla (CBP-839); the mid-portion comprises the maxilla between the fourth and tenth teeth (CBP-836), and the third corresponds to the posteriormost region containing the maxillary depressions (CBP-831). The lateral edge of the anterior portion of the maxilla is inclined posterolaterally. This contour does not show a pronounced dorsal or lateroventral festooning of the maxilla at the area of the large third, fourth and fifth teeth. However, this area is distorted by compression and the alveolar margin is slightly turned inward (Fig. 5). In CBP-836 specimen, the suture maxillonasal is preserved as a narrow band visible on the anterior part of the right maxillary. Isolated nasal fragments are visible on the left side. Accordingly, the nasomaxillary contact is interpreted, as a long, straight suture widened posteriorly (Fig. 6). In ventral aspect, the anterior part of the maxilla forms a flat secondary palate (Fig. 5).

**Figure 7:**
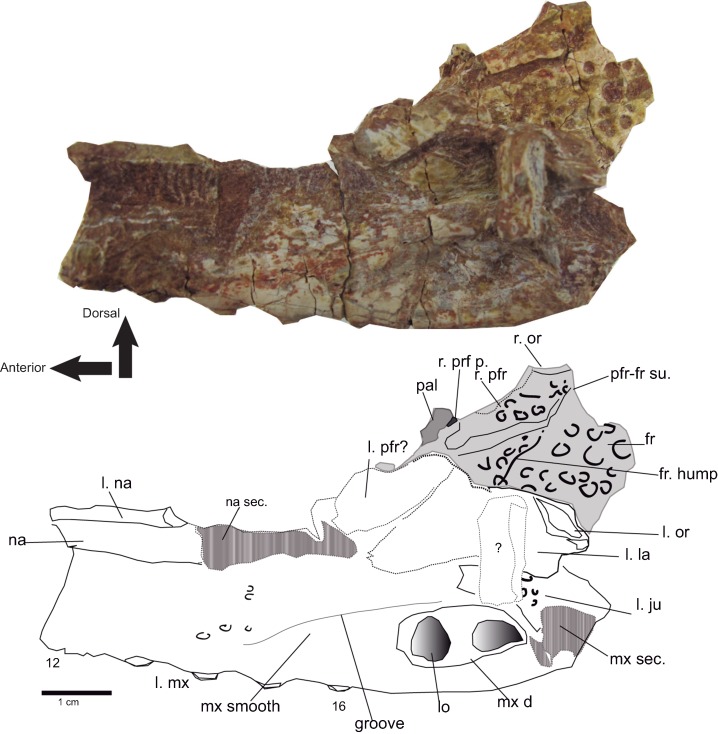
Rostral and orbital region. Lateral view of the left maxilla of *Hulkepholis rori* (CBP-831) from Galve (Teruel). Bones of the interorbital region are in plane gray, and the broken areas have been patterned as gray bars. Abbreviations: fr, frontal; ju, jugal; la, lacrimal; l, left; lo lobule at the maxillary depressions; mx d, maxillary depressions; na; nasal; or, orbital edge; pal, palatine; pfr, prefrontal; pfrp, prefrontal pillar; mx, maxilla; r, right; sec, section; su, suture; the teeth are numbered. Line drawing source credit: Angela D. Buscalioni; photograph source credit: Ignacio Arribas.

The fragment CBP-831 exposes the lateral aspect of the posterior part of the maxilla. The maxillary lateral surface is convex and is vertically oriented. The maxillary ventral margin lacks visible vascular foramina at the alveolar line, and dorsal to the maxillary depressions. The maxilla at that part has a smooth non-sculptured surface. At the level of the seventh tooth the maxillary fossae, a synapomorphy of the family Goniopholididae, is manifest. The maxillary depressions open laterally (Fig. 7), it has a sub-elliptical profile (the anterior part is greater in height than the posterior one), longer than wide, and contains at least two lobes divided by a mid-protuberance. A foramen sits within the anterior lobe of the maxillary fossae. From the anterodorsal area of the depression to the thirteenth teeth, a smooth crest delimits the non-sculptured maxillary ventral border (Fig. 7). Posteriorly, the fragment CBP-831 ends in a broken area above which the jugal suture is laterally disposed. The jugal overlies the maxilla and extends into an anterior process placed dorsal to the maxillary depressions.

**Figure 8:**
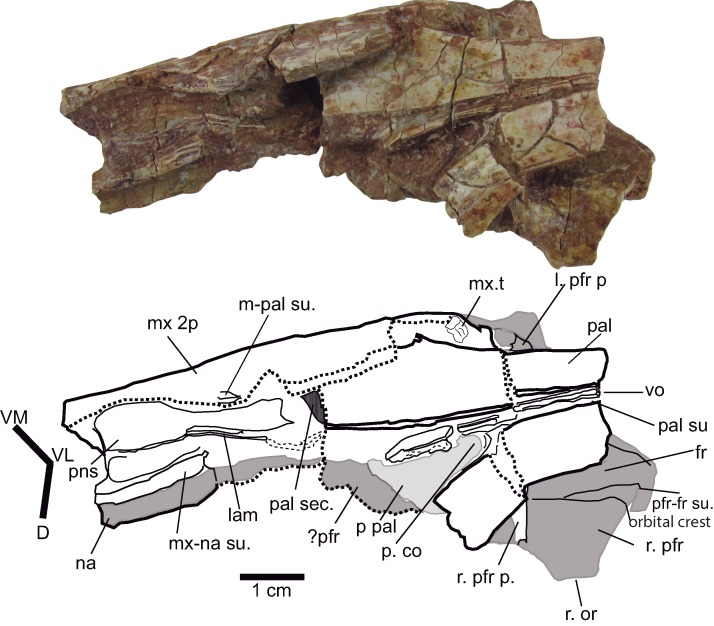
Palatal region. Palate and antorbital paranasal sinus of *Hulkepholis rori* (CBP-831), Galve (Teruel). The axes of the interpretative drawing correspond to the directions ventromedial (VM), ventrolateral (VL), and dorsal (D). The bones in gray are on the dorsal plane. Abbreviations: as in Fig. 6; lam, lamina; mx 2p, secondary maxillary palate; pns, paranasal sinus; p pal, primary palate; p co, primary choana; sec, section; t, tooth: vo, vomer. Line drawing source credit: Angela D. Buscalioni; photograph source credit: Ignacio Arribas.

In ventromedial aspect the maxillary fragment CBP-831 (between the 11th and 14th teeth) exposes the primary maxillary palate (Fig. 8). Internally the maxilla forms the antorbital paranasal sinus (Witmer & Ridgely, 2008). The lateral sinus ends into the lacrimo-nasal cavity whose contour is revealed on a section of the palatine. The sinus has a laminar bone (lam, Fig. 8) that crosses lateromedially toward the nasal cavity. This cavity is topographically similar to the one described for *G. simus* by Salisbury et al. (1999, figs 5, 7) based on the endocast of the rostrum maxillae and orbital region IPB R 359.

In ventral view, CPB-831 exposes the secondary maxillary palate. It has a convexo-concave surface, occupying the alveoli in the concave plane (see dentition). The maxillary lateral contour is moderately undulated and marked by the ample and depressed inter-alveolar spaces. Posteriorly, the maxilla constitutes the anterior border of the suborbital fenestra (Fig. 5C). This border is transversely wide, so that, palatine anterior process is located on the medial palatal surface.

Some fragments of the left **nasal** are detached and uplifted exposing a slightly concave ventral surface (Fig. 7, CBP-831). The **prefrontals** are damaged; the right one is articulated with the frontal, and the left one with the lacrimal; both prefrontals preserve their pillars in connection with the palatines. In dorsal aspect, the suture with the frontal is straight anteriorly but bends laterally at the posterior end (Fig. 7). The preserved orbital rim of the right prefrontal indicates that it contributes to the medial margin of the orbit. The ventral suture fronto-prefrontal is located throughout a broad medial surface at the orbital margin (Fig. 8).

Both prefrontal pillars are turned and medially directed forming an angle less than 90°, as occurs in *H. plotos*, *Anteophthalmosuchus escuchae* and AR-1-3422 and as described in Dollo’s *Anteophthalmosuchus* specimen IRSNB R47 (Martin, Delfino & Smith, 2016). The connection with the palatine process of the pillars is solid. The base of the pillars is not high, and laminar in shape (Fig. 8).

The **lacrimal** surface is dorsolateral and not heavily sculptured in CBP-831. It seems wider than long (Fig. 7), but lacrimal sutures with the maxilla and the nasal are blurred. Its anteriormost tip ends in a conspicuous edge, which is apparently separated from the prefrontal. According to our interpretation (Fig. 7) the anterior lacrimal tip is level with the anterior margins of the prefrontal and maxillary depressions. The posterior lacrimal margin has an elevated rim at the orbital area, placed dorsally to an orbital notch. The lacrimal orbital rim shows a marked depression that extends transversely. In turn, the notch marks the lacrimojugal suture. These two features, the presence of a notch in front of the orbit, and the depression at the orbital rim are also observed in *H. plotos*. The position of the lacrimal notch corresponds to the lacrimal fossa of *G. kiplingi* (De Andrade et al., 2011, FoLac, figure 6), although in *Hulkepholis* is transversely broad.

The dorsal surface of the **frontal** is flat; and an interorbital crest is absent. The frontal presents a slight swelling on the medial plane (Fig. 7), placed level with the prefrontal pillars, and in continuity to the oblique part of prefrontofrontal suture. The swelling becomes evident by a change in the ornamentation, and it is similar in *H. willetti* and *H. plotos*. The dorsal surface of the frontal is ornamented by pits, which disappear in front of the swelling. In ventral view, the frontal is concave where the olfactory tract would have been situated.

**Figure 9:**
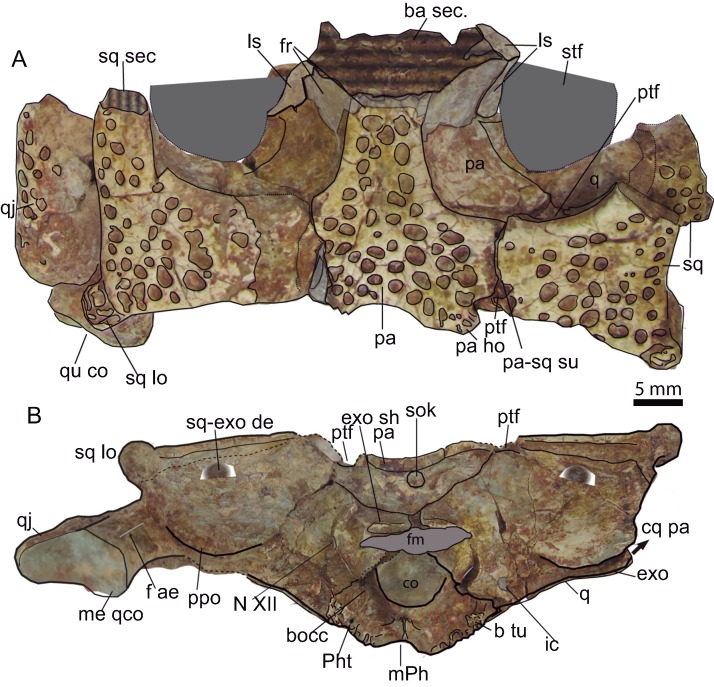
Skull table and occipital region. (A) Dorsal aspect of the skull table of *Hulkepholis rori* (CBP-385) from Galve (Teruel). The skull table has been backwardly tilted to expose bones of the supratemporal fossae. (B) Occipital region. Abbreviations: ba, basicranium; b tu, basioccipital tubera; bocc, basioccipital; cq pa, cranioquadrate passage; co, occipital condyle; exo sh, exoccipital shelf; f ae, foramen aërum; fm, foramen magnum; fr, frontal; ho, hornlet; ic, posterior carotid foramen; ls, laterosphenoid; m Ph, median pharyngeal tube; me qco, medial quadrate condyle; N XII, hypoglossal formen; pa, parietal; Ph t, pharyngotympanic tube; ppo, paroccipital process; ptf, posttemporal fossa; q, quadrate, qj, quadratojugal; sec, section; sq, squamosal; sq-exo dep, squamosal exoccipital depression; sq lo, squamosal lobe; sok, supraoccipital knob; stf, supratemporal fenestra; su, suture. Line drawing source credit: Angela D. Buscalioni; photograph source credit: Ignacio Arribas.

The posterior region of the frontal is preserved in the piece (CBP-385) that contains the suture with the parietal and the laterosphenoid. The frontoparietal suture is straight and positioned on the anterior third of the supratemporal fossae. The frontoparietal suture at the supratemporal fossa has an anterior concave outline, and it is placed posterior to the suture at the intertemporal bar (Fig. 9). The frontal contacts the laterosphenoid in a synarthrosis suture, overlying the laterosphenoid medially.

A fragment of the left anterior process of **postobital** (CBP-830) is recognizable as a subtriangular non-ornamented long and pointed process, with a concave and deep inner face. The outer border is sharp and convex.

The **parietals** are fused sagittally. However, unfused parietals appear in other goniopholidids: in AR-1-3422 they are paired at least at the posterior end of the skull table; also in *H. plotos* (Fig. 2) the frontals and parietals maintain their mid suture, and *Amphicotylus stovalli* (OMNH 2392) has unfused frontals. Allen (2012, p. 7) writes: “The [frontal] caudal process features a deep groove corresponding to the midline suture flanked by the raised medial margins of the supratemporal fenestrae.” The parietal has a “T” shape, anteriorly narrow, differing from the subrectangular parietals of *Eutetrauranosuchus delfsi* (Pritchard et al., 2013). Anteriorly, the intertemporal bar is long, and constitutes two-thirds of the total length of the supratemporal fossa. The parietal intertemporal bar lacks a sagittal crest. The parieto-squamosal suture is laterally placed, and has a slightly curvilinear outline, with the convexity toward the parietal.

Posteriorly, the parietal forms the posteromedial region of the skull table, excluding the supraoccipital from a dorsal exposition. The contact parieto-supraoccipital is visible on the occipital region. The parietal has a mid-concave posterior edge, and two parasagittal hornlets overhanging the occipital area. These hornlets are sculptured with tiny pits (Fig. 9).

The **squamosals** form the posterolateral corners of the skull table and each contribute to 1/3 of the posterior skull table width. The squamoso-postorbital suture must be placed anterior to the anterior third of supratemporal fossa, so that the squamosal is at least twice longer than it is wide. The dorsal surface of the squamosal is flat. The squamosal posterior margin is almost straight, and forms a sharp rim, demarcating the bone on the occipital surface. A buttressed lobe projects caudolaterally, passing over the paroccipital border. The lobe has a corrugated ornamentation (Fig. 9) and is delimited anteriorly by a sulcus. A separate lobe is also present in *H. plotos* (Fig. 2) but in this species the squamosal lobe is ornamented by pits. Anterior to the lobe, the squamosal is laterally concave, but it becomes slightly convex toward the middle of the skull table giving a sinusoidal appearance to the squamosal lateral profile. A groove demarks the ornamented dorsal surface of the squamosal from its lateral border. The squamosal is dorsoventrally narrow and tapers dorsomedially on the occipital surface. At the occipital areas, the ventral surface of the squamosal is anteriorly inclined (Fig. 9), and a transverse shallow crest, dorsally curved, delimits its suture with the exoccipitals. A squamosootoccipital depression at the contact with the exoccipital is similar to that described in *G. simus* (Salisbury et al., 1999) but the presence of a foramen is not clear. Posterolaterally, at the otic area, the squamosal projects a ventral lamina, which anteriorly shapes the dorsoposterior curvature of the otic recess, and ventrally the limit of an open cranio-quadrate passage.

The posterior process of the left **quadratojugal** is an elongated bone mostly exposed laterally. Its dorsal surface is convex whereas the ventral surface is strongly concave. The quadratojugal covers the mid-lateral surface of the quadrate branch on an extended suture. This suture does not reach the quadrate condyle.

The **quadrate** is a transversely expanded bone solidly attached to the braincase. However, the posterior body of the quadrate is short and placed in line with the occipital condyle. The orientation of the posterior process of the quadrate is scarcely bent ventrally, and it is posterolaterally directed. The dorsomedial surface of the posterior articular ramus is convex, and laterally bends toward the dorsoventrally thin quadratojugal. The posterior process has a subtriangular section with a dorsal conspicuous ridge at its cranial third. The condyles are slightly asymmetrical; the medial hemicondyle extends ventrally beyond the edge of the articulation and it is larger than the lateral one. A mid-groove divides the condyles (Fig. 9).

**Figure 10:**
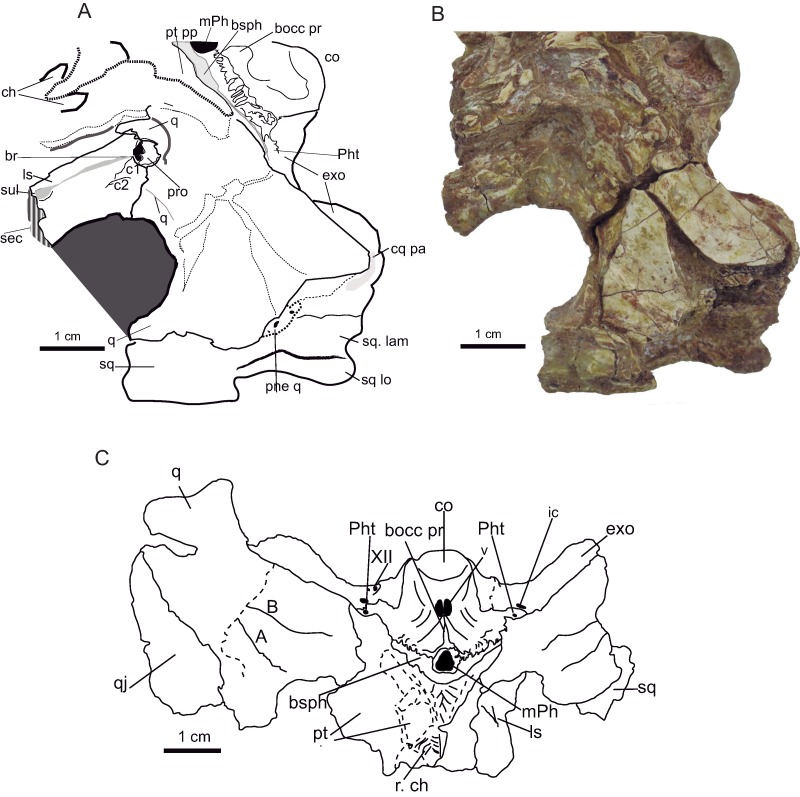
Basicranium. *Hulkepholis rori* (CBP-385) from Galve (Teruel). (A) Line drawing in lateroventral aspect (B) Photograph. (C) Line drawing in ventral aspect. Abbreviations: A and B, quadrate ventral crests; booc pr, basioccipital protuberance; bsph, basisphenoid; br, laterosphenoid bridge for N V1; c 1 and c2, crests of laterosphenoid; ch, choanae; co, occipital condyle; cq pa, cranioquadrate passage; exo, exoccipital; ic, posterior carotid foramen; ls, laterosphenoid; m Ph, median pharyngeal tube; pne q, pneumatization at quadrate; Ph t, pharyngotympanic tube; pro, prootic; pt, pterygoid; pt pp, pterygoid posterior processes; q, quadrate; qj, quadratojugal; r, right; sec, section; sq, squamosal; sq lo, squamosal lobe; sq lam, lateroventral lamina of squamosal at otic area; sul, sulcus; v, vein passage; XII, hypoglossal foramen. Line drawing source credit (A): Angela D. Buscalioni; (C); Ignacio Arribas; photograph source credit: Ignacio Arribas.

Ventrally the quadrate is smoothly textured, except at the posterior part (near the condyles) that is reticulated with pitting (Fig. 10); this is also observed in *Anteophthalmosuchus escuchae*. The two crests corresponding to A and B according Iordansky (1973) outline a raised area. The crests’ arrangement is like that of *Eutretauranosuchus* (Pritchard et al., 2013). The A lateral crest spreads anteromedial in parallel with the quadratojugal suture. The B crest is sharp and placed between the quadrate and the pterygoid, and ends close to the lateral condyle posteriorly, at the level of the A crest. In the holotype of *Anteophthalmosuchus escuchae* the B crest is thick, but the A crest is weak, possibly also due to preservation.

A section of the quadrate is visible just posterior to the otic recess; the inner quadrate shows an ample air passage medially placed surrounded by other smaller conducts (Fig. 10). The quadrate is hollow, and the cavities are connected to the middle ear forming part of the suspensorium diverticula (Dufeau & Witmer, 2015).

The anteroventral process of the quadrate (dorsal primary head) reaches posteriomedially the pterygoid and overlaps the basisphenoid posteriorly (Fig.10). The pterygoid descending process of the quadrate is anteroposteriorly long but dorsoventrally low. An anterior projection of the quadrate touches the anteroventral ramus of the laterosphenoid (Fig. 10). The quadrate pterygoid process forms the border of the foramen ovale ventroposteriorly, and dorsoanteriorly it contacts the prootic. The prootic is overlain by the quadrate but exposed laterally and placed posterior to the trigeminal foramen. The prootic occupies most of the diameter of the fossa (Fig. 10).

The dorsomedial part of the primary quadrate head contacts laterosphenoid, this contact forms a tubercle of the medial margin of the supratemporal fossa (Fig. 10). The contact reaches the dorsal edge of the foramen ovale. The quadrate forms the ventral part of the supratemporal fenestra and part of the fossa. The suture with the laterosphenoid rises dorsally from the quadrate-laterosphenoid tubercle. The quadrate contacts the parietal medially, so that the fossa wall is divided into two equivalent longitudinal areas by this suture. The quadrate curves laterally and contacts ventrally the squamosal (Fig. 10).

The **supraoccipital** has a triangular profile; it is almost vertical, differing from *G. baryglyphaeu*s, which has an inclined supraoccipital (Schwarz, 2002). At the center, there is a dorsal knob, but a median crest is absent (Fig. 9). The supraoccipital bears two horizontal parasagittal projections that are posteriorly directed. The supraoccipital shapes the ventrolateral edge of the post-temporal fenestra, whereas the squamosal constitutes the dorsolateral corner and the parietal the dorsomedial one. The contour of the ventral suture with the exoccipitals is rather sub-rounded but has a conspicuous mid-ventral convexity.

The **exoccipital** contacts the squamosal dorsally, the supraoccipital medially, and the quadrate ventrally. The paroccipital process is a lamina posterolaterally oriented, with a curved posteroventral contour. Ventrolaterally, the paroccipital process overhangs the quadrate. The exoccipitals form part of the dorsal margin of the foramen magnum; they develop a wing-like projection over the foramen magnum excluding the supraoccipital. The occipital condyle is framed by parasagittal robust exoccipital pedicels as occurs in *Goniopholis*, and as described in the specimen IRSNB R47 by Martin, Delfino & Smith (2016), *H. willetti* and the goniopholidids from Ariño. The cranial hypoglossal nerve (pair XII) and the posterior carotid foramen open on the exoccipitals; the posterior carotid foramen laterodorsal to the lateral basioccipital tubera, and cranial nerve XII lateral to the exoccipital pedicels of the occipital condyle (Fig 9). The ratio between the foramen magnum and occipital condyle is 1.46 in the Galve specimen.

The **basioccipital** is transversely extended, and the surface inclines anteroventrally. It is subtrapezoidal, the ventral end wider than the dorsal. Dorsally, at the base of the foramen magnum, the occipital condyle is shallowly concave. The occipital condyle has a ventrally turned lip, as seen in lateral view (Fig. 9). Ventral to the condyle there are two parasagittal vascular foramina inset at two depressions distinguished by a mid-ridge (Fig. 10). Ventral to these depressions, a salient protuberance is backwardly directed. This crest is absent in *H. plotos* and *Anteophthalmosuchus escuchae*. The exoccipital does not descend along the lateral basioccipital tubera maintaining the contact dorsal to it. The basioccipital tubera slightly surpass the plane of the dorsal contour of the foramen of the median pharyngeal tube. The lateral tubera have thick corrugated ventral borders, and they fold medially (folding is mentioned also for the specimen R-47; Martin, Delfino & Smith, 2016).

The **basisphenoid** is exposed between the pterygoid and the basioccipital as a thin lamina in ventral view, exposed medially. The basisphenoid forms the anterior edge of the foramen of the medial pharyngeal tube, and the anterior edge of the pharyngotympanic foramen.

**Figure 11:**
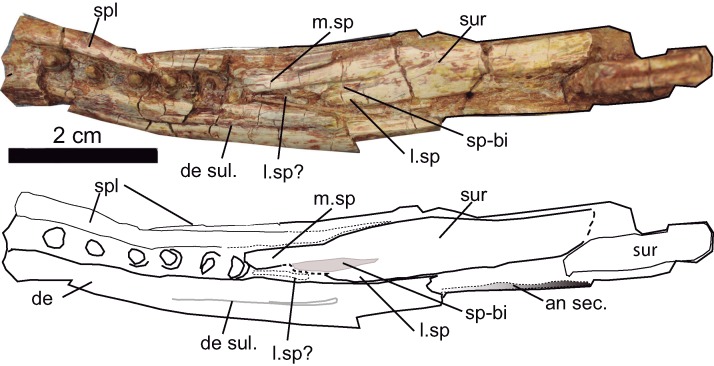
Mandible. Dorsal aspect of the mandible of *Hulkepholis rori* (CBP-383) from Galve (Teruel). Abbreviations: an sec, angular section; de, dentary; de sul., dentary sulcus; m. sp, medial spine of the anterior projection of the surangular; l. sp, lateral spine of the anterior projection of the surangular, (see text for l.sp?); sp-bi, depressed area associated to the bifurcation of the lateral and medial surangular spines; spl, splenial; sur, suarangular. Line drawing source credit and photograph: Angela D. Buscalioni.

The **laterosphenoid** does not preserve the capitate process. It is a conspicuous bone thick anteromedially. It takes part of the anterolateral and anteroventral region of the foramen ovale, and the ventromedial margin of the supratemporal fossa (Fig. 10). A prominent cotylar crest delimits the ventral part from the dorsal one. Its dorsal part corresponds to the area that shapes the supratemporal fenestra. The cotylar crest is like that of *Eutretauranosuchus* (Pritchard et al., 2013). Ventrally, on the posteromedial margin of the supratemporal fossa, a protuberance marks the suture with the quadrate. The laterosphenoid constitutes the anterodorsal margin of trigeminal fossa; the bone on level the fossa is stout and bulky. The laterosphenoid forms a crest that protects the canal of the trigeminal nerve (V1, ophthalmic branch) which extends parallel of the anterodorsal margin of the ascending process of the pterygoid. The crest bifurcates (c1 and c2 in Fig. 11) toward the anteroventral border of the trigeminal fossa.

The **palatines and vomers** are exposed in articulation. The anterior maxillary processes of the palatines are as wide as long, occupying the medial part of the suborbital fenestra. The lateral contact with the maxilla seems to be straight. The process has a convex anterior contour (Figs. 5 and 8). The palatine bar (between the palatal fenestrae) is wide, slightly narrower than the anterior palatine process. In the specimen, the palatines do not suture each other sagittally, they are separated by a pair of thick vertical laminae that extend from the base of the prefrontal pillar to the anterior palatal processes. Nonetheless, the secondary palate does not bear two additional palatal openings, and the absence of the palatine mid-suture and the exposure of vomer are likely due to preservation. The two laminae coincide with the vomeral septum, placed at the same location as *Eutretauranosuchus delfsi* (Pritchard et al., 2013) and *Amphicotylus lucasii* (Mook, 1942). An elongated depression (visible due to the displaced palatine right branch) with a curved posterior border matches topographically with the primary choana (Fig. 8).

The **pterygoid** is broken and crushed, and displaced but the posterior margin of the secondary choana is well delimited. The posteromedial margin of the pterygoid is concave and two posterolateral processes project up to the base of the pharyngotympanic tubes. An isolated detached fragment of the left pterygoid wing (CBP-8310) shows that the pterygoid flange would be anteriorly thick, and posteriorly acute and thin. The dorsal surface of the wing is concave, and the ventral surface is convex. On the dorsal surface of the pterygoid wing, the suture with the descending process of the ectopterygoid shows that this bone does not reach the posterior tip, and that the contact extends medially.

### Mandible osteology

The left posterior portion of the **dentary** is preserved together with the last six teeth in situ (Fig. 11). The alveoli are widely disposed, not set on discrete alveoli, and they do not have alveolar collars. The dentary is about 10 mm height at that part of the mandible, and its lateral surface lacks ornamentation. The posterior part of the dentary is a thin laminar bone that has a unique posterior process. The process ends in a truncate tip that sutures dorsally with the surangular and ventrally with the angular. Laterodorsally a dentary sulcus is appreciated from the last fourth tooth toward the rear of the bone. The wall at the medial margin of the teeth is formed by the splenial. Although not clearly preserved, the external mandibular fenestra is closed.

The **angular** is partially preserved. The anterior angular spine reaches the level of the last three teeth. It is stout and ornamented with grooves anteriorly and pits posterolaterally. Its ventral margin is curved. At the medial view, the ventral contour of the internal mandibular fenestra indicates that this opening is anteroposteriorly extended. The **surangular** is broken and incrusted in the space of the internal mandibular space. In CBP-832 is observed a flat anterodorsal spine of that reaches the alveolar margin. The surangular is anteriorly bifurcated, and the lateral ramus is shorter than the inner one; unclear whether the anterior lateral ramus has a thin spine placed laterally to the dental series as was described by Martin, Delfino & Smith (2016). The **splenial** covers the medial side of the dentary series. It is a thick, medially curved bone, which has a ventral contribution to the mandibular ramus. It extends posteriorly up to the base of the medial branch of the surangular spine. The **articular** represented by the fragment CBP-83 11 is an ample glenoid fossa, ventrally stout and short.

### Dentition

The teeth of the premaxillae, maxillae and dentaries are set in individual alveoli, except for the last six mandibular teeth that are set in a common sulcus. The premaxilla (CBP-839) has five ventrally oriented alveoli as in most goniopholidids (not in *Amphicotylus lucasii* and *H. plotos* where the alveoli are slightly lingually turned). The alveolus for the fifth tooth is placed at the posterolateral margin of the premaxilla and anterior to the premaxilomaxillary notch. The diameter of the first alveolus is slightly larger (three mm) than the second, the third and the fourth are four mm, and the fifth is the smallest (two mm), that is 5 < 2 < 1 < 4 = 3. This pattern is shared by *G. kiplingi*. Between the first and second alveolus there is a diastema, and at the base of these two alveoli an enlarged pit receives a dentary tooth. The second and the third alveoli are closely placed. The third and fourth are separated by a diastema that lodges an oblique occlusal pit, which extends from alveolar base to the premaxillary border (Fig. 7).

There are at least 18 maxillary teeth. One of the features that characterize *Hulkepholis* are the large inter-alveolar distances. In *H. rori* sp. nov. ample space is present between all the maxillary teeth, being at least twice the alveolar diameter for the first and for the last teeth, but as wide as the alveoli for the remaining mid maxillary teeth (Figs. 6 and 7). In CBP-839 the second maxillary tooth preserves the crown (four mm in diameter) yet the apex is broken. The third alveolus can be partially distinguished, but the connection with the fourth alveolus is broken. The first complete alveolus of the maxilla fragment CBP-836 corresponds to the sixth tooth. The fourth or fifth teeth would be the largest. The pits derived from the occlusion of dentary teeth are interfingered on the maxilla (placed more lateral in the fifth and sixth teeth than in the subsequent ones).

**Figure 12:**
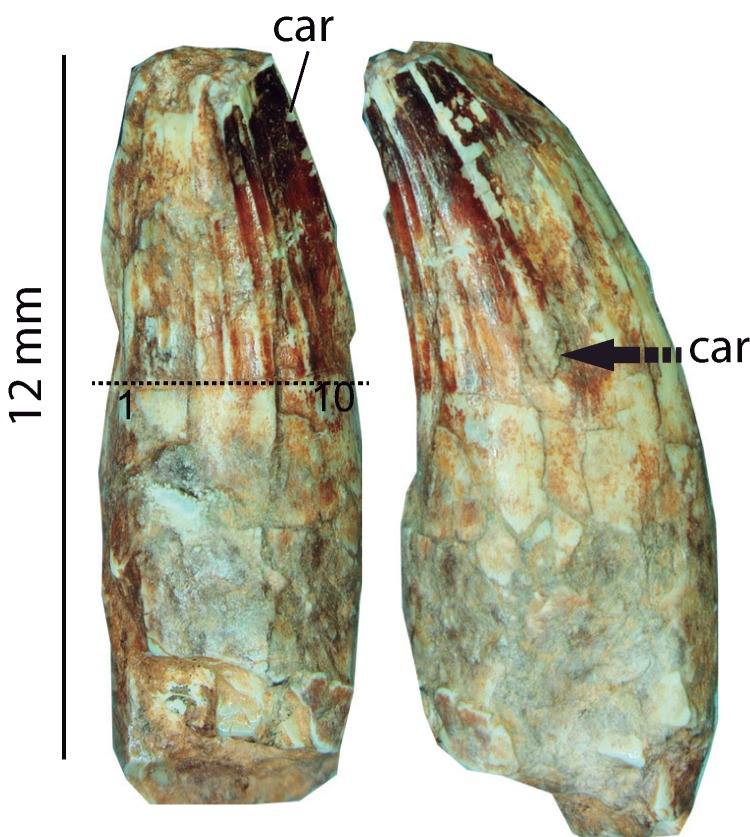
Dentition. Isolated tooth in lingual and mesial-distal views of *Hulkepholis rori* (CBP-833), Galve (Teruel). Abbreviations: car, carena. The arrow points the end of the carena. 1 to 10, ridges of ornamentation on the lingual crown surface. Dotted line is the boundary of the crown. Photograph source credit: Angela D. Buscalioni.

CBP-831 corresponds to the posterior region of the left maxilla, comprising the 12th to 17th teeth. The alveoli are of subequal diameter. The inter-alveolar spaces are ample, and the teeth crowns set in raised alveoli, providing an undulating aspect to the posterior alveolar margin of the maxilla. These raised alveoli have a medial rim. Every inter-alveolar space has an occlusal pit. The teeth crowns are conical with subcircular sections, and the crown-root contact lacks a neck. Teeth are stoutly ornamented by 10 regularly spaced ridges that extent apicobasally (Fig. 12).

## Phylogenetic results and discussion

**Figure 13:**
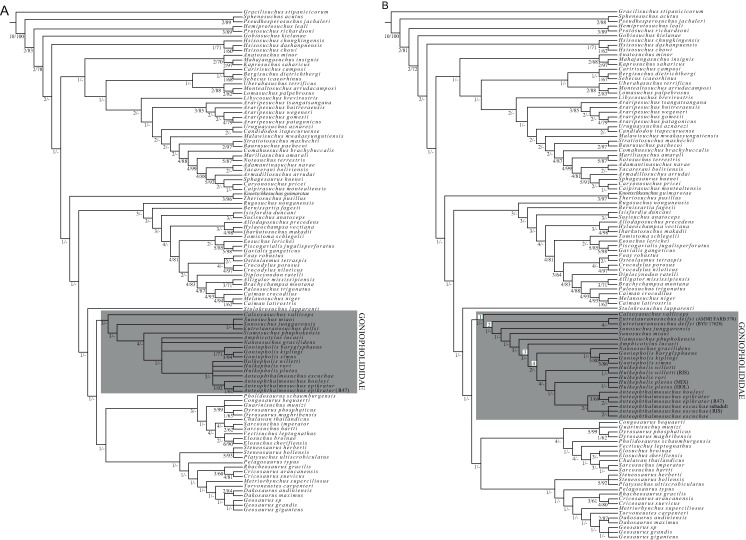
Phylogenetic analyses. (A) Strict Consensus cladogram of nine resulting trees of 2,227 steps, values of consistency index of C = 0.296 and RI = 0.765, conducted on the base of Ristevski et al. (2018) dataset to which only the species *Hulkepholis rori* was added (Dataset S1). (B) Strict Consensus cladogram of two resulting trees of 2,370 steps; values of consistency index of CI = 0.287 and retention index of RI = 0.761. In both cladograms bootstrap resampling method was performed with 1,000 replicates, and the Bremer decay adding 10 extra steps were analyzed (first value in figure corresponds to Bremer decay, bootstrap values <60 are not represented). Diagnoses of the nodes of cladogram B: Node 1 Goniopholididae; #26 (1), ventralmost foramina high on the maxilla and distant to alveoli; #66 (0), naso-oral fossa absent; #86 (1), presence of a lateral fossa next to alveolar margin; #89 (1), presence of maxillary depressions close to the maxilla-jugal contact; #122 (0), distal margin of frontal is medial to the dorsal end of postorbital bars; # 205 (2), presence of an anteroposteriorly elongated maxillo-palatine fenestra; #223 (0), anterior process of the palatines short with length subequal to width; # 245 (1), presence of a interchoanal septum in the ventral surface of the nasopharyngeal duct. Node 2 *Eutretauranosuchus* and the remainder goniopholidids; #13 (0), ornamentation dominated by pits; #31 (2), dorsal orientation of nares; #43 (0), antorbital cavity absent; #44 (0), absence of internal antorbital fenestra; #53 (1), presence of a small and shallow lacrimal fossa; # 80 (1), ventral margin of the maxilla festooned; #105 (1), presence of a shallow fossa at the supratemporal fenestra. Node 3 defined by *Amphicotylus* and the European goniopholidids ((*Nannosuchus* + *Goniopholis*) + (*Hulkepholis* + *Anteophthalmosuchus*)); # 65 (4), profile in dorsal of the premaxillae axe-shaped; #76 (0), laterally concave nasals in dorsal view posterior to external nares; #124 (1), extension of the posterior margin of the skull roof over the occipital surface, present and evident. Node 4 ****defined by all the European goniopholidids; #122 (2), distal margin of frontal is posterior to the dorsal end of postorbital bars; #141 (1), participation of the frontal in the primary medial border of the orbit very restricted; 142 (1), anterior process of the frontal reaches o barely surpass the anterior tip of the prefrontal; #152 (1), presence of a short postorbital anterolateral process projecting. Abbreviations in the cladogram represent different author coding, and specimens: MIX = all the specimens of the Ariño material; HOL= holotypes of the Ariño material; RIS = Ristevski et al., 2018. Line drawing source credit: Ignacio Arribas.

A first phylogenetic analysis was conducted on the base of Ristevski´s dataset to which only the species *H. rori* was added (Dataset S1). Not surprisingly, the phylogenetic result agrees with Ristevski et al. (2018) that Goniopholididae is monophyletic and the sister taxon of Tethysuchia and Thalattosuchia, which together constitute a major unnamed clade (Fig. 13). Nine equally parsimonious trees with a length of 2,227 steps, CI = 0.296, and RI = 0.765 resulted. The node Goniopholididae is herein defined by the same members as Ristevski et al. (2018), with *Calsoyasuchus* the sister taxon of the remaining goniopholidids (Fig. 13A). The strict consensus topology shows a poorly resolved tree with a trichotomy for the clade *Eutetrauranosuchus delfsi*, *Sunosuchus miaoi*, *Sunosuchus junggarensis* (Bremer -1), as the sister group of *Siamosuchus phuhokensis* plus the remaining goniopholidads (Bremer -4). The phylogenetic relationships of *Amphicotylus lucasii* and *N. gracilidens* were not resolved; these taxa form a node (Bremer -1) with the clades *Goniopholis* (Bremer -1) and (*Anteophthalmosuchus* + *Hulkepholis*) (Bremer -3). For the later clade we obtained the same topology as Ristevski et al. (2018, figs 26, 27), that is, *Anteophthalmosuchus* paraphyletic with respect to the formed by *Anteophthalmosuchus escuchae*, *H. plotos*, *H. rori*, *H. willetti*, and the node in which *Anteophthalmosuchus hooleyi*, is the sister taxon to the clade comprised of *Anteophthalmosuchus epikrator* and Dollo’s *Anteophthalmosuchus*. In this cladogram *H. rori* would be a member of the latest European goniopholidids but with an uncertain phylogenetic position.

Due to the unresolved relationships recovered within Goniopholididae, a second analysis was performed including 11 redefined characters (Dataset S2) and adding the specimens listed in Table 1 as terminal OTUs. Two most parsimonious trees were obtained with a length of 2,370 steps, and CI = 0.286, RI = 0.761. Goniopholididae is a fully supported clade (Bremer -1), with *Calsoyasuchus* at the base, and *Eutetrauranosuchus*, *Sunosuchus junggarensis, Sunosuchus miaoi, Siamosuchus*, and *Amphicotylus* as successive sister groups of the two European clades: ((*Nannosuchus* + *Goniopholis*) + (*Hulkepholis* + *Anteophthalmosuchus*)). *Nannosuchus* is herein positioned as the sister taxon of the clade formed by *Goniopholis*, differing from the solution provided by Ristevski et al. (2018). The best phylogenetic support within Goniopholididae corresponds to the clade formed by *Siamosuchus*, *Amphycotylus* and the European lineage (Bremer -3, bootstrap <50%), likewise for each of the two European components (*Goniopholis*, Bremer -3, bootstrap >50%; and (*Hulkepholis* + *Anteophthalmosuchus*), Bremer -4, bootstrap <50%; Fig. 13B).

### Positions of *Hulkepholis* and *Anteophthalmosuchus*

*Hulkepholis* and *Anteophtalmosuchus* differ from *Goniopholis* because they retain the primitive condition of the absence of a prefrontal-lacrimal crest dorsal to the orbit [#99 (0)]; and in the absence of a transverse frontal crest [#101 (0)]. The common ancestor of the clade comprising all species of *Hulkepholis* and *Anteophthalmosuchus* (Fig. 13B) is strongly supported by a set of common unambiguous synapomorphies in the two resulting trees. These synapomorphies are congruent with the shared derived characters described by Ristevski et al. (2018): the supratemporal fossa is larger than the orbit [#107 (2)]; lateral expansion of the frontal arched laterodorsally with palpebral and postorbital curved dorsally [#115 (1)]; posterior ramus of jugal subcircular to subpolygonal in cross-section [#173 (0)]; ventral margin of the jugal at level with the posterior ramus [#178 (0)]; palatal ramus of maxilla is part of the anteromedial border of suborbital fenestra [#215 (1)]. *H. willetti* based on its poorly preservation, it does not share the derived condition of the characters shown in the Iberian species of *Hulkepholis* and the species of *Anteophthalmosuchus*: [#152 (2)] long anterolateral process of the postorbital; [#153 (1)] postorbital process almost reaching the dorsal edge of the anterior jugal ramus, shielding the posterolateral section of the orbit], and [#288 (2)] basioccipital tubera present with lateral edges turned posteriorly.

The incorporation of several individuals of the same species in the analysis (Table 1) enables the resolution of observed interspecific variations as autapomorphies, and the estimation of missing specimen data, thus improving the results and character congruence. The procedure provides the strongest test of monophyly for the larger clade composed by *Hulkepholis* and *Anteophthalmosuchus*. However, despite the present finding that *Anteophthalmosuchus* is monophyletic, this is not the case for *Hulkepholis*, which becomes paraphyletic as *H. willetti* is excluded from the node formed by (*H. plotos* + *H. rori*) (Fig. 13B). The monophyly of *Hulkepholis* is not sustained due to numerous character discrepancies between the Iberian species and *H. willetti* (see Table 4), and such inconsistencies are mostly due to mismatches in the character interpretations (see case B in Table 5). The genus *Hulkepholis* becomes monophyletic (and the sister group of *Anteophthalmosuchus*) when we apply our own character codings and exclude *H. willetti* from the analysis (see Case A in Table 5 for the resulting cladogram). Therefore, a detailed revision of *H. willetti* is necessary to resolve this problem.

**Table 4:** Scores of the characters modified.

Character	*A. epikrator* (holotype)		*A. epikrator* (R47)		*A. escuchae*		*A. hooleyi*		*H. plotos*		*H. willetti*	
	M1	M2	M1	M2	M1	M2	M1	M2	M1	M2	M1	M2
66	1	1	?	?	?	?	?	?	?	0	1/2	?
101	0	0	0	0	0	?	0	0	0	0	0	0
111	1	1	1	1	1	1	3	1	1	3	1	3
139	0	0	0	0	0	?	0	0	0	0	0	0
141	0	0	0	0	0	?	0	1	0	1	0	1
151	1	1	?	?	1	1	1	1	1	1	1	1
155	0	2	0	2	0	0	0	0	0	2	0	2
221	0	0	?	?	?	?	0	0	0	0	0	0
233	?	?	1	1	?	?	1	1	1	1	1	1
247	?	?	?	?	?	?	0	?	0	0	0	0
288	0	2	0	0	0	2	0	2	0	2	0	?

**Note:**

Scores of the characters modified. The matrix only includes the genera *Hulkepholis* and *Anteophthalmosuchus*. M1 corresponds to matrix used in Ristevski et al. (2018), M2 is the matrix that includes the character scores herein modified (see Datasets S1 and S2; Characters S1).

**Table 5:** Monophyly and phylogenetic support of *Hulkepholis*.

Clades (parenthetical notation)	Length and index	Branch support	Bootstrap
Case A. ((*Hulkepholis plotos* MIX, *H. plotos* HOL, *H. rori*) + ((*A. escuchae* RIS, *A. escuchae* HOL, *A. escuchae* SUB) + ((*A. hooleyi* + (*A. epikrator* + *A. epikrator* 47)))))	2,356 CI = 0.288 RI = 0.763	(*Hulkepholis* + *Anteophthalmosuchus*) = −6 *Hulkepholis* = −2 *Anteophthalmosuchus* = −1	(*Hulkepholis* + *Anteophthalmosuchus*) >50% *Hulkepholis* > 82% *Anteophthalmosuchus* <50%
Case B. ((*Hulkepholis willetti* RIS + *H. willetti*) + (*H. plotos* RIS + ((*H. plotos* MIX + (*H. plotos* HOL + *H. rori*)) + ((*A. escuchae* HOL + (*A. escuchae* RIS+ *A. escuchae* SUB)) + ((*A.hooleyi* + (*A. epikrator* + *A. epikrator* 47)))))))	2,372 CI = 0.286 RI = 0.762	(“*Hulkepholis*” + *Anteophthalmosuchus*) = −5 *Anteophthalmosuchus* = −3	(*Hulkepholis* + *Anteophthalmosuchus*) = 50% *Anteophthalmosuchus* = 50%

**Note:**

Monophyly and phylogenetic support of *Hulkepholis*. Case A. Applying the characters coded by us for *H. plotos* and *H. rori* as in Dataset S2, but excluding the taxon *H. willetti* from the analysis. Case B. Combining the characters coded by Ristevski et al. (2018) as in Dataset S1 and by us as in Dataset S2, for *H. willetti* and *H. plotos*. Abbreviations: RIS, Ristevski Dataset S1; HOL, holotypes; SUB, subadult individual.

**Figure 14:**
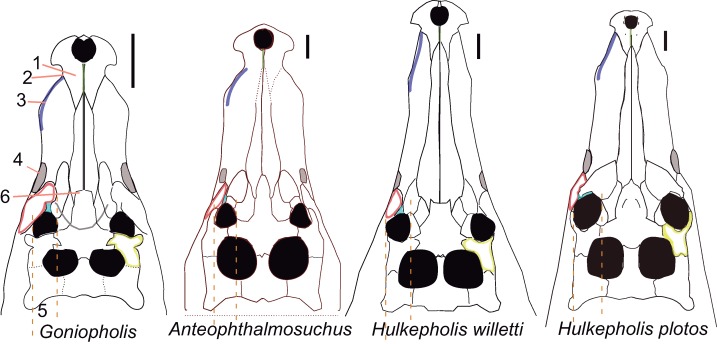
Skull of the European Goniopholididae. Phenetic comparison of the skulls in dorsal view of *Goniopholis kiplingi* (figure modified from De Andrade et al. (2011)), *Anteophthalmosuchus epikrator* (figure modified from Ristevski et al. (2018)) with the genus *Hulkepholis*. *H. willetti* (figure modified from Salisbury & Naish (2011)). The skull of *H. plotos* is a reconstruction based on the holotype and on the specimen AR-1-5762 (see also Fig. 2). The scale bar is five cm. The numbers correspond with the features described in the text. Line drawing source credit: Angela D. Buscalioni.

Nonetheless, phenetic similarities between the Iberian and English *Hulkepholis* are not only based on the snout proportions, configuring a longirostral skull, but also on a set of features that differentiate this genus in comparison with *Anteophtalmosuchus* and *Goniopholis* (Fig. 14). The main similarities between *H. wiletti* and *H. plotos* involve: (1) a relatively short dorsal suture between premaxillae that ends anterior to the maxillary expansion; (2) a short premaxillo-maxillary notch; (3) a linear maxillary lateral contour between the first to the fifth teeth, instead of convex one; (4) a verticalized maxilla so that the maxillary depressions face laterally and is almost hidden in dorsal aspect; (5) orbits facing dorsolaterally, and displaced laterally in the skull; (6) a stout and transversely expanded frontal anterior process.

### Distinctive features of *H. rori*

*Hulkepholis rori* is the earliest member of the genus on the Iberian Peninsula (early Barremian in age) and it is related to the Valanginian species *H. willetti* and the early Albian *H. plotos*. The three species constitute a sub-longirostral lineage and share a rectilinear, slightly divergent maxilla, with the two first teeth spaced, and one large vertical wave restricted to the enlarged teeth (3 < 4 < 5), which are close to each other. By the sixth to 13th the alveoli are subequal in size with wide inter-alveolar spaces, and the alveoli become greatly reduced posteriorly. The maxillary dental series in *H. plotos* (not so clear in *H. rori* and *H. willetti*), is peculiar because the sixth alveolus is as small as the second. The rostral length and the arrangement of the maxillary dentition are different in *Anteophthalmosuchus* (Table 2; Fig. 3). The three *Hulkepholis* species have also in common the anterior projection of a rounded maxillary ventral process which posteriorly forms a transverse premaxillo-maxillary suture, and the shape (rectilinear) and position (anterior) of the fronto-parietal suture. Although *H. rori* is poorly preserved, some diagnostic features, such as the premaxillary dentition, the presence of two foramina set into two divided depressions at the base of the occipital condyle, and a ventral mid-protuberance at the basioccipital, support the proposal for a new *Hulkepholis* species. Thus, *H. rori* has a particular character combination in comparison with its sister species (see also Table 2).

The Galve fossil also shows interesting anatomical features, some of which have not yet been described in other goniopholidids. *H. rori* shows a wide foramen of the medial pharyngeal tube despite its relatively small skull size; the quadrate holds wide internal spaces or fossa. The two traits are linked with cavities in the middle ear and point to a highly pneumatized skull. Pneumatization in Crocodylia has been functionally related to the rapid equalization of air pressure at the lateral and medial sides of the tympanic membranes (Owen, 1850; Colbert, 1946), for better buoyancy control of the skull, and/or to the improved reception of sound (Dufeau & Witmer, 2015; Montefeltro, Andrade & Larsson, 2016; Bona, Paulina-Carabajal & Gasparini, 2017). The function of such a long meatal chamber in goniopholidids is not clear (Montefeltro, Andrade & Larsson, 2016), although the combination of these features (quadrate pneumaticity, width of the foramen for the medial pharyngeal tube, and long meatal chamber), together with the rostral length and the maxillary dental disposal suggest an aquatic lifestyle. Furthermore, this distinctiveness would have been functionally linked to highly specific neck and skull movements, also linked to the protuberant knob and crest placed on the occipital region. The occipital protruding areas, such as the ventral paroccipital edge, the laterodorsal extension of the basioccipital tubera, the depression at the exoccipital-squamosal contact, and the overhanging posterior skull border, together with the diagnostic traits of *H. rori* (i.e., vascular fossa and mid-ventral basioccipital protuberance), indicate that these features would have played an important role in the lateral flexion of the head and neck (Snively & Russell, 2007).

### The geometry of the skulls

**Figure 15:**
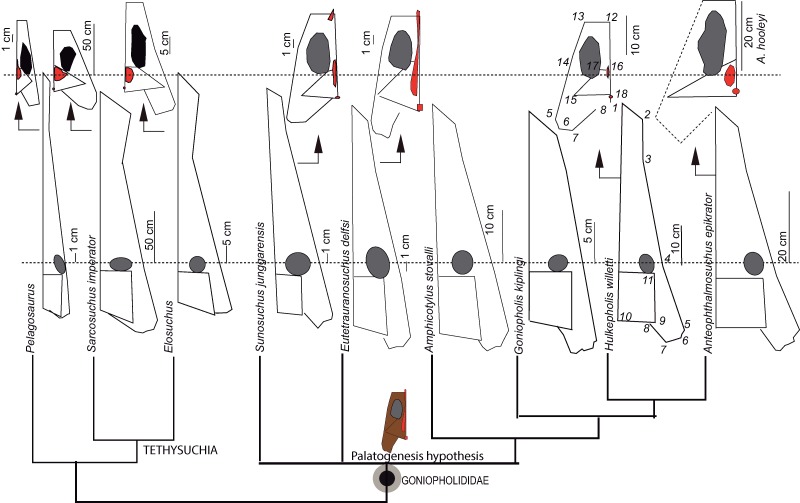
Geometrical comparison of neosuchian skulls. Dorsal and ventral bones of Tethysuchia, Thalattosuchia and Goniopholididae skulls. The landmarks in dorsal view are: (1) premaxillary tip; (2) lateralmost premaxillary edge; (3) maxillary edge at 5th tooth; (4) edge posterior to jugal bar; (5) lateralmost quadratojugal edge; (6) quadratojugal-quadrate suture; (7) quadrate condyle; (8) medial edge of the quadrate; (9) squamosal tip; (10) parietal margin; (11) skull table posterior to orbital edge. A partial clipping of the ventral aspect of the skull has been also depicted and includes the ventral fenestrae and openings. The selected landmarks for the ventral skull are: (12) maxilla-palatine suture at the palate; (13) maxillary orthogonal edge; (14) end of the maxillary dental series; (15) pterygoid lateral and posteriomost tip; (16) palatine-pterygoid suture; (17) palatine-pterygoid suture at the suborbital fenestra; (18) posterior tip at the basioccipital ventral edge. Line drawing source credit: Angela D. Buscalioni.

Interestingly, the geometric comparison of the skull (Fig. 15) shows major differences between the species of goniopholidids, tethysuchians and the thalattosuchian *Pelagosaurus*. The postrostral module in Goniopholididae tends to be relatively enlarged. This trend reaches its maximum in the complex of the European species because the laterodorsal expansion of the suspensorium (quadratojugal and quadrate) is outstanding (especially in *Hulkepholis* and *Anteophthalmosuchus*; Fig. 15). In addition, the presence of a singular palatal cleft, the narrowness of the secondary choana, and the wide diameter of the foramen of the median pharyngeal tube indicate major goniopholidid modifications when compared to *Pelagosaurus*, *Sarcosuchus* and *Elosuchus* (Fig. 15).

In basal goniopholidids, such as *Eutretauranosuchus delfsi*, *Amphicotylus lucasii* and *Amphicotylus stovalli*, the primary choana opens on the palate (Pritchard et al., 2013; 271), whereas in the derived European genera the nasopharyngeal duct is completely closed and the secondary choana opens between the palatines and the pterygoids (De Andrade et al., 2011). Nevertheless, we have yet to understand whether the aperture of the primary choana in the palate has phylogenetic significance, or whether it is an evolutionary novelty of Goniopholididae connected with a singular palatogenesis.

In the case of the phylogenetic hypothesis, the common ancestor of Tethysuchia, Thalattosuchia and Goniopholididae should have had a nasopharyngeal tube leading from the primary choana to the secondary one (the same condition as *Eutretauranosuchus*) with posteriorly fused pterygoids (Pritchard et al., 2013). In fact, features such as the vomer exposition at the palatal surface between the maxilla and the palatine, and a high the degree of variation of the choanal shape and disposition have been figured and described among members of Thalattosuchia (Wilberg, 2012). These features should be reviewed for the earliest members of Tethysuchia to test whether such variability would have preceded the closure of the primary choana, prior to the full formation of the secondary palate in Neosuchia—referred to by Dollman et al. (2018) as the stage of the palatine secondary palate.

The palatogenesis hypothesis would have implied a fused palatine portion of the secondary palate in the common ancestor of (Tethysuchia + Thalattosuchia), but also an exclusive goniopholidid evolutionary novelty that would have promoted the formation de novo of a palatal cleft (i.e., the presence of a palatal fenestra) (Fig. 15). Palatogenesis occurs during embryogenesis to form the secondary palate, separating the oral cavity from the narial passage (Lan, Xu & Jiang, 2015). The most noticeable modification in palatogenesis involves different types of palatal clefts that can be caused by major mutant genes in mammals (Levin, 1963; Smahel & Müllerová, 1986; Gregory, 2000) and reptiles (Bellairs & Boyd, 1957; Ferguson, 1979). In turn, alterations in palatogenesis involve a cascade of osteological changes in the craniofacial configuration, modifying the maxilla, the nasal cavity and the connection between the ear and the palate, whose changes consist of a less deep maxilla, backward maxillary displacement, in tooth malocclusion, widening of nasal cavity and interorbital distance, and variation of the pharyngeal tube width. Thus, in addition to a palatal fenestra other features such as, the extreme platyrostry, vomers located superficially in the palate (contrary to the common condition in Recent crocodiles with deep vomers; Ferguson, 1979), as well as the wide foramen of the median pharyngeal tube, and the backward projection of ectopterygoids and the posterior pterygoid processes, could had been due to a modification in the palatogenesis in Goniopholididae.

## Conclusions

The European lineages of goniopholidids comprise two clades (*Nannosuchus* + *Goniopholis*) plus (*Hulkepholis* + *Anteophthalmosuchus*). The second clade is supported by numerous synapomorphies including a unique set of characters differing from those in *Goniopholis*, denoting the disparity in skull shapes of the European lineage, as varied as in the modern clade of *Crocodylus*. *Anteophthalmosuchus* is clearly defined as a monophyletic group that includes *Anteophthalmosuchus escuchae*, whereas *Hulkepholis* has unstable group membership. However, new contributions would be necessary to better describe *H. willetti* and it would be appropriate to prepare and study newly available Ariño material of *H. plotos*. *H. rori* is based on an incomplete specimen from the locality of Galve (Camarillas Formation, lower Barremian). Nevertheless, the new species raises unexpected questions on the evolution of Goniopholididae. New characters have been recognized in the organization of the palate and in features that shape the occipital region of the skull. The set of palatal characters (the variability in the choana disposition, the extreme platyrostry in which the vomers are superficially located in the palate, the widened of the median pharyngeal tube foramen, the retroverted ectopterygoids, and the backward projection of the posterior pterygoid processes) are discussed as being part of a singular palatogenesis in Goniopholididae. Still, further questions must be answered in order to understand the biomechanics of neck and skull movements, as well as the hearing ability of the aquatic goniopholidid *Hulkepholis*.

## Supplemental Information

10.7717/peerj.7911/supp-1 Supplemental Information 1 List of characters used.

10.7717/peerj.7911/supp-2 Supplemental Information 2 Data matrix 1.

10.7717/peerj.7911/supp-3 Supplemental Information 3 Data matrix 2.

## Institutional acronyms

AMNH FARB: American Museum of Natural History; Fossil Amphibians, Reptiles, and Birds Collection (New York, NY,USA)
AR: Ariño collection in Museo Aragonés de Paleontología, Fundación Conjunto Paleontológico de Teruel-Dinópolis (Teruel, Spain)
BMNHB: Booth Museum of Natural History collections (Brighton, UK)
BYU: Brigham Young University (Provo, UT, USA)
CBP: Cabezo Santa Bárbara, Unidad de Paleontología, Universidad Autónoma de Madrid (Madrid, Spain)
CMNH: Cleveland Museum of Natural History (Cleveland, OH, USA)
DORCM: Dorset County Museum, Dorchester (England)
IRSNB: Institute Royal des Sciences Naturelles de Belgique (Brussels, Belgium)
IVPP: Institute of Vertebrate Paleontology and Paleoanthropology, Academia Sinica (Beijing, People’s Republic of China)
IWCMS: Isle of Wight County Museums Services (Dinosaur Isle Museum and visitor attraction) (Sandown, UK)
MNHN: Museum National d’Histoire Naturelle (coll. Gara Samani. SAM) (Paris, France)
MNN: Musée National du Niger
NHMUK: Natural History Museum (London, UK)
Nr: Teylers Museum (Haarlem, Netherlands)
OMNH: Oklahoma Museum of Natural History (Norman, OK, USA).

## Additional Information and Declarations

### Data Availability

The following information was supplied regarding data availability: The raw character-states are available in the Supplemental Files, used for the phylogenetic analysis in TNT v. 1.1. Material described in the article are stored in Museo Aragonés de Paleontología/Fundación Conjunto Paleontológico de Teruel-Dinópolis (Teruel, Spain) and listed in the Supplemental File.

### New Species Registration

The following information was supplied regarding the registration of a newly described species: Publication LSID: urn:lsid:zoobank.org:pub:A8446014-A73D-4D47-88B7-2EADECC81F32. *Hulkepholis rori* sp. nov LSID: urn:lsid:zoobank.org:act:E2EA1AFF-DDA3-42E3-912F-72A633FA8A0A.
