# Supplementary material for: A new goniopholidid crocodyliform, Hulkepholis rori sp. nov. from the Camarillas Formation (early Barremian) in Galve, Spain)
Source: PeerJ. 2019 Oct 31;7:e7911. doi: 10.7717/peerj.7911 (PMC6825746; doi:10.7717/peerj.7911)
Supplement: Supplemental Information 1 [file peerj-07-7911-s001.docx]

**Supplementary Information**

**The Iberian *Hulkepholis* and *Anteophalmosuchus*: a new goniopholidid species from the Camarillas Formation (early Barremian) in Galve (Teruel, Spain)**

Ignacio Arribas^1^, Ángela D. Buscalioni^1^*, Rafael Royo-Torres^2^, Eduardo Espílez^2^, Luis Mampel^2^ & Luis Alcalá^2^

Ignacio Arribas ^1^, Ángela D. Buscalioni^1^, Rafael Royo-Torres^2^, Eduardo Espílez ^2^, Luis Mampel ^2^, Luis Alcalá ^2^

^1^ Departamento de Biología, Paleontología, Universidad Autónoma de Madrid, Cantoblanco, 28049 Madrid, Spain

^2^ Fundación Conjunto Paleontológico de Teruel-Dinópolis, Museo Aragonés de Paleontología, 44002 Teruel, Spain

Corresponding Author:

Ángela D. Buscalioni^1^

Darwin, 2, Cantoblanco, Madrid, 28049, Spain

Email address: [angela.delgado@uam.es](mailto:angela.delgado@uam.es)

**Material studied**

**Ariño Locality**:

*Hulkepholis plotos*

**AR-1/56 (holotype)**: partial skeleton deposited at Museo Aragonés de Paleontología/Fundación Conjunto Paleontológico de Teruel-Dinópolis (Teruel, Spain), composed by skull (AR-1-2045), 3 vertebrae (AR-1-2048, AR-1-4859, AR-1-4860), a rib (AR-1-2046), a metapodial (AR-1-2048), 3 osteoderms (AR-1-2049, AR-1-4861, AR-1-4862).

**AR-1/2:** partial skeleton deposited at Museo Aragonés de Paleontología/Fundación Conjunto Paleontológico de Teruel-Dinópolis (Teruel, Spain), composed by snout and mandible (AR-1-1625), ten skull fragments (AR-1-1629, AR-1-1640, AR-1-1641, AR-1-1651, AR-1-7287, AR-1-7288, AR-1-7291, AR-1-7292, AR-1-7302, AR-1-7303), a dentary fragment (AR-1-1639), a angular fragment (AR-1-1634, AR-1-7289), six insolated teeth (AR-1-7281 to AR-1-7286), two cervical ribs (AR-1-1661, AR-1-7290), 2 dorsal vertebrae (AR-1-1632, AR-1-1633), 16 vertebrae (AR-1-1626, AR-1-1630, AR-1-1642, AR-1-1658, AR-1-1666, AR-1-1669, AR-1-1670, AR-1-1704, AR-1-7304 to AR-1-7311), a rib (AR-1-1703), 17 rib fragments (AR-1-1647, AR-1-7293 to AR-1-7301, AR-1-7315, AR-1-7316, AR-1-7317, AR-1-7318, AR-1-7319 to AR-1-7321), two partial humeri (AR-1-1649, AR-1-1650), pelvic girdle bone (AR-1-1652), partial femur (AR-1-1654), three metapods (AR-1-1657, AR-1-1662, AR-1-1665), a phalanx (AR-1-1646), 20 dermal armor plates (AR-1-1627, AR-1-1631, AR-1-1635, AR-1-1636 to AR-1-1638, AR-1-1643 to AR-1-1645, AR-1-1667, AR-1-1668, AR-1-7322 to AR-1-7330) and undetermined bones and fragments (AR-1-1628, AR-1-1648, AR-1-1653, AR-1-1655, AR-1-1656, AR-1-1659, AR-1-1660, AR-1-1663, AR-1-1664, AR-1-1705, AR-1-1706, AR-1-7312 to AR-1-7314, AR-1-7331 a AR-1-7340),

**AR-1/104:** partial skeleton deposited at Museo Aragonés de Paleontología/Fundación Conjunto Paleontológico de Teruel-Dinópolis (Teruel, Spain), composed by dorsal skull bones (AR-1-5762) (excluding neurocranium and mandibles), a maxilar fragment (AR-1-8553), eight skull fragments (AR-1-5692, AR-1-5694, AR-1-5695, AR-1-5707, AR-1-5722, AR-1-5745 to AR-1-5747), three mandible fragments (AR-1-5648, AR-1-5693, AR-1-8567), a angular (AR-1-5738), 48 insolated teeth (AR-1-5647, AR-1-5684 to AR-1-5686, AR-1-5754 to AR-1-5761, AR-1-8519 to AR-1-8552, AR-1-8554, AR-1-8555), two cervical ribs (AR-1-5633, AR-1-8586), 12 vertebrae (AR-1-5606, AR-1-5612, AR-1-5615, AR-1-5616, AR-1-5618, AR-1-5622, AR-1-5749 to AR-1-5752, AR-1-8565, AR-1-8566), 16 vertebra fragments (AR-1-5605, AR-1-5610, AR-1-5623, AR-1-5634, AR-1-5678, AR-1-5679, AR-1-5693, AR-1-5705, AR-1-5708, AR-1-5721, AR-1-8585, AR-1-8587 to AR-1-8590, AR-1-8606), 16 ribs (AR-1-5613, AR-1-5620, AR-1-5624, AR-1-5629, AR-1-5630, AR-1-5636, AR-1-5649, AR-1-5660, AR-1-5704, AR-1-5723 to AR-1-5728, AR-1-8612), 38 rib fragments (AR-1-5621, AR-1-5637, AR-1-5650, AR-1-5677, AR-1-5680, AR-1-5697 to AR-1-5703, AR-1-5748, AR-1-8564, AR-1-8569 to AR-1-8584, AR-1-8613 to AR-1-8615, AR-1-8617, AR-1-8618, AR-1-8621 to AR-1-8623), a coracoid (AR-1-5646), a ilium (AR-1-5652), a ilium fragment (AR-1-8592), ischia (AR-1-5706, AR-1-8607), three metapods (AR-1-8616, AR-1-8619, AR-1-8620), 100 dermal armor plates (AR-1-5607 to AR-1-5609, AR-1-5611, AR-1-5614, AR-1-5617, AR-1-5625 to AR-1-5628, AR-1-5631, AR-1-5632, AR-1-5635, AR-1-5638 to AR-1-5645, AR-1-5653 to AR-1-5659, AR-1-5661 to AR-1-5675, AR-1-5681 to AR-1-5683, AR-1-5687 to AR-1-5690, AR-1-5709 to AR-1-5712, AR-1-5714 to AR-1-5720, AR-1-5730 to AR-1-5737, AR-1-5739 to AR-1-5743, AR-1-8556 to AR-1-8563, AR-1-8591, AR-1-8593 to AR-1-8605, AR-1-8608, AR-1-8610, AR-1-8611) and seven undetermined bones and fragments (AR-1-5619, AR-1-5676, AR-1-5691, AR-1-5729, AR-1-5753, AR-1-8568, AR-1-8609)

*Anteophthalmosuchus escuchae*

**AR-1/37 (holotype)**: partial skeleton deposited at Museo Aragonés de Paleontología/Fundación Conjunto Paleontológico de Teruel-Dinópolis (Teruel, Spain), composed by a disarticulated but associated skull (AR-1-1097, including the mandible), 11 isolated teeth (AR-1-1051, AR-1-1096, AR-1-1106 to AR-1-1109, AR-1-1140, AR-1-1167 to AR-1-1169, 17 vertebrae (AR-1-1187, AR-1-1191, AR-1-1192, AR-1-1198 to AR-1-1205, AR-1-1340, 1342, AR-1-1343, AR-1-1345, AR-1-1347, AR-1-1351, AR-1-1355), 6 ribs (AR-1-1193, AR-1-1194, AR-1-1335, AR-1-1337, AR-1-1339, AR-1-1341), a limb bone (AR-1-1095), 16 dermal armor plates (AR-1-1043, AR-1-1047 to AR-1-1049, AR-1-1098, AR-1-1160, AR-1-1165, AR-1-1195, AR-1-1196, AR-1-1346, AR-1-1348 to AR-1-1350, AR-1-1352 to AR-1-1354

*Anteophthalmosuchus escuchae* (subadult individual)

**AR-1/62**: partial skeleton deposited at Museo Aragonés de Paleontología/Fundación Conjunto Paleontológico de Teruel, componed by a skull (AR-1-3422), mandibles (AR-1-3423; AR-1-4676) 4 vertebrae (AR-1-3429, AR-1-4677, AR-1-4678, AR-1-4679), 9 rib (AR-1-3428, AR-1-4691 to AR-1-4698), a left coracoid (AR-1-4686), a left scapula (AR-1-4687), a right scapula (AR-1-4687), a partial humerus (AR-1-4689) 2 pelvic girdle bones (AR-1-4701, AR-1-4702), ulna (AR-1-4690), a metapod (AR-1-4699), 9 dorsal osteoderms (AR-1-3424, AR-1-3425, AR-1-3427, AR-1-4680 to AR-1-4685), a ventral osteoderm (AR-1-3426).

**Goniopholididae Fossil Record**

*Calsoyasuchus valliceps* from the North American Kayenta Formation (Tykoski et al., 2002) is the oldest goniopholidid on record, dated as Sinemurian-Pliensbachian (196.5-183 Ma). The species *Eutretauranosuchus delfsi* (Mook, 1967; Smith *et al*., 2010; Pritchard *et al*., 2013)*, Amphicotylus lucasii* and *Amphicotylus stovalli* (Mook, 1942; Allen, 2012; Erikson, 2016) come from the Upper Jurassic of the Morrison Formation (155.7-145.5 Ma). The earliest Asian record was assigned to Goniopholididae indet. (Kuzmin et al., 2013) and comes from the Middle Jurassic (Bathonian, 167.7-164.7 Mya) of Siberia*. Sunosuchus* sp. forms part of the Callovian (164.7-161.2 Mya) fossil record of China. This genus diversified in Asia (*Sunosuchus junggarensis* from China, and *Sunosuchus shartegensis* from Siberia (Schellhorn *et al*., 2009; Halliday *et al*., 2013) during the Oxfordian-Tithonian (161.2-145.5 Mya), and a peak of diversification occurred during the Early Cretaceous (*Sunosuchus miaoi* in China, and *Siamosuchus phuphokensis* in Thailand; Buffetaut and Ingavat, 1980). The oldest European Goniopholididae recorded is from the Tithonian of Chassiron in France (Vullo *et al*., 2014) although succinctly described. *Goniopholis baryglyphaeus* from the Kimmeridgian of Guimarota in Portugal is better documented (Schwarz, 2002). The same species has been also reported in the locality of Andrès in Pombal (Malafaia *et al*., 2006). Other Kimmeridgian goniopholidids come from Langenberg/Oken in Germany, and North of France. These were respectively assigned to *Goniopholis simus* and Goniopholididae indet. (Sauvage, 1874, 1882; Buffetaut, 1986 and Salisbury et al., 1999; Karl *et al*., 2006)*.*

The Berriasian (145-139.8 Ma) localities of Cherves-de-Cognac (France) and the Wessex localities (England) have provided the greater diversity of European *Goniopholis*: *Goniopholis simus, Goniopholis kiplingi, Goniopholis crassidens, Nannosuchus gracilidens* and *Hulkepholis willetti* (Pouech *et al*., 2006; Mazin & Pouech, 2008; Mazin *et al*., 2006, 2008; Salisbury & Naish, 2011; Martin *et al*., 2016). Other incomplete or ambiguous in age *Goniopholis sp.* come from the lowermost Cretaceous Rabekke (Berriasian) and Jydegård Formations (Berriasian-Valanginian) on the Baltic island of Bornholm, Denmark, and the Annero Formation of Skåne (Berriasian), southernmost Sweden (Schwarz-Wings *et al*., 2009),

The European Barremian goniopholidids are known by a good number of localities, *Anteophthalmosuchus hooleyi* comes from the locality ‘Tie Pits’ in the Isle of Wight, England (Martin *et al.,* 2016; Ristevski *et al*., 2018) and *Anteophthalmosuchus epikrator* from the outcrop of Bernissart in Belgium and Hanover Point on the Isle of Wight (Ristevski *et al*., 2018). The Spanish Barremian comprises the locality of Galve in the Camarillas Formation (Buscalioni & Sanz, 1987 a, b; Sánchez-Hernández *et al*., 2007), and the localities of Uña and Buenache de la Sierra in the La Huérguina Formation of Cuenca,(Brinkmann, 1989, 1992 and Buscalioni *et al*., 2008), together with the localities of Vallipón and Cantalera in the Artoles Formation in Teruel (Ruiz-Omeñaca & Canudo, 2001); and from the Urbión D Formation of Vadillos-San Román de Cameros (Barremian) in La Rioja (Spain) (Ortega *et al*., 1996; Buscalioni *et al*., 2013).The attributions of goniopholidids coming from these Spanish localities have been quoted in figure 1 in the main text.

The Aptian-Albian taxa from Ariño are the latest record of the European Goniopholididae (Buscalioni *et al*., 2013). The record comprises the sympatric and synchronic taxa *Hulkepholis plotos* and *Anteophthalmosuchus escuchae*. Figure 15 in the text includes skull details of the material assigned *Anteophthalmosuchus escuchae* housed at Museo Aragonés de Paleontología/Fundación Conjunto Paleontológico de Teruel-Dinópolis (Teruel, Spain).

**List of characters used in the phylogenetic analyses**

Character list (494 characters) used for the phylogenetic analysis herein, organized in anatomical according (Ristevski *et al*., 2018) with their own references. Comments on recodified characters have been added to the list.

**Skull geometry and dimensions** (Ch. 1 – 10; 2.02% of characters)

| # | Description |
| --- | --- |
| 1 | **Skull height, in posterior view:**  *Clark (1994, ch. 3 modified); Andrade & Bertini (2008a, ch. 2); Andrade et al. (2011, ch. 1).*  0. skull higher than wide, or subequal  1. skull evidently wider than high |
| 2 | **Skull geometry, relative position of tooth row, quadrate articular facet and occipital condyle:**  0. tooth row and quadrate condyle aligned, both at a lower level than the occipital condyle  1. tooth row at a lower level than the quadrate condyle, which is aligned to the occipital condyle  2. tooth row quadrate and occipital condyle all aligned in the same plane  3. tooth row and occipital condyle aligned, but quadrate condyle at a slightly lower level  4. tooth row and quadrate condyle unaligned and quadrate at a lower level, but both below the occipital condyle  5. tooth row and quadrate condyle unaligned and tooth row at a lower level, but both below the occipital condyle |
| 3 | **Skull geometry, relative position of tooth row and occipital condyle:**  *Wu & Sues (1996, ch. 24 modified); Sereno et al. (2003, ch. 46 modified); Pol (2003, ch. 104 modified); Turner & Buckley (2008, ch. 105 modified); Andrade et al. (2011, ch. 3).*  0. unaligned, tooth row at a lower level than occipital condyle  1. tooth row and occipital condyle aligned in the same plane |
| 4 | **Skull geometry, relative position of quadrate and occipital condyles:**  *Wu & Sues (1996, ch. 24 modified); Sereno et al. (2003, ch. 46 modified); Pol (2003, ch. 104 modified); Turner & Buckley (2008, ch. 105 modified); Andrade et al. (2011, ch. 4).*  0. unaligned, quadrate condyle at a lower level than the occipital condyle  1. quadrate and occipital condyles aligned in the same plane |
| 5 | **Rostrum, length relative to the total skull length:**  *Wu & Sues (1996, ch. 4 modified); Ortega et al. (2000, ch. 3+4 modified); Andrade et al. (2011, ch. 5).*  0. brevirostrine, rostrum length no more than 55% of the total length  1. mesorostrine, rostrum length shorter than 67% of the total length  2. sublongirostrine, rostrum length longer than 66% of the total length, but not longer than 70%  3. longirostrine, rostrum length longer than 70% of the total length |
| 6 | **Rostrum, relation between height and width: (ORDERED)**  *Clark (1994, ch. 3 modified); Andrade et al. (2011, ch. 6).*  0. wider than high, platyrostral  1. height and width subequal  2. higher than wide, oreinrostral |
| 7 | **Rostrum, in dorsal view – amblygnathy (“bullet-shaped”, with the rostrum retaining its width along almost all its length):**  *Young et al. (2016, ch. 3).*  0. no  1. yes |
| 8 | **Rostrum, relation with the skull at maturity, in dorsal view: (ORDERED)**  *Clark (1994, ch. 2); Andrade et al. (2011, ch. 7).*  0. rostrum well defined, broadening abruptly at orbits  1. rostrum poorly defined, smoothly broadening and fitting the skull at orbits  2. rostrum poorly defined, as broad as skull or slightly wider, smoothly fitting the skull at orbits |
| 9 | **Rostrum, relation with the skull at maturity, in lateral view:**  *Andrade et al. (2011, ch. 8).*  0. rostrum smoothly fits the skull, skull roof progressing towards the tip of the snout at about the same level  1. rostrum smoothly decreases in height from skull, at least towards the mid-rostrum  2. rostrum and skull with a poor fit |
| 10 | **Rostrum, dorsal projection:**  *Andrade et al. (2011, ch. 9).*  0. absent, rostrum straight or low  1. present and evident, rostrum bulges dorsally, with nasals assuming an arched profile in lateral view |

**Craniomandibular ornamentation** (Ch. 11 – 21; 2.22% of characters)

| # | Description |
| --- | --- |
| 11 | **Ornamentation, bony surface sculpted with an anastomosed arrangement of wrinkles and ridges, composing a vermiform-dendritic pattern:**  *Ortega et al. (2000, ch. 1 modified); Andrade et al. (2011, ch. 10).*  0. absent  1. present |
| 12 | **Ornamentation, bony surface sculpted with elliptic to subpolygonal pits and grooves, composing a pitted pattern:**  *Ortega et al. (2000, ch. 1 modified); Andrade et al. (2011, ch. 11).*  0. absent  1. present |
| 13 | **Ornamentation, proportion between pits and grooves relative to the ornamented area, at late ontogeny:**  *Andrade et al. (2011, ch. 12).*  0. ornamentation dominated by pits, with grooves almost entirely absent  1. pits and grooves well represented on the skull, with grooves usually present (e.g.,  maxillo-jugal suture, frontal, dentary) late in ontogeny  2. ornamentation dominated by grooves |
| 14 | **Ornamentation, presence of pitted pattern on the postorbital bar, if skull sculpted:**  *Clark (1994, ch. 25 revised); Andrade et al. (2011, ch. 13).*  0. absent  1. present |
| 15 | **Ornamentation, presence of pitted pattern on the jugal:**  *Andrade et al. (2011, ch. 14).*  0. absent  1. present |
| 16 | **Ornamentation, distribution of pitted pattern on the jugal surface:**  *Andrade et al. (2011, ch. 15).*  0. evident ornamentation only occurs at the anterior ramus  1. evident ornamentation occurs on the anterior and posterior rami |
| 17 | **Ornamentation, presence of pitted pattern on the quadratojugal:**  *Pol (1999a, ch. 161); Pol & Apesteguia (2005, ch. 144); Turner & Buckley (2008, ch. 145); Andrade et al. (2011, ch. 16).*  0. absent  1. present, restricted to the distal end |
| 18 | **Ornamentation (mandible), presence of strong pitted pattern on surangular-articular:**  *Andrade et al. (2011, ch. 17).*  0. absent  1. present |
| 19 | **Ornamentation (mandible), presence of strong pitted pattern on angular:**  *Andrade et al. (2011, ch. 18).*  0. absent  1. present |
| 20 | **Sculpturing, palatal surface of maxilla:**  *Ortega et al. (2000, ch. 2); Andrade et al. (2011, ch. 20).*  0. absent, palatal surface smooth.  1. present, palatal surface ornamented with ridges |
| 21 | **Sculpturing, presence on the palatal surface of pterygoid:**  *Clark (1994, ch. 40); Andrade et al. (2011, ch. 21).*  0. absent, surface smooth  1. present |

**Rostral neurovascular foramina** (Ch. 22– 27; 1.21% of characters)

| # | Description |
| --- | --- |
| 22 | **Neurovascular foramina, presence of an expanded network of openings on the dorsal surface of the rostrum and ventral-lateral surfaces of the mandible:**  *Andrade et al. (2011, ch. 22).*  0. absent, neurovascular openings limited to a single line, near the ventral margin of the  rostrum and dorsal margin of dentary  1. present at least at the premaxillae, maxillae and dentaries |
| 23 | **Neurovascular foramina (premaxilla), overall distance to the alveolar margin and teeth:**  *Andrade & Bertini (2008, ch. 17part); Andrade et al. (2011, ch. 23).*  0. ventral-most foramina reach area next to the alveolar margin, close to teeth  1. ventral-most foramina clearly apart from the alveolar margin, distant to the teeth |
| 24 | **Neurovascular foramina (anterior maxilla), overall distance to the alveolar margin and teeth:**  *Andrade & Bertini (2008, ch. 17part); Andrade et al. (2011, ch. 24).*  0. ventral-most foramina reach area next to the alveolar margin, close to teeth  1. ventral-most foramina clearly apart from the alveolar margin, distant to the teeth |
| 25 | **Neurovascular foramina (mid maxilla) forming a strongly arched line at mid-rostrum, at maturity:**  *Andrade et al. (2011, ch. 25).*  *State (1) is putative apomorphy of* Araripesuchus  0. absent, line of foramina follows the overall outline of the margin  1. present, ample area of smooth margin ventral to the arched line of foramina |
| 26 | **Neurovascular foramina (posterior maxilla), distribution on the alveolar margin**  *based on Andrade & Bertini (2008, ch. 17); Andrade et al. (2011, ch. 26).*  0. ventral-most foramina not high on the maxillary margin, either close or next to the alveoli  1. ventral-most foramina high on the maxilla (up to twice the distance from other foramina), very distant to the alveoli |
| 27 | **Neurovascular foramina (dentary), distribution of neurovascular foramina relative to the alveolar margin, on the non-tubular snouted forms: (in external or lateral surface)**  *Andrade et al. (2011, ch. 27).*  0. foramina form a simple straight to ventrally-arched line  1. foramina form a sinusoid line, following the dorsal fluttings, when fluttings are present |

**Cranial rostrum** (Ch. 28– 98; 14.31% of characters)

| # | Description |
| --- | --- |
| 28 | **Foramen at premaxillomaxillary suture in lateral view:**  *Pol (1999, ch. 149); Ortega et al. (2000, ch. 13 revised); Turner & Buckley (2008, ch. 135); Sereno & Larsson (2009 ch. 84 modifed); Andrade et al. (2011, ch. 28).*  0. absent  1. present |
| 29 | **Perinarial crests, presence and morphology:**  *Andrade et al. (2011, ch. 29).*  0. absent, surface even or bearing a perinarial fossa  1. present as well defined and distinct ridges, cornering the lateral to posterior borders of the naris |
| 30 | **Lachrymal crest anterior to orbit, presence (Young & Andrade 2009):**  *Andrade et al. (2011, ch. 30).*  0. absent  1. present |
| 31 | **Naris, orientation in the sagittal plane: (ORDERED)**  *Clark (1994, ch. 6 modifed); Sereno et al. (2003, ch. 2+ch. 7);Andrade et al. (2011, ch. 31).*  0. not dorsalised, either anterior or lateral, but nasal cavity not visible in dorsal view  1. antero-dorsal  2. dorsal |
| 32 | **Naris, shape of narial opening in anterodorsally or dorsally oriented naris (not considering the internarial bar, when present):**  *Andrade et al. (2011, ch. 32).*  0. subcircular, approximately as long as wide  1. heart-shaped  2. keyhole-shaped, subcircular anteriorly, but elongated and subquadratic posteriorly  3. highly elliptic  4. wider than long |
| 33 | **Naris, distance from the anteriormost edge of premaxillae:**  0. narial opening is close to the anterior tip of the snout, regardless of the presence of contact between anterior rami of premaxillae, or the orientation of naris  1. narial opening is distant to the anterior tip of the snout, with anterior rami of premaxillae meeting broadly anterior to naris |
| 34 | **Naris, presence of an anterior narial notch:**  *Andrade et al. (2011, ch. 34).*  0. absent  1. present |
| 35 | **Naris, presence of lateral narial notch:**  *Pol (1999a, ch. 135); Turner & Buckley (2008, ch. 123); Andrade et al. (2011, ch. 35).*  *The notch is fully formed in the hyperadult of* Hulkepholis plotos.  0. absent  1. present |
| 36 | **Naris, composition of dorsal/posterior border:**  *Pol (1999a, ch. 136 modified); Pol (2003, ch. 124); Turner & Buckley (2008, ch. 124 modified); Andrade et al. (2011, ch. 36).*  0. formed mostly by the nasals  1. formed mostly by premaxilla, or nasal excluded from dorsal/posterior border |
| 37 | **Naris, presence and morphology of the internarial bar, in late ontogeny:**  *Clark (1994, ch. 66 modifed); Sereno et al. (2003, ch. 7 modifed);Andrade et al. (2011, ch. 37).*  0. absent, external nares confluent  1. present, not arched dorsally/anterodorsally and with nasal contribution  2. present, evidently arched dorsally and with nasal contribution  3. present, arched dorsally and with no nasal contribution |
| 38 | **Naris, projection of the internarial bar relative to the main body of premaxilla and narial opening, in late ontogeny:**  *Andrade et al. (2011, ch. 38).*  0. does not project anterior to the main body of premaxilla  1. strongly projected anteriorly from narial opening, anterior to main body of premaxilla |
| 39 | **Naris, presence of dorsal projection of anterior rami of premaxillae, and proportional participation in the internarial bar:**  *Clark (1994, ch. 4 revised); Andrade et al. (2011, ch. 39).*  0. premaxillae does not project, or projection is incipient and poorly defined, feebly contributing to internarial bar  1. premaxillae project from the medial contact of anterior rami, composing at least the base of the internarial bar, or an evident bar-like projection if bar is absent |
| 40 | **Perinarial fossa, presence and extent:**  *Sereno et al. (2003, ch. 13 modifed); Turner & Buckley (2008, ch. 226); Sereno & Larsson (2009, ch. 82 modifed);Andrade et al. (2011, ch. 40).*  0. absent or incipient  1. present, small, shallow and mostly limited to the ventral part of the naris  2. present, extremely well-developed and deep, widely extending lateral to the naris |
| 41 | **Postnarial fossa, presence:**  *Andrade et al. (2011, ch. 41).*  0. absent  1. present |
| 42 | **Intranarial fossa, presence at the lateral walls, inside narial cavity, at the vestibulum:**  *Andrade et al. (2011, ch. 42).*  *Interestingly, it could be present in the hyperadult of Hulkepholis plotos, but due to preservation it cannot be ascertained.*  0. absent  1. present |
| 43 | **Antorbital cavity, presence:**  *Clark (1994, ch. 67 modifed);Andrade et al. (2011, ch. 43).*  0. absent  1. present |
| 44 | **Antorbital cavity, presence of internal antorbital fenestra:**  *Clark (1994, ch. 67 modifed);Andrade et al. (2011, ch. 44).*  0. absent  1. present, connecting with internal sinus |
| 45 | **Antorbital cavity, relation between external and internal antorbital fenestrae:**  *Andrade et al. (2011, ch. 45).*  0. external and internal fenestrae subequal or not distinguishable  1. external fenestra larger than internal fenestra, but no more than twice its area  2. external fenestra much larger than internal fenestra, or external fenestra present and internal fenestra closed |
| 46 | **Antorbital cavity, shape:**  Gasparini *et al. (2006, ch. 246);Andrade et al. (2011, ch. 46).*  0. subcircular to subpoligonal  1. strongly elliptic, length at least twice its height, and obliquely orientated at approximately 30 degrees with respect to the longitudinal axis |
| 47 | **Antorbital cavity, size (area) of external antorbital fenestra, relative to the orbit:**  *Clark (1994, ch. 67 modifed);Andrade et al. (2011, ch. 47).*  0. small, less than 50% the orbit area, or antorbital cavity absent  1. large, almost as large as the orbit |
| 48 | **Antorbital cavity, size (length) of internal antorbital fenestra relative to the orbit:**  *Clark (1994, ch. 67 modifed);Andrade et al. (2011, ch. 48).*  0. small, internal fenestra is less than 25% of the length of the orbit, or internal fenestra is absent  1. medium, internal fenestra is approximately 25-50% of the length of the orbit  2. large, internal fenestra is more than 50% of the length of the orbit  3. very large, internal fenestra approximately the same size as the orbit |
| 49 | **Antorbital cavity, nasal participation in the internal fenestra:**  *Ortega et al. (2000, ch. 70 revised);Andrade et al. (2011, ch. 49).*  0. absent, nasals excluded from the internal fenestra by a maxillo-lachrymal contact  1. present, nasals broadly reach the internal fenestra (or reach deep into the fossa, if the fenestra is closed) |
| 50 | **Antorbital cavity, jugal participation in the external fenestra:**  0. absent, jugal excluded from the external fenestra by a maxillary-lachrymal contact  1. present, jugal takes part in the external fenestra |
| 51 | **Antorbital cavity, position relative to the rostrum:**  *Andrade et al. (2011, ch. 51 modified).*  0. closer to the orbit than to the alveolar margin  1. closer to the alveolar margin than to the orbit, or approximately equidistant (but with the cavity still noticeably anterior to the orbit) |
| 52 | **Antorbital cavity, position relative to the orbit:**  *Andrade et al. (2011, ch.52).*  0. close to the orbit, with lachrymal narrow between orbit and antorbital cavity  1. distant to the orbit, with lachrymal wide between orbit and antorbital cavity |
| 53 | **Lachrymal fossa, presence:**  *Andrade et al. (2011, ch. 53).*  *The depression is not as profound in* Eutretauranosuchus *and* Amphicotylus *as in the European goniopholidids.*  0. absent, bony surface completely plain or convex  1. present as a small and shallow depression at the anteriormost corner of the orbit, but with most bony surface plain or convex  2. present and fully developed, with most bony surface anterior to orbit concave |
| 54 | **Rostrum, orientation of external surface of premaxillae and maxillae:**  *Pol (1999, ch. 153 modifed); Turner & Buckley (2008, ch. 139 modifed); Andrade et al. (2011, ch. 54).*  0. premaxillae and maxillae face laterally  1. rostrum with ventral region facing laterally to ventrolaterally, and dorsal region  facing dorsolaterally |
| 55 | **Rostrum, morphology of the external surface of premaxilla and maxilla:**  *based on Pol (1999, ch. 153); Andrade et al. (2011, ch. 55).*  0. rostrum with a continuous surface, either convex or plain  1. rostrum with distinct ventral and dorsal surfaces, plain and separated by a somewhat distinct anteroposterior ridge or edge |
| 56 | **Rostrum, presence of constriction at the premaxillae-maxillae suture, in dorsal view, either forming a notch, a shallow concavity or a narrow slit:**  *based on Clark (1994, ch. 9); Andrade et al. (2011, ch. 56).*  0. absent  1. present |
| 57 | **Rostrum, type of contriction at the premaxilla-maxilla suture:**  *Clark (1994, ch. 9 modifed);Andrade et al. (2011, ch. 57).*  Calsoyasuchus *is not anteroposteriorly wide*.  0. narrow slit  1. wide, poorly-defined concavity, or not constricted at all  2. well-defined notch |
| 58 | **Rostrum, morphology of notch at the premaxillae-maxillae suture:**  *Andrade et al. (2011, ch. 58).*  0. notch absent, or poorly encasing the tooth (< 50% of crown perimetre), usually only part of the lingual surface of the crown  1. notch closely encasing tooth (c. 50%-60%), at least the lingual surface of the crown  2. notch strongly encase tooth in a scabbard-like notch (c. 70%), including most of lingual/labial surfaces of the crown |
| 59 | **Rostrum, presence of a posterodorsal process of premaxilla, at contact with maxilla and nasal:**  *Pol (1999a, ch. 138); Turner & Buckley (2008, ch. 125); Andrade et al. (2011, ch. 59).*  0. absent  1. present, extending posteriorly, wedging between maxilla and nasals |
| 60 | **Rostrum, morphology of contact at premaxillae-maxillae suture, in dorsal/lateral views:**  *based on Gasparini et al. (1991, ch. 3) and description of* Hamadasuchus *in Larsson & Sues (2007); Andrade et al. (2011, ch. 60).*  0. simple, suture not interdigitating  1. complex, with an anteriorly directed process from maxilla fitting the premaxilla |
| 61 | **Rostrum, presence of wedge-like process of maxilla to the premaxilla, at premaxilla-maxilla suture:**  *Gasparini et al. (1991, ch. 3); Turner & Buckley (2008, ch. 213);Andrade et al. (2011, ch. 61).*  0. absent  1. present, wedge-like, anteriorly directed and fitting the premaxilla |
| 62 | **Premaxillae anterior to naris, morphology:**  *Clark (1995, ch. 5 modifed);Andrade et al. (2011, ch. 62).*  0. anterior rami of premaxillae do not meet medially, anterior/ventral to naris, with both premaxillae in contact only through palatine rami  1. anterior rami of premaxillae meet anterior to naris, through a very narrow band, but not projecting vertically  2. anterior rami of premaxillae broadly meet anterior to naris, forming a vertical wall, which may be straight or slightly convex |
| 63 | **Premaxilla, type of contact with maxilla:**  *Clark (1994, ch. 8); Andrade et al. (2011, ch. 63).*  0. premaxilla loosely overlies maxilla on face  1. premaxilla and maxilla suture together along butt joint |
| 64 | **Premaxillae, lateral projection relative to maxillae:**  *Andrade et al. (2011, ch. 64).*  0. absent, premaxillae subequal or narrower than maxillae at anterior to mid rostrum  1. present, premaxillae clearly wider than maxillae at anterior to mid rostrum |
| 65 | **Premaxillae, general profile in dorsal view:**  *Andrade et al. (2011, ch. 65).*  0. round to triangle-shaped, premaxillae smoothly fitting the rostrum  1. lanceolate, premaxillae smoothly fitting the rostrum  2. subquadratic, premaxillae smoothly fitting the rostrum  3. paddle-shaped, expanded laterally  4. axe-shaped, expanded laterally |
| 66 | **RECODED: Premaxillae, presence of a subelliptic naso-oral fossa (=incisive foramen, fossa premaxillaris) at medial contact of ventral rami:**  *Brochu (1999, ch. 124 part);Andrade et al. (2011, ch. 66).*  0. absent, premaxillae fully in contact medially along the palate**,** or very narrow-fossa less than the diameter of the 1^st^ alveolous  1. present as a discrete fossa or foramen, less than half the greatest width of premaxillae  2. large, more than half the greatest width of premaxillae |
| 67 | **Premaxillae, position of the naso-oral fossa in the palatine rami, relative to the alveolar margin:**  *Brochu (1997, 1999, ch. 153 modified); Turner & Buckley (2008, ch. 270 modified); Andrade et al. (2011, ch. 67).*  0. completely situated far from premaxillary tooth-row, at the level of the second or third alveolus, or posterior  1. abuts premaxillary tooth-row  2. projects between first premaxillary teeth |
| 68 | **Premaxillae, contribution of maxillae to the naso-oral fossa at medial contact of ventral rami:**  *Clark (1994, ch. 7 revised); Brochu (1999, ch. 124 part); Andrade et al. (2011, ch. 68).*  0. absent, premaxillae contact medially, posterior to the naso-oral foramen  1. present, maxillae take part at least on the posterior border of the foramen |
| 69 | **Premaxillae, shape of naso-oral fossa:**  *based on Salisbury et al. (1999); Andrade et al. (2011, ch. 69).*  0. oblong, leaf-like  1. teardrop-shaped to slit-like  2. complex, triangular, arrow-headed or fleur-de-liz  3. diamond-shaped  4. elliptic and elongated  5. subcircular |
| 70 | **Premaxillae, shape of naso-oral fenestra:**  *Andrade et al. (2011, ch. 70).*  0. both anterior and posterior ends tapering to an acute end  1. tapering anteriorly only, with round or complex distal end  2. anterior and posterior ends rounded, not tapering |
| 71 | **Premaxilla, presence of foramen in the perinarial depression:**  *Gasparini et al. (2006, ch. 237); Turner & Buckley (2008, ch. 237);Andrade et al. (2011, ch. 71).*  0. absent  1. present |
| 72 | **Nasals, length: (ORDERED)**  *Clark (1994, ch. 13+14);Andrade et al. (2011, ch. 72).*  0. poorly elongated, anteriorly limited by the maxillae only  1. elongated, contacting the maxillae and premaxillae, but not reaching the naris  2. evidently elongated, taking part in the naris |
| 73 | **Nasals, morphology in dorsal view: (ORDERED)**  *Andrade & Bertini (2008a, ch. 21);Andrade et al. (2011, ch. 73).*  0. triangular, lateral margins strongly confluent anteriorly  1. rectangular or subrectangular, lateral margins mostly parallel, or lateral margins poorly confluent anteriorly  2. triangular, lateral margins diverging anteriorly |
| 74 | **Nasals, general structure:**  *Andrade et al. (2011, ch. 74).*  .  0. flattened  1. dome-like |
| 75 | **Nasals, dorsal surface close to the posterior end:**  *Andrade et al. (2011, ch. 75).*  0. continuous, lacking a medial groove or trench  1. slightly concave, forming a shallow and poorly defined depression, anteroposteriorly oriented  2. deeply trenched, with a steep longitudinal depression at midline |
| 76 | **Nasals, morphology of lateral border, posterior to external nares, in dorsal view:**  *Pol (1999, ch. 140 modified); Pol & Apesteguia (2005, ch. 127 modified); Andrade et al. (2011, ch. 76).*  0. laterally concave  1. straight or convex |
| 77 | **Nasals, fusion at maturity:**  *Gasparini et al. (2006, ch. 257); Sereno & Larsson (2009, ch. 10);Andrade et al. (2011, ch. 77).*  0. absent, nasals unfused  1. present, nasals at least partially fused |
| 78 | **Maxilla, participation in the orbit:**  *Andrade (2005, ch. 16); Andrade & Bertini (2008, ch. 15);Andrade et al. (2011, ch. 78).*  0. absent, excluded from the orbit by lachrymal-jugal contact  1. present, maxilla takes part in the orbit |
| 79 | **Maxilla, surface of ventral margin, along the alveoli:**  *Wu & Sues (1996, ch. 29 modified); Martinelli (2003, ch. 24 modified); Turner & Buckley (2008, ch. 107 modified); Andrade et al. (2011, ch. 79).*  0. not distinct from the remaining surface of maxilla, slightly convex  1. maxillae with a distinct ventral surface alongside the alveolar margin, smooth and mostly plane |
| 80 | **Maxilla, projection of ventral margin in lateral view:**  0. ventral maxillary margin is straight  1. ventral maxillary margin festooned, being convex and concave at locations, assuming a sinusoidal profile  2. ventral maxillary margin is overall convex, from contact with premaxilla to contact with jugal |
| 81 | **Maxilla, lateral projection of ventral margin in dorsal view:**  *based on Ortega et al. (2000, ch. 130); Pol & Apesteguia (2005, ch. 171); Andrade et al. (2011, ch. 81).*  0. maxilla does not project laterally at all, and is narrow throughout  1. maxilla expands laterally in locations (coincident with festooning waves, when present), with maxilla sinusoidal in dorsal view  2. maxilla is wide throughout, with no flutting prominent in dorsal view |
| 82 | **Maxilla, number of waves, when festooning present:**  *based on Clark (1994, ch. 79); Ortega et al. (2000, ch. 21);Andrade et al. (2011, ch. 82).*  0. a single clearly identifiable wave is present, at the anterior section of the maxilla, with ventral maxillary margin poorly sinusoidal  1. two major waves clearly identifiable, separated by an evident concave area, with ventral maxillary margin strongly sinusoidal and a corresponding dorsally directed wave on the dorsal edge of the dentary |
| 83 | **83. Maxilla, lateral exposure of occlusal pit for the 11th dentary tooth, at maturity:**  *Andrade et al. (2011, ch. 83).*  0. not exposed laterally, dentitions may overbite or interlock, but lateral wall of occlusal pit is closed  1. laterally open, with occusal surface exposed as a shallow notch |
| 84 | **Maxilla, position of largest alveolus:**  *Ortega et al. (2000, ch. 156 modified);Andrade et al. (2011, ch. 84).*  0. largest alveolus is the third or anterior  1. largest alveolus is the fourth or posterior |
| 85 | **Maxilla, presence of multiple cecal recesses at the surface within narial canal:**  *based on Witmer (1995); Brochu (1997, ch. 148); Andrade et al. (2011, ch. 85).*  0. absent, surface imperforate  1. present |
| 86 | **Maxilla, presence of lateral fossa/fossae next to the alveolar margin:**  *Andrade et al. (2011, ch. 86).*  0. absent, maxillary bony surface convex or flat  1. present |
| 87 | **Maxilla, presence of multiple fossae along most of its lateral face:**  *Wu et al. (2001b);Andrade et al. (2011, ch. 87).*  0. absent  1. present |
| 88 | **Maxilla, presence of a shallow fossa at maxilla-jugal contact:**  *Andrade et al. (2011, ch. 88).*  0. absent  1. present |
| 89 | **Maxilla, presence of a maxillary depression, next to the maxilla-jugal contact:**  *Wu et al. (1997, ch. 127); Sereno et al. (2003, ch. 14); Lauprasert et al. (2007, ch. 97 modified); Andrade et al. (2011, ch. 89).*  0. absent  1. present |
| 90 | **Maxilla, morphology of anterior border of maxillary depressions:**  *Andrade et al. (2011, ch. 90).*  0. shallow, anterior edge of depression usually poorly defined, or maxillary depression is absent  1. deep, anterior border always well-defined relative to dermal surface of maxilla |
| 91 | **Maxilla, extent of contact with nasal:**  *Andrade et al. (2011, ch. 91).*  0. small sutural contact  1. extensive contact |
| 92 | **Maxilla, presence of an evident posteromedial process between lachrymal and nasal:**  *Brochu (1999, ch. 93);Andrade et al. (2011, ch. 92).*  0. absent, maxillary posteromedial process no more than feeble  1. present, maxilla sends an evident posteromedial process within lachrymal |
| 93 | **Maxilla, presence of broad contact with prefrontal:**  *based on Clark (1994, ch. 11) and Ortega et al. (2000, ch. 165 part); Andrade et al. (2011, ch. 93).*  0. maxilla and prefrontal do not contact at all, or with a feeble contact  1. present and evident, maxilla extensively contacts prefrontal |
| 94 | **Lachrymal, relative proportions at maturity:**  *Andrade et al. (2011, ch. 94).*  0. sub-quadratic, or shorter than wide  1. longer than wide |
| 95 | **Lachrymal, proportional length:**  *Andrade et al. (2011, ch. 95).*  0. poorly elongated, AP/ML index closer to 2, or smaller  1. proportionally long, AP/ML index closer to 3, or higher |
| 96 | **Lachrymal, exposure in dorsal aspect:**  *Andrade et al. (2011, ch. 96).*  0. absent, lachrymal is vertically oriented and only exposed in lateral aspect  1. present, either vertical or horizontal |
| 97 | **Lachrymal, presence of a posteroventral process:**  *Andrade et al. (2011, ch. 97).*  0. absent, lachrymal does not progress below orbit, or barely projects through a broad and short wedge  1. present, progress below orbit as an acute process  2. present, progress below orbit as a truncated process |
| 98 | **Lachrymal, extent of contact with nasal:**  *based on Clark (1994, ch. 11+12); as in Ortega et al. (2000, ch. 165 revised); Andrade et al. (2011, ch. 98).*  0. nasal and lachrymal do not contact at all, or with a feeble contact  1. present and evident, lachrymal extensively contacts nasal |

**Skull roof** (Ch. 99– 156; 11.7% of characters)

| # | Description |
| --- | --- |
| 99 | **Prefrontal-lachrymal crest dorsal to orbit, presence and morphology:**  *Brochu (1999, ch. 144 modified);Andrade et al. (2011, ch. 99).*  *See also Poe (1977) and Norell (1988).*  0. absent, or incipient and laterally directed, as a simple edge  1. present and evident, projecting dorsally and short, only slightly progressing anterior to the orbit  2. present and evident, projecting dorsally and long, broadly progressing on the rostrum as very prominent divergent crests |
| 100 | **Transverse rostral crest, presence:**  *Andrade et al. (2011, ch. 100).*  0. absent  1. present |
| 101 | **RECODED: Transverse frontal crest anteromedial to the orbits, presence:**  *Andrade et al. (2011, ch. 101).*  *In* Goniopholis *and* Amphicotylus *the crest is present but in the former, there is no anterior ornamentation in front of the crest, whereas in the later the skull is ornamented.*  0. absent, dorsal skull surface plain or bearing a sagittal crest, or a shallow hump  1-present and steped, crescent-shaped (facing anteriorly), and transecting the skull from orbit |
| 102 | **Frontal sagittal crest, presence:**  *Clark (1994, ch. 22 modifed, part);Andrade et al. (2011, ch. 102).*  0. absent  1. present |
| 103 | **Parietal sagittal crest, presence:**  *Clark (1994, ch. 22 part);Andrade et al. (2011, ch. 103).*  0. absent  1. present |
| 104 | **Crests margining the border of the supratemporal fossae, dorsally projected:**  *Andrade et al. (2011, ch. 104).*  0. absent, or present only in part of the perimeter  1. present around the full perimeter |
| 105 | **Supratemporal fenestra, presence of a shallow fossa at its anteromedial corner:**  *Brochu (1999, ch. 92 revised); Turner & Buckley (2008, ch. 265 revised); Andrade et al. (2011, ch. 105).*  0. absent, anteromedial corner of supratemporal fenestra smooth  1. present |
| 106 | **Supratemporal fenestra, size proportional to the orbit at maturity:**  *Clark (1994, ch. 68 modified); Sereno et al. (2003, ch. 4 modified); Andrade & Bertini (2008, ch. 38 modified); Andrade et al. (2011, ch. 106).*  0. clearly smaller than the orbit  1. fenestra subequal to the orbit  2. supratemporal fenestra larger than orbit |
| 107 | **Supratemporal fossa, size proportional to the orbit at maturity:**  *based on Clark (1994, ch. 68); Brochu (1999, ch. 87); Ortega et al. (2000, ch. 70); Andrade et al. (2011, ch. 107).*  0. clearly smaller than the orbit, or fossa closed by skull table elements  1. fossa subequal to the orbit  2. supratemporal fossa larger than orbit |
| 108 | **Supratemporal fossa, relation with surrounding dermal bones of skull roof:**  *Norell (1988, ch. 9 modified); Brochu (1999, ch. 87 modified); Andrade et al. (2011, ch. 108).*  0. dermal bones of skull roof do not overhang rim at maturity, or slightly overhang rim at part of the external fenestra  1. dermal bones of skull roof consistently overhang rim of supratemporal fenestra around the entire edge, near maturity  2. dermal bones of skull roof overhang rim of supratemporal fenestra near maturity, closing or nearly closing the fenestra during ontogeny |
| 109 | **Supratemporal fossa, presence of a main axis:**  *Andrade & Bertini (2008a, ch. 36);Andrade et al. (2011, ch. 109).*  0. main axis indistinct, or poorly distinct  1. main axis evident and much longer than secondary axis |
| 110 | **Supratemporal fossae, orientation of the main axis at late ontogeny:**  *Andrade & Bertini (2008a, ch. 37);Andrade et al. (2011, ch. 110).*  0. both axis diverge anteriorly  1. both axis parallel  2. both axis converge anteriorly |
| 111 | **Supratemporal fossae, overall shape:**  *Andrade et al. (2011, ch. 111).*  0. elliptic(with lateral and medial edges curved, and the axis twice shorter than the anteroposterior)  1. square-shaped to subrectangular(with the lateral and medial edges almost straight and the axis wider than the anteroposterior for the rectangular)  2. triangle-shaped, axis converging medially (with lateral edge straight and anterior and posterior confluent to a point at the medial border)  3. circular(with the lateral edge straight to slightly curve, but the medial one strongly concave, and both axis subequal in dimension)  4. triangle-shaped, axis converging anteromedially |
| 112 | **Supratemporal fossae, angle between posterolateral process of the frontal and the intertemporal bar:**  *Wilkinson et al. (2008, ch. 26 modified); Young & Andrade (2009, ch. 26 modified); Andrade et al. (2011, ch. 98).*  *See diagrammatic explanation for this character in Wilkinson et al. (2008: p.1311, Fig. 4).*  0. angle of c. 90 degrees, with anteromedial corner of the supratemporal fossae mostly rounded  1. angle of c. 50 degrees, with anteromedial corner of the supratemporal fossae acute, wedge-like |
| 113 | **Supratemporal fossae, shape of medial borders:**  *Andrade et al. (2011, ch. 113).*  0. convex, so the intertemporal bar has a double-concave profile  1. borders straight, parallel  2. straight, either convergent or divergent |
| 114 | **Supratemporal fossa, presence of foramina on the medial parietal wall:**  *Norell (1988, ch. 51), Brochu (1999, ch. 104);Andrade et al. (2011, ch. 114).*  0. absent, wall imperforate  1. present, wall bearing foramina |
| 115 | **Skull roof at orbits, general morphology of periorbital elements (frontal, postorbital, palpebral):**  *based on Hulke (1878) and Hooley (1907); Andrade et al. (2011, ch. 115).*  0. overall level with each other and with the remaining skull table  1. lateral processes of frontal arched laterodorsally, with palpebral and postorbital curved dorsally |
| 116 | **Skull roof, alignment of parietal, frontal and nasals in a steep angle:**  *Andrade et al. (2011, ch. 116).*  0. absent  1. present |
| 117 | **Supratemporal skull roof (posterior to orbit), general proportions:**  *Andrade et al. (2011, ch. 117).*  0. about as long as wide  1. clearly longer than wide (at least 1.5 times) |
| 118 | **Supratemporal skull roof, dorsal surface:**  *Clark (1994, ch. 24);Andrade et al. (2011, ch. 118).*  0. surface complex  1. flat skull table present, formed by flattened and levelled surfaces of frontal, postorbital, squamosal and parietal |
| 119 | **Supratemporal skull roof, dorsal curvature and elongation of squamosal prongs, at maturity:**  *Brochu (1999, ch. 140);Andrade et al. (2011, ch. 119).*  0. with broad curvature and short squamosal prongs  1. with nearly horizontal sides and significant squamosal prongs |
| 120 | **Supratemporal skull roof, morphology of frontoparietal suture:**  *Brochu (1999, ch. 86);Andrade et al. (2011, ch. 120).*  0. concave anteriorly, `V` or `U-shaped`  1. transverse to the skull, either straight or complex  2. concave posteriorly, `V` or `U-shaped` |
| 121 | **Supratemporal skull roof, position of frontoparietal suture at medial line of skull, relative to the orbit:**  *based on Mook (1942) and Clark (1994, ch. 23); Andrade et al. (2011, ch. 121).*  0. frontoparietal suture is evidently posterior to orbits  1. frontoparietal suture is at a very anterior position, almost medial to posteriormost border of the orbits, always as a straight line transecting most of the skull roof, and frontal-postorbital contact is extremely reduced |
| 122 | **Supratemporal skull roof, position of frontoparietal suture at medial line of skull, relative to the postorbital bars:**  *reformulated from Clark (1994, ch. 23 modified); Brochu (1999, ch. 81); Buckley & Brochu (1999, ch. 81); Andrade et al. (2011, ch. 122).*  0. distal margin of frontal is medial to the (dorsal end of) postorbital bars, or slightly anterior  1. distal margin of frontal is posterior to the postorbital bars, but not reaching the mid skull roof  2. distal margin of frontal is posterior to the postorbital bars, frankly reaching into the intertemporal bar and mid skull roof |
| 123 | **Supratemporal skull roof, morphology of the postorbital (=anterolateral) corner of skull roof, in dorsal view:**  *Clark (1994, ch. 29 modified);Andrade et al. (2011, ch. 123).*  0. distinct anterior (or anterolateral) and lateral edges, separated by a round to acute anterolateral corner, or a projecting process  1. anterior and lateral edges, separated by a distinct anterolaterally facing edge  2. anterior to lateral edges continuous, curved, without an anterolaterally projecting edge, corner or process |
| 124 | **Supratemporal skull roof (parietal and squamosals), posterior extension of its posterior margin over the occipital surface:**  *Andrade et al. (2011, ch. 124).*  0. absent or feeble  1. present and evident |
| 125 | **Prefrontal, lateral projection:**  *Gasparini et al. (2006, ch. 247);Andrade et al. (2011, ch. 125).*  0. small to medium, not overhanging the orbit  1. extremely enlarged, overhanging the orbit |
| 126 | **Prefrontals, extension of posterior end visible in dorsal and lateral aspects:**  *Andrade et al. (2011, ch. 126).*  0. short, limited to the anteromedial border of the orbit, with frontal reaching the medial to posteromedial border of the orbit  1. long, composing the anteromedial and medial borders of the orbit, with frontal reaching orbit medially and posteromedially  2. very long, reaching the posteromedial borders of the orbit, with frontal reaching orbit posteromedially only |
| 127 | **Prefrontal pillar, presence of contact between prefrontal descending process and palatine ascending process:**  *Clark (1994, ch. 15 part); Andrade & Bertini (2008, ch. 27);Andrade et al. (2011, ch. 127).*  *Nothwidstanding the prefrontal pillar is obliquely inclined in* Goniopholis *(*Hulkepholis*, and* Anteophtalmosuchus hooleyi*)*  0. absent  1. present |
| 128 | **Prefrontal pillar, structure at contact between prefrontal and palatine processes:**  *Clark (1994, ch. 15 part); Andrade & Bertini (2008, ch. 27);Andrade et al. (2011, ch. 128).*  0. small contact area  1. robust contact suture |
| 129 | **Prefrontal pillar, morphology of descending process of prefrontal:**  *Ortega et al. (2000, ch. 30 part); Turner & Buckley (2008, ch. 182 part); Andrade et al. (2011, ch. 129).*  0. descending process transversely expanded, with the lateromedial lamina well developed and anteroposterior lamina small or absent  1. descending process longitudinally expanded, with the anteroposterior lamina well developed and lateromedial lamina small or absent |
| 130 | **Prefrontal pillar, morphology of ascending process of palatine, when pillar complete:**  *Ortega et al. (2000, ch. 30 part); Turner & Buckley (2008, ch. 182 part); Andrade et al. (2011, ch. 130).*  0. ascending process transversely expanded  1. ascending process columnar, or anteroposteriorly elongated |
| 131 | **Prefrontal pillar, morphology of medial process:**  *Brochu (1999, ch. 136);Andrade et al. (2011, ch. 131).*  0. expanded dorsoventrally  1. expanded anteroposteriorly |
| 132 | **Frontals, fusion of main body at maturity:**  *Clark (1994, ch. 21 modified);Andrade et al. (2011, ch. 132).*  0. not fused, and a medial suture fully persists in late ontogeny, with frontals paired  1. frontals are completely fused into a single element, or at least the main body is completely fused |
| 133 | **Frontal, width relative to the total skull width, at maturity:**  *Andrade et al. (2011, ch. 133).*  0. narrow, approximately one-fifth of skull width, or narrower  1. wide, approximately one-third of skull width, or more |
| 134 | **Frontal, proportional width of main body (between orbits) relative to width of the supratemporal skull roof, at maturity:**  *based on Clark (1994, ch20); Andrade et al. (2011, ch. 134).*  0. narrow, usually 20-30% of the width of the skull roof  1. wide, usually 40-50% of the width of skull roof |
| 135 | **Frontal, general geometry:**  *Brochu (1997, ch. 103 modified);Andrade et al. (2011, ch. 135).*  0. essentially flat, lateral margins flush with skull surface  1. concave, medial borders of the orbit upturned, forming ridged orbital margins |
| 136 | **Frontal, morphology of anterior process:**  *Andrade et al. (2011, ch. 136).*  *The character state (1) does not discriminate between the W wedge-like process of the frontal in Goniopholis and the triangle shaped process in Hulkepholis and Anteophalmosuchus*.  0. wide throughout  1. wide posteriorly and tapering anteriorly, wedge-like to triangle-shaped  2. narrow throughout, as a distinct projection |
| 137 | **Frontal, morphology of anterior-most border of anterior process:**  *Andrade et al. (2011, ch. 137).*  0. truncated  1. wedge-like, either broad or acute |
| 138 | **Frontal, medial surface:**  *Andrade et al. (2011, ch. 138).*  0. frontal is continuous, from anterior margin to posterior end  1. frontal is divided into two plain surfaces, being the anterior at a slightly more ventral level than the posterior |
| 139 | **Frontal, presence of a tuberous intumescent projection on the medial line, between the orbits:**  *based on description by Andrade & Hornung (2011); Andrade et al. (2011, ch. 139).*  0. absent, or very incipient as a humped differentiation of the ornamentation medial or parasagittal  1. present, well defined |
| 140 | **Frontal, extension of anterior margin at late ontogeny:**  *Andrade et al. (2011, ch. 140).*  0. short, does not progress anterior to the orbits  1. long, progress anterior to the orbits |
| 141 | **RECODED: Frontal, participation in the primary medial border of orbit, at dorsal skull roof:**  *based on Salisbury et al. (1999), Schwarz (2002), and Andrade & Hornung (2011); Andrade et al. (2011, ch. 141).*  0. extensive participation in the orbit  1. Participation is very restricted  2. Excluded from the orbit by prefrontal-postorbital contact |
| 142 | **Frontal (anterior process), position relative to tip of prefrontal:**  *Sereno et al. (2001, ch. 27); Turner & Buckley (2008, ch. 238); Andrade et al. (2011, ch. 142).*  0. ends evidently posterior to tip of prefrontals  1. reach or barely surpass the anteriormost tip of prefrontals |
| 143 | **Frontal, presence of wedge-like processes projecting posterolaterally from the distal margin:**  *based on Brochu (1999, ch. 81) and Buckley & Brochu (1999, ch. 81), but with a different*  *conception; Andrade et al. (2011, ch. 143).*  0. absent  1. present, wedging between postorbital and parietal, and frankly projecting towards or inside the supratemporal fossae |
| 144 | **Parietals, fusion in adults:**  *Ortega et al. (2000, ch. 28);Andrade et al. (2011, ch. 144).*  *Note that also frontals may be unfused and eventually both elements are not fused. Frontals are unfused in* Amphycotilus stovalii *and* G. kiplingi*. The trait is not linked to age, since in hyperadult* Hulkepholis plotos *the medial suture of both bones is not fused.*  0. absent, parietals and/or frontal are paired elements  1. present, parietal is a single element |
| 145 | **Parietal, morphology of the medial surface at maturity: (ORDERED)**  *Clark (1994, ch. 33 modified);Andrade et al. (2011, ch. 145).*  0. broad throughout, with a wide sculpted region separating fossae  1. narrow(less than the lateral supratemporal bar), but with a flattened surface separating the fossae, which may be sculpted or not  2. narrow, forming a sagittal crest |
| 146 | **Parietal, dorsal projection of the medial surface, relative to the skull roof:**  *Andrade et al. (2011, ch. 146).*  0. does not project dorsally  1. projects dorsally |
| 147 | **Postparietal (=dermosupraoccipital), presence as a distinct element:**  *Clark (1994; ch. 34 revised); Andrade et al. (2011, ch. 147).*  0. absent or fused with parietal  1. present |
| 148 | **Upper temporal bars, orientation in dorsal view:**  *Ortega et al. (2000, ch. 157 modified);Andrade et al. (2011, ch. 148).*  *Upper (=postorbital-squamosal) temporal bar.*  0. temporal bars mostly parallel, giving the skull roof a rectangular outline in dorsal view  1. temporal bars oblique and anteriorly convergent, giving the skull roof a trapezoidal outline in dorsal view |
| 149 | **Upper temporal bars, outline of lateral margins in dorsal view:**  *Andrade et al. (2011, ch. 149).*  0. margin mostly straight or slightly convex  1. margin strongly sinusoidal: |
| 150 | **Upper temporal bar, position relative to intertemporal bar:**  *Andrade et al. (2011, ch. 150).*  0. upper temporal bar is levelled with intertemporal bar  1. upper temporal bar is ventrally displaced relative to the intertemporal bar, coincident with the horizontal plane, and rotated, with dorsal surface exposed laterally and ventral surface medially  2. upper temporal bar is diagonally displaced relative to the intertemporal bar (posterior end more ventrally displaced than anterior end), but not rotated |
| 151 | **RECODED: Upper temporal bar, relative participation of postorbital(in dorsal aspect and not taking into account any anterior projection of the postorbital nor the squamosal lobe):**  *Ortega et al. (2000, ch. 33 modified);Andrade et al. (2011, ch. 151).*  0*.* small, postorbital represents less than 30% of the bar  1. postorbital between 30-50% of upper temporal bar  2. extensive, postorbital represents more than 50% of the bar |
| 152 | **Postorbital, presence and elongation of a robust anterolateral process projecting from its dorsal surface and postorbital bar: (ORDERED)**  *Clark (1994, ch. 28 modified); Turner & Buckley (2008, ch. 28 modified); Andrade et al. (2011, ch. 98).*  0. absent or poorly developed  1. present and short, but evident  2. present and very long, reaching the lateral corner of the orbit |
| 153 | **Postorbital, relation of anterolateral process and its lamina (when present) to the orbit and anterior jugal ramus:**  *Andrade et al. (2011, ch. 153).*  0. orbit not shielded laterally by a postorbital process and its lamina  1. postorbital process almost reaching or reaching the dorsal edge of the anterior jugal ramus, with process and lamina shielding the posterolateral-lateral sections of the orbit |
| 154 | **Squamosals, presence of horn-like flanges projecting dorsolateraly from the lateral edge:**  *based on Brochu (2007) and Turner & Buckley (2008); Andrade et al. (2011, ch. 154).*  0. absent, lateral surface of squamosal level with frontal or at a lower plane  1. absent, but edges buttressed and evidently enlarged  2. present |
| 155 | **RECODED:Squamosal, presence of an extra lobe, unsculpted, at the posterodorsal corner:**  *Clark (1994, ch. 35); Lauprasert et al. (2007, ch. 32);Andrade et al. (2011, ch. 155).*  0. absent, posterodorsal border of squamosal squared off  1. present, unsculpted discrete lobe demarcated from the rest of the skull table by a sulcus or step projecting posterolateraly  2. present, caudolateral lobe diferentiated of the skull table by a heavy sculptured or bulked, but in continuity. |
| 156 | **Squamosal, orientation of the posterior face at the occipital surface, at maturity:**  *Andrade & Bertini (2008a, ch. 77);Andrade et al. (2011, ch. 156).*  0. mostly vertical, facing posteriorly  1. inclined, facing posterodorsally |

**Orbits and temporal region** (Ch. 157 – 204; 9.68% of characters)

| # | Description |
| --- | --- |
| 157 | **Orbits, orientation in dorsal view (not considering palpebrals): (ORDERED)**  *Andrade et al. (2011, ch. 157).*  0. orbits fully lateral  1. orbits face dorsolaterally  2. orbits with a strong dorsal component |
| 158 | **Orbits, orientation in lateral view:**  *Andrade et al. (2011, ch. 158).*  0. orbits are lateral-dorsal, with a small anterior component, frontal horizontal or poorly inclined  1. orbits have a strong anterior component, frontal steep and facing anterodorsally |
| 159 | **Orbits, presence of sclerotic ring:**  *Andrade et al. (2011, ch. 159).*  0. absent  1. present |
| 160 | **Orbit, composition of anterior (lachrymal) border:**  *Andrade et al. (2011, ch. 160).*  0. lachrymal, but with extensive participation of jugal  1. lachrymal only, neither prefrontal nor jugal reach the lachrymal area  2. lachrymal, but with extensive participation of prefrontal |
| 161 | **Orbit, morphology of medial (primary) border:**  *based on description by Andrade & Hornung (2011); Andrade et al. (2011, ch. 161).*  0. medial and distal borders at a broad angle (>90 degrees), primary orbit is circular to polygonal  1. medial and distal borders at an acute angle (<90 degrees) and forming a posteromedial corner, with primary orbit triangular |
| 162 | **Laterotemporal fenestra, size proportional to orbit in late ontogeny:**  *based on Clark et al. (2004); Andrade et al. (2011, ch. 162).*  0. small to absent, no more than 20% the area of the orbit  1. large, area is usually no less than 50% of the area of the orbit |
| 163 | **Laterotemporal fenestra, proportions:**  *based on Benton & Clark (1988); Andrade et al. (2011, ch. 163).*  0. longer than high or subequal, usually half the height of the orbit  1. higher than long, and usually as high as the orbit |
| 164 | **Laterotemporal fenestra, shape:**  *based on Benton & Clark (1988); Andrade et al. (2011, ch. 164).*  0. elliptic to subpolygonal  1. clearly triangular |
| 165 | **Laterotemporal fenestra, orientation:**  *Ortega et al. (2000, ch. 46);Andrade et al. (2011, ch. 165).*  0. faces laterally  1. faces laterodorsally |
| 166 | **Laterotemporal fenestra, position of jugal-quadratojugal suture relative to the posteroventral corner:**  *Norell (1989, ch. 10 modified); Brochu (1999, ch. 75 modified); Ortega et al. (2000, ch. 39 modified); Andrade et al. (2011, ch. 166).*  0. jugal-quadratojugal suture lies at ventral edge of the fenestra, with quadratojugal taking part in the lower temporal bar  1. jugal-quadratojugal suture lies at posterior angle of the fenestra, with quadratojugal barely reaching the distal end of the lower temporal bar  2. jugal-quadratojugal suture at posterodorsal edge of the fenestra, with quadratojugal excluded from the lower temporal bar |
| 167 | **Laterotemporal fenestra (spina quadratojugalis), presence of a quadratojugal spine in the distal border of the fenestra:**  *Brochu (1999, ch. 114); Ortega et al. (2000, ch. 46);Andrade et al. (2011, ch. 167).*  0. absent  1. present |
| 168 | **Laterotemporal fenestra (spina quadratojugalis), position of spine on the quadratojugal border, when present:**  *Brochu (1999, ch. 114 modified);Andrade et al. (2011, ch. 168).*  0. low. near distal corner of infratemporal fenestra  1. high, between distal and upper corners of infratemporal fenestra |
| 169 | **Laterotemporal fenestra (spina quadratojugalis), orientation of spine, when present:**  *Brochu (1999, ch. 114 modified);Andrade et al. (2011, ch. 169).*  0. anteriorly-anterodorsally oriented  1. dorsally-anterodorsally oriented |
| 170 | **Laterotemporal fenestra (spina quadratojugalis), elongation of quadratojugal spine, when present and fully preserved:**  *Brochu (1999, ch. 114 modified);Andrade et al. (2011, ch. 170).*  0. short  1. long |
| 171 | **Jugal, participation in the ventral border of the orbit:**  *Mueller-Töwe (2006, ch. 139 revised);Andrade et al. (2011, ch. 171).*  0. reduced participation, or excluded from the orbit by a lachrymal-postorbital contact, ventral to the orbit  1. jugal forms large part or all of the ventral margin of the orbit |
| 172 | **Jugal, cross-section of anterior ramus, beneath orbit:**  *based on Clark (1994, ch. 18); Andrade & Bertini (2008a, ch. 47); Andrade et al. (2011, ch. 172).*  0. subcircular to subpolygonal, ramus rod-like  1. elliptic to laminar, lateral surface of ramus evidently flattened |
| 173 | **Jugal, cross-section of posterior ramus, beneath laterotemporal fenestra:**  *Clark (1994, ch. 18 revised); Andrade et al. (2011, ch. 173).*  0. subcircular to subpolygonal, ramus rod-like  1. elliptic, lateral surface of ramus evidently flattened, blade-like |
| 174 | **Jugal, height of anterior ramus relative to the height of posterior ramus:**  *Clark (1994, ch. 17 modified);Andrade et al. (2011, ch. 174).*  0. anterior ramus approximately as broad as posterior ramus  1. anterior ramus approximately twice as broad as posterior ramus |
| 175 | **Jugal (anterior ramus), height below orbit at maturity:**  *Andrade et al. (2011, ch. 175).*  0. low, jugal only forms a narrow band of bone  1. high, broadly separates orbit from ventral plane of cranium |
| 176 | **Jugal, alignment of anterior and posterior processes:**  *Turner & Buckley (2008, ch. 286); Andrade et al. (2011, ch. 176).*  0. inline dorsoventrally  1. anterior and posterior processes at a sharp angle to one another, with both processes sloping ventrally to form a strongly arched jugal |
| 177 | **Jugal, presence of fossa at ventrolateral surface near ectopterygoid contact:**  *Sereno & Larsson (2009, ch. 46);Andrade et al. (2011, ch. 177).*  0. absent, jugal-ectopterygoid contact is inset from lateral surface of jugal, or at ventral surface of jugal  1. present as a smooth but evident depression, below orbit |
| 178 | **Jugal (anterior ramus), projection of ventral margin at maturity:**  *Andrade et al. (2011, ch. 178).*  0. absent, ventral margin of anterior jugal ramus level with the ventral margin of posterior ramus  1. present and poor, jugal gradually flares ventrally at a low angle, and ventral projection is modest  2. present and deep, jugal flares anteriorly at a steep angle, and ventral projection is conspicuous |
| 179 | **Jugal (anterior ramus), presence of a ridge at dorsal edge, and relation with postorbital bar:**  *Ortega et al. (2000, ch. 34 modified); Turner & Buckley (2008, ch. 167 modified); Andrade et al. (2011, ch. 179).*  0. ridge absent, postorbital bar becomes flush with dorsal surface of jugal  1. ridge present, separating postorbital bar from lateral surface of jugal, but neither conspicuous, nor projecting dorsally  2. ridge present and conspicuous, projecting dorsally and separating postorbital bar from lateral surface of jugal |
| 180 | **Jugal (anterior ramus), presence and number of neurovascular foramina:**  *Andrade (2005, ch. 43 modified); Andrade & Bertini (2008a, ch. 43 modified); Andrade et al. (2011, ch. 180).*  0. single enlarged foramen anteriorly directed  1. absent  2. multiple (2-5) small foramina, usually ventrally oriented |
| 181 | **Jugal (anterior ramus), participation in the rostrum:**  *Pol (1999, ch. 134); Turner & Buckley (2008, ch. 122); Andrade et al. (2011, ch. 181).*  0. ramus short, does not progress anterior to the anterior-most border of the orbit, and does not take part in the rostrum  1. at least moderately elongated, clearly surpassing the anterior-most border of the orbit |
| 182 | **Jugal (anterior ramus), length:**  *Andrade et al. (2011, ch. 182).).*  0. short to moderately elongated, but does not reach beneath the antorbital fenestra, poorly contributing to rostrum  1. long, progress below antorbital fenestra and extensively takes part in the rostrum |
| 183 | **Jugal, morphology of anterior end at maturity:**  *Andrade et al. (2011, ch. 183).*  0. rounded or wedging dorsally/medially  1. truncated  2. wedging ventrally/distally |
| 184 | **Jugal fit to maxilla:**  *Andrade et al. (2011, ch. 184).*  0. ventral border of jugal continuous with ventral border of maxilla, with outline in lateral view straight or smoothly curved  1. ventral borders of jugal and maxilla not leveled and with a poor fit, as the maxilla is at a lower level relative to the jugal |
| 185 | **Jugal, presence of a blade-like prong, laterally projecting from mid-body:**  *Novas et al. (2009);Andrade et al. (2011, ch. 185).*  0. absent  1. present |
| 186 | **Palpebrals, presence and number:**  *Clark (1994, ch. 65 modified); Turner & Buckley (2008, ch. 65); Andrade et al. (2011, ch. 186).*  0. absent, or (anterior) palpebral is deeply fused with prefrontal  1. one large (anterior) palpebral present  2. two large palpebrals (anterior and posterior) present |
| 187 | **Palpebrals, overall structure and size:**  *based on (Clark 1994, ch. 65 modified); Andrade et al. (2011, ch. 187).*  0. palpebrals are small and gracile  1. palpebrals are robust and large  2. palpebrals are robust, but small |
| 188 | **Palpebral (anterior), shape:**  *Andrade et al. (2011, ch. 188).*  0. elongated, sickle-like  1. elongated, delta-like  2. rectangular |
| 189 | **Palpebral (anterior), attachment to the skull:**  *Pol & Norell (2004b, ch. 181); Turner & Buckley (2008, ch. 214); Andrade et al. (2011, ch. 189).*  0. reduced contact, palpebral sutured to skull only closer to its anterior edge, or not sutured at all  1. long contact, palpebrals well sutured to the skull by at least anterior and medial edges  2. extensive contact, palpebral fully sutured to the skull by anterior, medial and posterior edges (including lateral end of posterior edge) |
| 190 | **Palpebrals, contact between anterior and posterior elements, when both are present:**  *Andrade et al. (2011, ch. 190).*  0. no sutural contact  1. small, posterior palpebral only contacts the anterior palpebral by the lateral-most end of posterior edge  2. extensive, posterior palpebral contacts the anterior palpebral by most or all the posterior edge |
| 191 | **Postorbital bar, general structure at maturity:**  *Norell (1989, ch. 3); Brochu (1999, ch. 70 revised); Andrade & Bertini (2008a, ch. 53); Andrade et al. (2011, ch. 191).*  0. slender, gracile  1. massive and robust |
| 192 | **Postorbital bar, cross-section:**  *Benton & Clark (1988); Norell & Clark (1990, ch. 3); Clark (1994, ch. 26 revised); Turner & Buckley (2008, ch. 26 revised); Andrade et al. (2011, ch. 192).*  0. subcircular, bar cylindrical  1. elliptical, bar transversely flattened |
| 193 | **Postorbital bar, relation to dermis:**  *based on a modified version of characters in Clark (1994, c. h25) and Ortega et al. (2000, ch. 34), but not co-dependent. Andrade & Bertini (2008a, ch. 49);Andrade et al. (2011, ch. 193).*  0. sub-dermic, distinct, originating mesially from the jugal ramus  1. dermic, gradually narrowing |
| 194 | **Postorbital bar, inclination in lateral view:**  *Andrade & Bertini (2008, ch. 54);Andrade et al. (2011, ch. 194).*  0. vertical  1. inclined, with dorsal end distal to the ventral end |
| 195 | **Postorbital bar, inclination in anterior view:**  *based on Andrade & Bertini (2008, ch. 54); Andrade et al. (2011, ch. 195).*  0. vertical  1. inclined, with dorsal end medial to the ventral end |
| 196 | **Postorbital bar, presence and number of projections:**  *Norell (1989, ch. 2 modified); Brochu (1999, ch. 134 revised); Andrade et al. (2011, ch. 196).*  0. no projection  1. single projection, generally not prominent  2. two prominent projections |
| 197 | **Postorbital bar, contribution from ectopterygoid:**  *Clark (1994, ch. 26 modified); Sereno et al. (2003, ch. 22); Andrade & Bertini (2008a, ch. 51);Andrade et al. (2011, ch. 197).*  0. absent, bar does not receive contribution from ectopterygoid  1. present, bar receives contribution from ectopterygoid |
| 198 | **Postorbital bar, presence of ectopterygoid-postorbital contact:**  *Clark (1994, ch. 26 modified); Pol (2003, ch. 144); Ortega et al. (2000, ch. 36); Andrade & Bertini (2008a, ch. 52);Andrade et al. (2011, ch. 198).*  0. absent  1. present |
| 199 | **Postorbital bar, composition of lateral surface:**  *Gasparini et al. (2006, ch. 244);Andrade et al. (2011, ch. 199).*  0. lateral surface formed by postorbital and jugal  1. lateral surface formed by postorbital only, with jugal only exposed at the medial face of the bar |
| 200 | **Postorbital bar, morphology of postorbital-jugal contact:**  *Clark (1994, ch. 16 modified); Ortega et al. (2000, ch. 37 modified); Andrade et al. (2011, ch. 200).*  0. postorbital anterior to jugal  1. postorbital medial to jugal, or slightly posterior to  2. postorbital lateral to jugal |
| 201 | **Postorbital bar (postorbital), relation with dorsal part of the postorbital:**  *Norell & Clark (1990, ch. 3); Clark (1994, ch. 30); Salisbury et al. (2006, ch. 175); Andrade et al. (2011, ch. 201).*  0. bar broadens dorsally, continuous with dorsal part of postorbital  1. dorsal part of the postorbital bar constricted, with clear limits from the main body of the postorbital |
| 202 | **Postorbital bar (postorbital), presence of a vascular opening at the lateral edge of the bar, close to the dorsal surface of the postorbital:**  *Clark (1994, ch. 27);Andrade et al. (2011, ch. 202).*  0. absent  1. present |
| 203 | **Quadratojugal, morphology of dorsal process at posterodorsal angle of laterotemporal fenestra:**  *Clark (1994, ch. 19 modified); Turner & Buckley (2008, ch. 19); Andrade et al. (2011, ch. 203).*  0. narrow relative to main body, contacting only a small part of postorbital  1. dorsal end expanded as a broad sheet, extensively contacting the postorbital |
| 204 | **Quadratojugal, extension of anterodorsal ramus:**  *Buscalioni et al. (1992, ch. 6); Brochu (1999, ch. 80);Andrade et al. (2011, ch. 204).*  0. quadratojugal reaches dorsal angle of infratemporal fenestra  1. quadratojugal does not extend to dorsal angle of infratemporal fenestra, and quadrate participates in laterotemporal fenestra |

**Palate and perichoanal structures** (Ch. 205 – 265; 12.3% of characters)

| # | Description |
| --- | --- |
| 205 | **Maxillo-palatine fenestrae:**  *Andrade & Bertini (2008a, ch. 85);Andrade et al. (2011, ch. 205).*  0. absent  1. present, subcircular  2. present, anteroposteriorly elongated |
| 206 | **Suborbital fenestrae, presence and size: (ORDERED)**  *Andrade et al. (2011, ch. 206).*  0. absent  1. present, much smaller than orbits  2. present, subequal or larger than orbits |
| 207 | **Suborbital fenestrae, shape of anterior border:**  *Andrade & Bertini (2008, ch. 86);Andrade et al. (2011, ch. 207).*  0. rounded, smooth  1. in sharp angle, forming a notch, fissure-like |
| 208 | **Anterochoanal fossae: (ORDERED)**  *Andrade et al. (2011, ch. 208).*  0. absent, bony surface flat  1. present as shallow fossae  2. present as deep fossae |
| 209 | **Parachoanal fossae, presence:**  *Andrade & Bertini (2008, ch. 103);Andrade et al. (2011, ch. 209).*  0. absent  1. present |
| 210 | **Palate, palatine rami of premaxillae:**  *Andrade et al. (2011, ch. 210).*  0 absent  1. present, in contact at the medial line and forming the anteriormost section of the secondary bony palate |
| 211 | **Palate, presence of contact between ventral rami of the maxillae:**  *Clark (1994, ch. 10);Andrade et al. (2011, ch. 211).*  0. absent, ventral rami are poorly developed and do not meet at medial line, with secondary palate not formed  1. present, ventral rami meet each other at medial line, or meet vomer, and secondary bony palate formed |
| 212 | **Palate, presence of palatal shelves of palatines, and their relation with the narial passage: (ORDERED)**  *Clark (1994, ch. 37 part);Andrade et al. (2011, ch. 212).*  0. palatal shelves of palatine absent, narial passage only bounded dorsally, by the pterygoid  1. narial passage at least partially bounded by palatal shelves of the palatine, laterally, creating the choanal grove  2. narial passage at least mostly bounded by palatal shelves of the palatine, laterally and ventrally, forming the nasopharyngeal duct |
| 213 | **Palate, relation between palatal shelves of the palatine:**  *Clark (1994, ch. 37 part);Andrade et al. (2011, ch. 2013).*  0. palatal shelves of palatine absent or not fully in contact at medial line  1. palatal shelves of palatine fully developed, bounding the narial passage ventrally an in contact at medial line, creating fully formed nasopharyngeal duct, with secondary palate complete |
| 214 | **Palate, participation of maxilla in the suborbital fenestra:**  *Novas et al. (2009, ch. 231);Andrade et al. (2011, ch. 214).*  0. takes part in the suborbital fenestra  1. fully excluded from the suborbital fenestra by a palatine-ectopterygoid contact, anterior to suborbital fenestra |
| 215 | **Palate, composition of anteromedial border of suborbital fenestrae:**  *Andrade & Bertini (2008a, ch. 88 part);Andrade et al. (2011, ch. 215).*  0. entirely composed of palatines, which expand laterally and reach the anteriormost border of the fenestra  1. palatal ramus of maxilla takes part in the anteromedial border of the fenestra |
| 216 | **Palate, presence of maxillary process to palatine, next to the anterior border of antorbital fenestra:**  *Andrade & Bertini (2008a, ch. 88 part);Andrade et al. (2011, ch. 216).*  0. absent  1. present, palatines composing a three-radiated blade anteriorly |
| 217 | **Palate, direction of the sutural contact of premaxilla-maxilla, at the palate:**  *Ortega et al. (2000, ch. 9 modified);Andrade et al. (2011, ch. 217).*  0. curved posteriorly, premaxillary palate projects over maxillary palate  1. straight, poorly arched or complex  2. curved anteriorly, maxillary palate projects over premaxillary palate |
| 218 | **Palate, presence of maxilla-pterygoid contact medial to suborbital fenestra, in ventral view:**  *Sereno & Larsson (2009);Andrade et al. (2011, ch. 218).*  0. absent, palatines take part in the suborbital fenestra  1. present, excluding palatines from suborbital fenestra |
| 219 | **Palate, relative position of the distalmost suture of palatine, at the border suborbital fenestra:**  *Brochu (1999, ch. 85); Salisbury et al. (2006, ch. 85);Andrade et al. (2011, ch. 219).*  0. suture is at distal end of suborbital fenestra, or lateral to it, but very close  1. suture is located medial to posterior angle of suborbital fenestra, and far from it |
| 220 | **220. Palate canals, presence:**  *Andrade et al. (2011, ch. 220).*  0. absent  1. present |
| 221 | **RECODED: Vomer, ventral exposure on palate:**  *Norell (1988, ch. 22); Brochu (1999, ch. 125); Ortega et al. (2000, ch. 59);Andrade et al. (2011, ch. 221).*  *It has been included the new state, because in Goniopholidids the exposition of the vomers is between the maxilla and palatines are topographically congruente with the position of the primary choana. This trait corresponds to character 271 of Pritchard et al (2013) ventral surface of the choanal septum with bilateral vomeral septal laminae.*  0. not exposed on palate, hidden by palatal branch of maxillae  1. exposed on palate between premaxillae and maxillae  2. exposed on palate between maxilla and palatine |
| 222 | **Palatines, progression of the palatine process through the palate:**  *Andrade et al. (2011, ch. 222).*  0. short, anterior border medial to the anteriormost margin of the suborbital fenestrae, or barely anterior to them  1. long, anterior border of palatines clearly surpassing the anteriormost border of the suborbital fenestrae |
| 223 | **Palatines, proportional length of anterior process, projecting at maxillary palate(taken from their border at the suborbital fenestra):**  *Andrade et al. (2011, ch. 223).*  0. anterior process of palatines short, with length subequal to width  1. evidently longer than wide |
| 224 | **Palatines, overall morphology of palatine process:**  *Andrade et al. (2011, ch. 224).*  0. wide, fan-like  1. wide posteriorly and tapering anteriorly, wedging between palatine rami of maxilla  2. lateral margins parallel or flaring anteriorly |
| 225 | **Palatines, morphology of anterior face of palatine process near medial line:**  *Andrade et al. (2011, ch. 225).*  0. rounded or pointed anteriorly, either U-shaped or V-shaped  1. truncated, maxillo-palatine suture transversally oriented relative to the skull  2. invaginated by at least a broad and short maxillary wedge, maxillo-palatine suture M-shaped |
| 226 | **Palatines, presence of a long medial process of the maxilla, posteriorly directed and dividing the anterior face of palatine process:**  *Andrade et al. (2011, ch. 226).*  0. absent, anterior face of palatine process U-shaped, V-shaped or broad M-shaped  1. present, anterior face of palatine process clearly bifid, narrow M-shaped |
| 227 | **Palatines, heart-shaped anterior face of palatine process:**  *Brochu (1999, ch. 108 modified);Andrade et al. (2011, ch. 227).*  0. absent, maxillo-palatine suture U-shaped, V-shaped, or M-shaped  1. present, anterior palatine process invaginated close to the medial line by maxilla, with maxillo-palatine suture assuming a heart-shaped profile |
| 228 | **Palatine bar, presence:**  *Martinelli (2003, ch. 36 modified); Turner & Buckley (2008, ch. 232 modified); Andrade et al. (2011, ch. 228).*  0. absent  1. present, gracile  2. present as distinctively robust bars |
| 229 | **Nasopharyngeal duct, width at its narrowest section relative to the skull width(taken between quadrates):**  *Andrade et al. (2011, ch. 229).*  0. narrow in proportion to skull width, no more than 25%  1. wide in proportion to skull width, no less than 30% |
| 230 | **Nasopharyngeal duct, presence of a deep sulcation on the ventral surface, where the medial contact of palatine occurs:**  *Andrade et al. (2011, ch. 230).*  0. absent  1. present |
| 231 | **Nasopharyngeal duct, cross-section:**  *Andrade et al. (2011, ch. 231).*  0. width is subequal to height, or greater  1. evidently higher than wide |
| 232 | **Anterochoanal sagittal crest, presence:**  *Andrade et al. (2011, ch. 232).*  0. absent, bony surface is smooth to concave  1. present, pterygoid surface at medial line projects ventrally anterior to the internal naris, forming a discrete and short acute crest  2. present, bony surface of palatines projects ventrally at medial line, anterior to the internal naris, forming a discrete and short acute crest |
| 233 | **RECODED: Choanae, size (area) relative to skull:**  *Clark (1994, ch. 42 modified);Andrade et al. (2011, ch. 233).*  *To include the state of an ample coanal area shown in thegoniopholidids with an open palatal fenestra from the primary coana to the rear border of the pterygoid.*  0. very small  1.wide  2. ample in continuity with the sagittal palatal fenestra and primary choana |
| 234 | **Choanae and perichoanal fossa, width relative to the width of the nasopharyngeal duct:**  *based on Clark (1994, ch. 44), Sereno et al. (2003, ch. 31) and Andrade & Bertini (2008a, ch. 101); Andrade et al. (2011, ch. 234).*  0. narrow, width evidently smaller than the narrowest section of the nasopharyngeal duct  1. subequal, wider than the narrowest section of the nasopharyngeal duct, but not wider than the widest section  2. proportionally wide, clearly wider than the widest section of the nasopharyngeal duct |
| 235 | **Choanae, position of anterior border of the internal naris relative to nasopharyngeal duct and suborbital fenestra: (ORDERED)**  *Clark (1994, ch. 44 modified, part); Pol & Norell (2004ab, ch. 44 modified, part); Andrade et al. (2011, ch. 235).*  0. anterior border situated anterior to the suborbital fenestra  1. anterior border situated at mid-nasopharyngeal duct  2. anterior border close to the posterior end of the nasopharyngeal duct, or posterior to it |
| 236 | **Choanae, position of anterior border of the internal naris relative to pterygoid surface at medial line:**  *Clark (1994, ch. 44 modified, part); Pol & Norell (2004ab, ch. 44 modifed, part); Andrade et al. (2011, ch. 236).*  0. close to anterior end of pterigoyd, or anterior to it  1. at mid/posterior pterygoid surface |
| 237 | **Choanae, position of posterior border relative to pterygoid ventral surface:**  *based on Clark (1994, ch. 44) and Pol & Norell (2004ab, ch. 44); Andrade et al. (2011, ch. 237).*  0. posterior border situated near the anterior edge of the pterygoids, or anterior to pterygoids  1. posterior border situated approximately at mid-pterygoid  2. posterior border at posterior pterygoid surface |
| 238 | **Choanae, shape of anterior border in palatal view:**  *Andrade et al. (2011, ch. 238).*  0. rounded, straight, or invaginated  1. choanal opening wedges between bony lamina as an acute V-shape, internal nares assuming a lanceolate profile |
| 239 | **Choanae, shape in palatal view:**  *Andrade (2005, ch. 87 modified); Andrade & Bertini (2008, ch. 91 modified);Andrade et al. (2011, ch. 239).*  0. subcircular, elliptic or lanceolated  1. triangle-like  2. rectangular  3. V-shaped or reversed triangle  4. butterfly-shaped |
| 240 | **Choanae, general morphology:**  *Andrade et al. (2011, ch. 240).*  0. choanae wider than long  1. length and width subequal  2. choanae longer than wide |
| 241 | **Choanae, orientation:**  *Andrade & Bertini (2008a, ch. 93);Andrade et al. (2011, ch. 241).*  0. ventrally oriented  1. posteroventrally oriented |
| 242 | **Choanae, participation of pterygoid in the choanal border:**  *Clark (1994, ch. 43 modified); Brochu (1999, ch. 71 modified); Jouve et al. (2005, ch. 4 modified); Turner & Buckley (2008, ch. 43 modified); Andrade et al. (2011, ch. 242).*  0. pterygoid only bounds the posterior border of the choanae  1. pterygoid forms at least the posterior and lateral choanal borders  2. anterolateral rami of pterygoid embrace most of the choanae, but do not meet medially, at the anterior choanal border (either by the presence of palatine or ventral exposure and expansion of interchoanal septum)  3. anterolateral rami of pterygoid completely embrace the choanae, meeting medially at its anterior border (eusuchian choanae) |
| 243 | **Choanae, presence of extensive contact between converging perichoanal pterygoid laminae, anterior to choanae:**  *Andrade et al. (2011, ch. 243).*  0. absent, anterior pterygoid laminae narrow anterior to choanae, or not in contact at all  1. present, anterior pterygoid laminae widely meet anterior to choanae |
| 244 | **Interchoanal septum, exposure at internal naris:**  *Clark (1994, ch. 69 modified); Brochu (1999, ch. 152 modified); Ortega et al. (2000, ch. 137 revised); Andrade et al. (2011, ch. 244).*  0. nasopharyngeal septum absent or receeded, not dividing the internal narial opening  1. present, nasopharyngeal septum of pterygoid fully dividing the choanal opening, or evidently projecting from ventral surface of pterygoid, if choanal groove is not enclosed by palatines |
| 245 | **Interchoanal (nasopharyngeal) septum, exposure at the ventral surface of the nasopharyngeal duct, anterior to the choanae:**  *based on Clark (1994, ch. 43) and Turner & Buckley (2008, ch. 43 modified); Andrade et al. (2011, ch. 245).*  0. absent, or nasopharyngeal septum receded and not exposed ventrally at all  1. present, interchoanal septum projects anteriorly between palatines, and expands ventrally, creating a wide surface at the anterior choanal border |
| 246 | **Interchoanal septum, presence of an acute groove on its ventral surface:**  *Turner (2004, ch. 126); Turner & Buckley (2008, ch. 271); Andrade et al. (2011, ch. 246).*  0. absent, ventral surface smooth to slightly depressed  1. present and evident, well-marked |
| 247 | **RECODED: Interchoanal septum, shape:**  *Pol & Apesteguia (2005, ch. 186 modified); Turner & Buckley (2008, ch. 191 modified); Andrade et al. (2011, ch. 247).*  0. narrow vertical bony sheet, and rectangular in cross section  1. narrow vertical bony sheet, expanded ventrally, T-shaped in cross-section  2. narrow vertical bony sheet, inflated ventrally and forming a robust bar, semi-circular in cross-section |
| 248 | **Interchoanal septum, morphology of ventralmost surface, as septum approaches choanal opening:**  *Pol & Apesteguia (2005, ch. 220 modified); Turner & Buckley (2008, ch. 225); Andrade et al. (2011, ch. 248).*  0. parallel sided  1. tapers anteriorly  2. expanding both anteriorly and posteriorly, hourglass-shaped  3. tapers both anteriorly and posteriorly |
| 249 | **Perichoanal ridge, presence at anterolateral edge of internal naris, late in ontogeny:**  *based on Brochu (1999, ch. 73 modified); Andrade et al. (2011, ch. 249).*  0. absent, all anterolateral edge of choanae flush with remaining bony surface  1. present as a well-defined wall, bounding at least the anterolateral border of the internal naris |
| 250 | **Perichoanal ridge, presence at posterolateral edge of choana, late in ontogeny:**  *Andrade et al. (2011, ch. 250).*  0. absent, all posterolateral edge of choanae flush with remaining bony surface  1. present as a well-defined wall, bounding at least the posterolateral border of the internal naris |
| 251 | **Perichoanal ridge, presence of a continuous wall around the internal naris, early in ontogeny:**  *Brochu (1999, ch. 73 modified);Andrade et al. (2011, ch. 251).*  0. absent or incomplete, at least part of the choanal margin flush with bony surface  1. present, continuous |
| 252 | **Ectopterygoid, relation with maxilla and tooth-row:**  *Brochu (1997, ch. 91 modified); Turner & Buckley (2008, ch. 264 modified); Andrade et al. (2011, ch. 252).*  0. ectopterygoid abuts maxillary toothrow at medial wall of distal alveoli  1. ectopterygoid broadly separated from last teeth in the toothrow by palatal ramus of maxilla, or barely contacting maxilla |
| 253 | **Ectopterygoid, presence of broad contact with palatine ramus of maxilla:**  *based on Brochu (1997, ch. 91 modified); Andrade et al. (2011, ch. 253).*  0. absent, ectopterygoid does not contact maxilla, or barely contacts its caudal end, medial to jugal  1. present |
| 254 | **Ectopterygoid, morphology of medial process:**  *Ortega et al. (2000, ch. 146); Turner & Buckley (2008, ch. 180);Andrade et al. (2011, ch. 254).*  0. single  1. forked |
| 255 | **Ectopterygoid, development of the medial ramus:**  *Andrade (2005, ch. 93); Zaher et al. (2006, ch. 196 modified); Andrade & Bertini (2008a, ch. 98);Andrade et al. (2011, ch. 255).*  0. small, do not take part in the internal naris  1. well-developed, taking part in the internal naris |
| 256 | **Ectopterygoid, morphology of the distal ramus:**  *based on description by Pol & Apesteguia (2005: p. 8), where the subcylindrical profile of*  *the distal ramus (1) was noted in* Araripesuchus buitreraensis*; Andrade et al. (2011, ch. 256).*  0. laminar, extending as a flattened sheet over the pterygoid wing  1. robust, extending as a rod over most of the pterygoid wing, with subcircular cross-section through most of its length |
| 257 | **Ectopterygoid, length of posterior ramus, at maturity:**  *Norell (1988, ch. 32 modified); Brochu (1999, ch. 149); Turner & Buckley (2008, ch. 269); Andrade et al. (2011, ch. 257).*  0. reaches posterior tip of lateral pterygoid flange  1. pinched off anterior to posterior tip of lateral pterygoid flange |
| 258 | **Pterygoids, fusion posterior to choanae:**  *Clark (1994, ch. 41);Andrade et al. (2011, ch. 258).*  0. not fused  1. fused |
| 259 | **Pterygoid, participation in the suborbital fenestra:**  *Martinelli (2003, ch. 35 modified); Andrade & Bertini (2008c, ch. 89 modified);Andrade et al. (2011, ch. 259).*  0. pterygoid takes part in the suborbital fenestra  1. pterygoid excluded from the suborbital fenestra by a palatine-ectopterygoid contact |
| 260 | **Pterygoid, presence of depression on primary pterygoidean palate posterior to choanae:**  *Ortega et al. (2000, ch. 139 revised); Turner & Buckley (2008, ch. 42); Andrade et al. (2011, ch. 260).*  0. absent or moderate in size, being narrower than palatine bar  1. wider than palatine bar |
| 261 | **Pterygoid ventral rami (wings), size: (ORDERED)**  *Andrade & Bertini (2008a, ch. 94 modified);Andrade et al. (2011, ch. 261).*  0. very small or vestigial  1. well-developed  2. extremelly well-developed |
| 262 | **Pterygoid ventral rami (wings), ventral surface at distal end:**  *based on the discussions and descriptions by Ortega (2000, 2004), Sereno et al. (2003), Peng & Shu (2005), and Turner & Buckley (2008); Andrade et al. (2011, ch. 262).*  0. plain surface  1. evident transverse ridge on ventral surface, forming a vertically oriented postchoanal wall, or a buttressed bar |
| 263 | **Pterygoid ventral rami (wings), structure:**  *Andrade & Bertini (2008c, ch. 96);Andrade et al. (2011, ch. 263).*  0. laminar  1. robust |
| 264 | **Pterygoid ventral rami (wings), orientation in lateral view:**  *Andrade & Bertini (2008a, ch. 95);Andrade et al. (2011, ch. 264).*  0. poorly to mildly inclined, no more than 45 degrees  1. strongly verticalized, 50 degrees or more relative to the horizontal plane |
| 265 | **Pterygoid ventral rami (wings), extension of posterior border of ventral wings, in ventral view:**  *Andrade et al. (2011, ch. 265).*  0. relatively anterior, not covering the anteromedial end of quadrates  1. relatively posterior, covering the anteromedial end of quadrate |

**Occipital** (Ch. 266 – 280; 3.02% of characters)

| # | Description |
| --- | --- |
| 266 | **Supraoccipital, presence and development of tuberous prominences:**  *Clark (1994, ch. 64); Jouve et al. (2005, ch.3);Andrade et al. (2011, ch. 266).*  0. absent  1. present, modest but evident  2. present and extremely developed |
| 267 | **Supraoccipital, morphology of posterior wall:**  *Andrade et al. (2011, ch. 267).*  0. essentially flat, or projecting distally  1. strongly concave |
| 268 | **Exoccipitals, overall morphology:**  *Norell (1988, ch. 20 modified); Clark (1994, ch. 57 modified); Brochu (1999, ch. 151); Andrade et al. (2011, ch. 268).*  0. terminate dorsal to basioccipital tubera  1. send robust process ventrally and participate in basioccipital tubera  2. send slender process ventrally to basioccipital tubera |
| 269 | **Exoccipitals, presence of a large ventrolateral part ventral to paroccipital process:**  *Clark (1994, ch. 60);Andrade et al. (2011, ch. 269).*  0. absent  1. present |
| 270 | **Exoccipitals, presence of medial contact between both elements:**  *Clark (1994, ch. 62); Ortega et al. (2000, ch. 63);Andrade et al. (2011, ch. 270).*  0. do not meet in midline  1. meet on the midline, dorsal to the basioccipital, excluding the supraoccipital from the foramen magnum |
| 271 | **Exoccipitals, presence of a pronounced transverse ridge dorsal to foramen magnum:**  *based on Peng & Shu (2005);Andrade et al. (2011, ch. 271).*  0. absent or incipient  1. present |
| 272 | **Exoccipitals, participation in the occipital condyle:**  *based on Peng & Shu (2005), as in Jouve et al. (2005, ch. 5);Andrade et al. (2011, ch. 272).*  0. absent or incipient, neither reaching the articular surface nor meeting medially  1. present and evident, reaching the articular surface and meeting medially |
| 273 | **Exoccipital, presence of descending flange ventral to subcapsular process:**  *Clark (1994, ch. 58);Andrade et al. (2011, ch. 273).*  0. absent  1. present, laterally concave |
| 274 | **Exoccipital, extent of contact with the quadrate:**  *Clark (1994, ch. 48 modifed + 51);Andrade et al. (2011, ch. 274).*  0. absent or narrow  1. broad contact present, stabilising the quadrate |
| 275 | **Exoccipital, presence of ventrolateral contact with the ventromedial part of quadrate:**  *Clark (1994, ch. 51 modified);Andrade et al. (2011, ch. 275).*  0. absent, quadrate does not contact exoccipital  1. present, exoccipital and quadrate enclosing carotid artery and forming passage for cranial nerves IX-XI |
| 276 | **Exoccipital, presence of individualised passage (metopic foramen) for cranial nerve IX:**  *Clark (1994, ch. 59); Ortega et al. (2000, ch. 64);Andrade et al. (2011, ch. 276).*  0. absent, cranial nerves IX-XI pass through a common large foramen  1. metopic foramen present, medial to cranial passage for nerves X-XI |
| 277 | **Exoccipitals, presence of an evident boss on lateral edge of paroccipital process:**  *Brochu (1999, ch. 141 revised, part); Turner & Buckley (2008, ch. 268 revised, part); Sereno &*  *Larsson (2009, ch. 166 part);Andrade et al. (2011, ch. 277).*  0. absent, exoccipital with small or no boss on paroccipital process  1. present, paroccipital process with a proportionally robust, thickened lateral/ventrolateral edge |
| 278 | **Exoccipitals, elongation of lateral end (=paraocciptal process) relative to the skull roof:**  *Brochu (1999, ch. 141 revised, part); Turner & Buckley (2008, ch. 268 revised, part); Sereno & Larsson (2009, ch. 166 part); Andrade et al. (2011, ch. 278).*  0. relatively short, does not progress lateral to the skull roof  1. relatively long, clearly progress lateral to the skull roof |
| 279 | **Exoccipital, projection of the lower margin of paraoccipital process:**  *Andrade et al. (2011, ch. 279).*  0. absent or feeble, ventral border usually level with foramen magnum  1. present and evident, lower margin reaching ventrally at least as far as the same level as the occipital condyle |
| 280 | **Exoccipital, morphology of ventral border of paraoccipital process:**  *Andrade et al. (2011, ch. 280).*  0. either projects as a wide blade, or projection is feeble/absent  1. projects ventrally as a narrow bar, rod-like |

**Braincase, basicranium and suspensorium** (Ch. 281 – 309; 5.44% of characters)

| # | Description |
| --- | --- |
| 281 | **Crista interfenestralis between fenestrae pseudorotunda and ovalis, orientation:**  *Clark (1994, ch. 61);Andrade et al. (2011, ch. 281).*  0. nearly vertical  1. horizontal |
| 282 | **Supraoccipital, internal presence of the cavity for the intertympantic diverticulum of the pharyngotympanic sinus system (= the “mastoid antrum”):**  *Clark (1994, ch. 63 modified);Andrade et al. (2011, ch. 282 modified).*  0. absent (in Thalattosuchia this diverticulum is lost)  1. present |
| 283 | **Lateral carotid foramen, position relative to basisphenoid (lateral exposure), at maturity:**  *Brochu (1999, ch. 128);Andrade et al. (2011, ch. 283).*  0. opens lateral to basisphenoid lateral exposure  1. opens dorsal to basisphenoid lateral exposure |
| 284 | **Anterior foramen for palatine ramus of cranial nerve VII, position relative to basisphenoid rostrum** *Brochu (1999, ch. 164); Andrade et al. (2011, ch. 284).*  0. ventrolateral to basisphenoid rostrum  1. ventral to basisphenoid rostrum |
| 285 | **Laterosphenoid, orientation of capitate process:**  *Brochu (1999, ch. 130); Salisbury et al. (2006); Andrade et al. (2011, ch. 285).*  0. capitate process oriented laterally towards midline  1. capitate process oriented anteroposteriorly towards midline |
| 286 | **Basisphenoid, ventral exposure in adults and young individuals, but not immature or hatchlings: (ORDERED)**  *Clark (1994, ch. 55 revised+ 56 revised); Ortega et al. (2000, ch. 68 modified); Andrade et al. (2011, ch. 286).*  1. well-exposed, although basisphenoid surface clearly smaller than basioccipital surface  2. ample surface exposed ventrally, basisphenoid at least as long as the basioccipital, or longer |
| 287 | **Basioccipital, cross-section of occipital condyle:**  *Andrade et al. (2011, ch. 287).*  *State (1) is putative apomorphy of Dyrosauridae*  0. subcircular, condyle not compressed  1. strongly elliptic, condyle dorsoventrally flattened |
| 288 | **RECODED: Basioccipital, presence of basal tubera:**  *Clark (1994, ch. 57); Lauprasert et al. (2007, ch. 46);Andrade et al. (2011, ch. 288).*  0. absent  1. tubera present, large and pendulous  2. widen and with lateral edges turned posteriorly |
| 289 | **Basipterygoid process, development:**  *Clark (1994, ch. 54 revised); Andrade et al. (2011, ch. 289).*  0. small or absent  1. prominent, forming a movable joint with pterygoid, and with basisphenoid joint suturally closed |
| 290 | **Medial pharyngeal and pharyngotympantc tubes (= “Eustachian tubes”), relation to basioccipital and basisphenoid:**  *Clark (1994, ch. 52);Andrade et al. (2011, ch. 290).*  0. not enclosed between basioccipital and basisphenoid  1. entirely enclosed between the basioccipital and basisphenoid |
| 291 | **Quadrate, orientation of main body in lateral view: (ORDERED)**  *Ortega et al. (2000, ch. 44 modified);Andrade et al. (2011, ch. 291).*  0. poorly inclined, subvertical  1. slightly inclined posteriorly, approximately 45 degrees  2. strongly inclined, with quadrate almost horizontal |
| 292 | **Quadrate, orientation in dorsal view: (ORDERED)**  *Andrade et al. (2011, ch. 292).*  0. does not project laterally to the skull, with the lateral surface of the quadrate covered by the squamosal  1. slightly projected laterally to the skull, with most of the laterodistal end of the quadrate covered by the squamosals  2. strongly projected laterally to the skull, exposing most of the distal end of the quadrate |
| 293 | **Quadrate, orientation of distal end and condylar head:**  *Pol (1999, ch. 166 modified); Ortega et al. (2000, ch. 44 modified); Turner & Buckley (2008, ch. 149 modified); Andrade et al. (2011, ch. 277).*  0. directed posteroventrally  1. directed mostly ventrally, or anteroventrally |
| 294 | **Quadrate, presence of preotic siphonial foramen on medial surface, close to tympanum:**  *based on Clark (1994, ch. 45 part); Andrade et al. (2011, ch. 294).*  0. absent  1. present |
| 295 | **Quadrate, presence of fenestrae on the dorsolateral-posteromedial surfaces:**  *Clark (1994, ch. 45 part); Ortega et al. (2000, ch. 51 revised); Sereno et al. (2003, ch. 35); Andrade et al. (2011, ch. 295).*  0. fenestrae absent or limited to one opening (preotic siphonial foramen)  1. two or more fenestrae additional to siphonial foramen (if siphonial foramen present) |
| 296 | **Quadrate, external structure:**  *based on description of* Notosuchus *by Bonaparte (1991), but see also Andrade & Bertini (2008a); Andrade et al. (2011, ch. 296).*  0. non-pneumatic  1. highly pneumatic |
| 297 | **Quadrate, morphology of posterior edge:**  *Clark (1994, ch. 46);Andrade et al. (2011, ch. 297).*  0. broad medial to tympanum, gently concave  1. posterior edge narrow dorsal to exoccipital contact, strongly convex |
| 298 | **Quadrate, articulation of primary head:**  *Clark (1994, ch. 47);Andrade et al. (2011, ch. 298).*  0. proötic, squamosal, and exoccipital  1. proötic and laterosphenoid |
| 299 | **Quadrate, position of foramen aerum, next to the articular condyle:**  *Brochu (1999, ch. 121 modified);Andrade et al. (2011, ch. 299).*  0. foramen aerum single, on mediodorsal angle of the quadrate, close to the condyle  1. foramen aerum single, on dorsal surface of the quadrate, close to the condyle  2. foramen aerum double, being the medial foramen on the mediodorsal angle of the quadrate and distant to the condyle, and the lateral foramen on the dorsal surface and close to the condyle |
| 300 | **RECODING:Quadrate condyle, size of medial hemicondyle relative to lateral hemicondyle, and presence of intercondylar groove:**  *Ortega et al. (2000, ch. 53); Andrade & Bertini (2008a, ch. 68); Turner & Buckley (2008, ch. 170);Andrade et al. (2011, ch. 300).*  0. medial hemicondyle smaller or subequal to lateral one, poorly curved, and with intercondylar groove incipient at best.  2. medial hemicondyle evidently larger than the lateral one, round, with intercondylar groove incipient |
| 301 | **Quadrate condyle, expansion of medial hemicondyle:**  *Brochu (1999, ch. 112 modifed, part);Andrade et al. (2011, ch. 301).*  0. absent  1. present, medial hemicondyle is expanded. |
| 302 | **External auditory meatus, general morphology:**  *Andrade et al. (2011, ch. 302).*  0. subpolygonal to elliptic  1. triangle-shaped, with apex directed dorsally |
| 303 | **External auditory meatus, size:**  *Andrade et al. (2011, ch. 303).*  0. very small, poorly visible (even in lateral view)  1. medium to large, conspicuous |
| 304 | **External auditory meatus, position of squamosal-quadrate suture at distal edge:**  *Brochu (1999, ch. 132);Andrade et al. (2011, ch. 304).*  0. squamosal-quadrate suture extends dorsally along posterior margin of external auditory meatus, directed anterodorsally  1. squamosal-quadrate suture extends only to posteroventral corner of external auditory meatus, directed anteroventrally or horizontal |
| 305 | **Otic aperture, morphology of distal margin:**  *Brochu (1999, ch. 102 modified); Salisbury et al. (2006, ch. 102 modified); Delfino et al. (2008, ch. 102 modified); Andrade et al. (2011, ch. 305).*  0. posterior margin not defined and gradually merging into the exoccipital, or smooth  and continuous with the paraoccipital process, but never invaginated  1. distal margin inset or invaginate |
| 306 | **Cranioquadrate canal, presence and position:**  *Clark (1994, ch. 49 modified);Andrade et al. (2011, ch. 306).*  0. absent  1. at least partially enclosed by quadrate, exoccipital and squamosal, with distal end near lateral edge of skull  2. at least partially enclosed by quadrate, exoccipital and squamosal, with distal end located ventral to paraoccipital process |
| 307 | **Cranioquadrate canal, general structure:**  *Andrade et al. (2011, ch. 307).*  0. canal laterally open or not formed, and cranioquadrate passage absent  1. passage fully formed, with canal enclosed at least distally by the exoccipital and squamosal, regardless of the participation of the quadrate  2. passage fully formed, with canal laterally enclosed by quadrate and squamosal, and exoccipital only bounding the canal medially  3. passage fully formed, with canal laterally enclosed by quadrate and exoccipital, regardless of the participation of the squamosal |
| 308 | **Cranioquadrate canal, lateral contact between quadrate and exoccipital:**  *Andrade et al. (2011, ch. 308).*  0. narrow or absent  1. broad |
| 309 | **Cranioquadrate passage, exposure in occipital view:**  *Buscalioni & Sanz (1990); Ortega et al. (2000, ch. 160 modified); Buscalioni et al. (2001, ch. 166 revised); Delfino et al. (2005, ch. 166 revised); Andrade et al. (2011, ch. 309).*  0. exposed on occipital surface  1. shielded posteriorly by the ventral border of paraoccipital process, with passage not exposed in occipital view |

**Mandible** (Ch. 310 – 366; 11.49% of characters)

| # | Description |
| --- | --- |
| 310 | **Oral symphyseal fossa, presence:**  *Andrade et al. (2011, ch. 310).*  0. absent  1. present as a small depressed area, anteroposteriorly elongated, located at the medial line of symphysis, in the oral cavity |
| 311 | **Posteroventral symphyseal fossa, presence of a single depressed area at the posterior end of symphysis, in ventral view:**  *Andrade et al. (2011, ch. 311).*  0. absent or feeble  1. evident, deep, longer than wide |
| 312 | **External mandibular fenestra, presence:**  *Clark (1994, ch. 75 modified); Ortega et al. (2000, ch. 80 revised);Andrade et al. (2011, ch. 312).*  0. absent  1. present as a diminutive passage  2. present as an evident fenestra |
| 313 | **External mandibular fenestra, size relative to the orbit:**  *based on Ortega et al. (2000, ch. 80); Andrade et al. (2011, ch. 313).*  0. extremely reduced or absent, less than 50% of the length of the orbit, with surangular and angular sutured along most of (or all) their length  1. small, approximately with the same length of the orbit  2. large, evidently longer than the orbit |
| 314 | **External mandibular fenestra, orientation of main axis:**  *Andrade et al. (2011, ch. 314).*  0. horizontal  1. main axis inclined, directed anteroventrally-posterodorsally |
| 315 | **External mandibular fenestra, shape:**  *Andrade et al. (2011, ch. 315).*  0. subcircular to poorly elliptic  1. highly elliptic, anteroposterior axis much longer than dorso-ventral axis, three time or more, but both ends rounded  2. slit-like, proportionally very long and both ends acute  3. broad teardrop-like  4. narrow teardrop-like  5. triangle |
| 316 | **External mandibular fenestra, morphology of anterior margin:**  *Andrade et al. (2011, ch. 316).*  0. curved, with a broad arched margin anteriorly  1. anterodorsal and anteroventral margins poorly arched, meeting at an acute angle  anteriorly, anterior end is wedge-like |
| 317 | **Mandible, presence of evident festooning at anterior mandible:**  *Andrade et al. (2011, ch. 317).*  0. absent, margin straight, in lateral view  1. present, projecting dorsally at the premaxilla-maxilla suture |
| 318 | **Mandible, presence of evident festooning at mid mandible:**  *after Ortega et al. (2000, ch. 21 revised); Andrade et al. (2011, ch. 318).*  0. absent, margin straight in lateral view, or moderately concave  1. present and incipient, with dorsal edge of dentary weakly sinusoidal in lateral view  2. present and evident, with dorsal edge of dentary strongly sinusoidal in lateral view |
| 319 | **Mandible, overall morphology in dorsal view:**  *Andrade et al. (2011, ch. 319).*  0. mandible is narrow, hemimandibles are confluent, with left and right alveolar margins running alongside each other  1. mandible is broad, hemimandibles are mostly parallel, but alveolar margins meet medially at first alveolus forming a wide arched line, giving the mandible a broad-U shape |
| 320 | **Mandible, orientation of hemimandibles at their medial contact:**  *Andrade et al. (2011, ch. 320).*  0. evidently acute angle, hemimandibles meet at approximately 45 degrees of each other, or less  1. broad angle, hemimandibles meet at approximately 70 degrees of each other, or more |
| 321 | **Mandible, morphology of distal rami in dorsal/ventral views:**  *Andrade et al. (2011, ch. 321).*  0. distal rami mostly straight or poorly curved  1. distal rami strongly curved medially at mid-mandible, giving the mandible a broad-Y shape |
| 322 | **Mandible, ventral border at angular, in lateral view: (ORDERED)**  *Andrade et al. (2011, ch. 322).*  0. angular straight and mostly horizontal, or poorly curved, from the anterior to the posterior end  1. angular evidently (but gently) curved  2. angular abruptly curved, always below glenoid fossa, with mid-posterior sections of angular sub-vertical, facing posteriorly |
| 323 | **Mandible, morphology of ventral margin, in lateral view:**  *Andrade et al. (2011, ch. 323).*  0. mandible is curved ventrally, with maximum curvature at anterior section of angular, below the mandibular fenestra (when present), or not curved at all  1. mandible is curved posteroventrally, with maximum curvature at posterior section of angular, below (or almost below) the mandibular glenoid fossa, usually posterior to mandibular fenestra (when present) |
| 324 | **Mandible, dorsal border at dentary-surangular contact, in lateral view:**  *Clark (1994, ch. 74); Sereno et al. (2003, ch. 41);Andrade et al. (2011, ch. 324).*  0. mostly straight  1. gently arched dorsally  2. strongly arched dorsally |
| 325 | **Mandible, presence of an evident coronoid process, projecting dorsally or anterodorsally:**  *Andrade & Bertini (2008a, ch. 112);Andrade et al. (2011, ch. 325).*  0. absent  1. present |
| 326 | **Mandible, relation between surangular and articular:**  *Brochu (1999, ch. 60);Andrade et al. (2011, ch. 326).*  0. truncated, sulcus present between surangular and articular  1. continuous, articular flush against surangular |
| 327 | **Mandible, position of caudal end of surangular-angular suture relative to articular:**  *Brochu (1999, ch. 67);Andrade et al. (2011, ch. 327).*  0. lingually meets articular at ventral tip  1. meets articular dorsal to ventral tip |
| 328 | **Mandible, morphology of angular-surangular suture and relation with external mandibular fenestra, at late ontogeny:**  *Norell (1988, ch. 40); Brochu (1999, ch. 47);Andrade et al. (2011, ch. 328).*  0. angular-surangular suture mostly horizontal, contacting fenestra at posterior angle (when fenestra is present)  1. angular-surangular suture curves ventrally at anterior end, passing broadly along ventral margin of fenestra (when fenestra is present) |
| 329 | **Mandible, presence of a splenial crest:**  *Andrade et al. (2011, ch. 329).*  0. absent  1. present as a long, laminar and horizontal blade, next to the alveolar margin, projecting medially |
| 330 | **Mandible, presence of a conspicuous and robust surangular crest on the lateral surface of the mandible, next to the glenoid fossa:**  *Turner & Buckley (2008, ch. 287); Andrade et al. (2011, ch. 330).*  0. absent  1. present |
| 331 | **Symphysis, orientation relative to the horizontal plane:**  *based on discussion by Ortega et al. (2000); Andrade et al. (2011, ch. 331).*  0. horizontal or slightly inclined  1. inclined dorsally |
| 332 | **Symphysis, length relative to width:**  *Andrade et al. (2011, ch. 332).*  0. short, length and width subequal or shorter than wide, and mandible "U" or "Vshaped"  1. proportionally long, longer than wide, and mandible "V" or "Y-shaped"  2. extremely long, length at least five times its width, and mandible "Y-shaped" |
| 333 | **Symphysis, morphology of anterior end:**  *Andrade et al. (2011, ch. 333).*  0. symphysis tapers anteriorly, with no constriction at mid-posterior sections  1. symphysis clearly constricted at fifth-sixth alveoli  2. symphysis flares anteriorly, with anterior region bearing teeth 1-4 at anterior margin and posterior region narrower (but constriction poorly defined)  3. symphysis flares anteriorly, with anterior region bearing teeth 1-2 at anterior margin and posterior region narrower (but constriction poorly defined) |
| 334 | **Symphysis, morphology of dorsal surface:**  *Pol & Apesteguia (2005, ch. 184); Turner & Buckley (2008, ch. 189); Andrade et al. (2011, ch. 334).*  0. flat or slightly concave  1. strongly concave and narrow, trough shaped |
| 335 | **Symphysis, presence of posterior splenial peg:**  *Pol & Apesteguia (2005, ch. 181);Andrade et al. (2011, ch. 335).*  0. absent  1. present |
| 336 | **Symphysis, shape of anterior end in dorsal view: (ORDERED)**  *Andrade et al. (2011, ch. 336).*  0. anterior end expanded, fan-like  1. anterior end expanded, rounded to sub-quadratic  2. not expanded |
| 337 | **Dentary, presence of an occlusal pit or strong concavity for the reception of an enlarged maxillary caniniform:**  *Buckley & Brochu (1999, ch. 105 modified); Turner & Buckley (2008, ch. 158 modified); Andrade et al. (2011, ch. 337).*  0. absent  1. present, occlusal concavity lateral to the 5th-7th alveoli  2. present, occlusal concavity lateral to the 8th-9th alveoli |
| 338 | **Dentary, morphology of distal end:**  *Clark (1994, ch. 70 modified);Andrade et al. (2011, ch. 338).*  0. dentary tappers posterodorsally into a single ramus, usually acute, extending only dorsal to the mandibular fenestra  1. dentary extends dorsally to the mandibular fenestra, and almost vertically ventral to the anterior margin of the fenestra (posteroventral ramus incipient)  2. dentary distal end bifurcated, usually extending both dorsally and ventrally to the mandibular fenestra (if fenestra present and not reduced), with posteroventral ramus evidently present and well developed |
| 339 | **Dentary, morphology of dorsal ramus at distal end, next (dorsal) to mandibular fenestra:**  *Andrade et al. (2011, ch. 339).*  0. dentary ramus dorsal to fenestra follows dorsal edge of fenestra  1. dentary ramus dorsal to fenestra projects posteroventrally as a laminar blade, partially shielding the fenestra laterally and creating a secondary, straight to slightly convex anterodorsal border. |
| 340 | **Dentary distal end, extension relative to the distal margin of the orbit:**  *Andrade et al. (2011, ch. 340).*  0. relatively short, do not reach posterior to the orbit  1. relatively long, reaches posterior to the orbit |
| 341 | **Splenials, general structure:**  *Ortega et al. (1996, ch. 7); Buckley & Brochu (1999, ch. 110); Turner & Buckley (2008, ch. 161); Andrade et al. (2011, ch. 341).*  0. thin posterior to symphysis  1. robust dorsally, posterior to symphysis |
| 342 | **Splenials, involvement in symphysis, in ventral view:**  *Clark (1994, ch. 77 modified); Brochu (1999, ch. 43 modified); Ortega et al. (2000, ch. 88 modified); Andrade et al. (2011, ch. 342).*  0. absent, splenials do not take part in the symphysis  1. present, splenials are visible at the distal end of symphysis, in ventral view |
| 343 | **Splenials, extent of involvement in symphysis:**  *Clark (1994, ch. 77 modified); Turner & Buckley (2008, ch. 77 modified); Andrade et al. (2011, ch. 343).*  0. marginal, or none at all  1. modest but evident, with length of splenials at symphysis approximately the same as the width of the symphysis  2. extensive participation, length of splenials at symphysis much longer than width ofsymphysis |
| 344 | **Splenials, morphology at their contact in the symphysis, in ventral view:**  *Pol & Apesteguia (2005, ch. 180); Turner & Buckley (2008, ch. 185); Andrade et al. (2011, ch. 344).*  0. V-shaped  1. U-shaped |
| 345 | **Splenial, participation in the medial wall of the posterior mandibular alveoli:**  *Andrade et al. (2011, ch. 345).*  0. does not take part, splenial may reach the alveolar margin, but alveoli are delimited solely by the dentary  1. participates in the distalmost alveoli, supporting teeth |
| 346 | **Surangular, extension of the anterior lateral ramus:**  *Andrade et al. (2011, ch. 346).*  0. short, does not extend beyond the orbit  1. long, extends at least to the same relative position as the anterior border of the orbit, or reaches beyond the orbit |
| 347 | **Surangular, proportional development of lateral and medial rami, at anterior end:**  *Andrade & Bertini (2008a, 2008b, ch. 113 modified); Turner & Buckley (2008, ch. 289); Andrade et al. (2011, ch. 347).*  0. medial ramus absent or incipient, with lateral ramus well developed  1. medial ramus well developed, subequal ro lateral ramus, and dentary-surangular suture evidently complex |
| 348 | **Surangular, morphology of lateral anterior process:**  *Ortega et al. (2000, ch. 96);Andrade et al. (2011, ch. 348).*  0. single  1. forked, with a dorsal and a ventral process evident |
| 349 | **Surangular, relative length of the anterior processes of the lateral anterior ramus:**  *Brochu (1999, ch. 48 modified);Andrade et al. (2011, ch. 349).*  0. unequal, dorsal process much longer, or ventral process absent  1. subequal to equal  2. unequal, ventral process longer |
| 350 | **Surangular, presence of extension to the retroarticular process:**  *Norell (1988, ch. 42 modified); Brochu (1999, ch. 51 revised); Andrade et al. (2011, ch. 350).*  0. absent, pinched off anterior to tip of retroarticular process, or surangular excluded from process  1. present, extends to posterior end of retroarticular process |
| 351 | **Coronoid, relation with foramen intermandibularis medius:**  *Norell (1988, ch. 12); Brochu (1999, ch. 46);Andrade et al. (2011, ch. 351).*  0. limited, coronoid only bounds posterior half of the foramen  1. extensive, completely surrounds foramen  2. extensive, obliterates foramen |
| 352 | **Coronoid, morphology of dorsal edge:**  *Brochu (1999, ch. 54);Andrade et al. (2011, ch. 352).*  0. slopes strongly anteriorly  1. almost horizontal |
| 353 | **Angular, presence of insertion area for M pterygoideus posterior onto its lateral surface:**  *Clark (1994, ch. 76);Andrade et al. (2011, ch. 353).*  0. absent, M pterygoideus posterior limited to the posterior/ventral surfaces of angular  1. present and evident |
| 354 | **Prearticular, presence:**  *Clark (1994, ch. 72 revised); Sereno et al. (2003, ch. 39); Andrade et al. (2011, ch. 354).*  0. absent  1. present |
| 355 | **Mandiblular glenoid fossa, length relative to width:**  *Wu & Sues (1996, ch. 23 modified); Ortega et al. (2000, ch. 105); Turner & Buckley (2008, ch. 104); Andrade et al. (2011, ch. 355).*  0. short, length smaller than width, matching the dimensions of the quadrate condyle  1. length at least equal to width, or longer, and evidently longer than the quadrate condyle |
| 356 | **Mandibular glenoid fossa, development of posterior margin:**  *Pol & Apesteguia (2005, ch. 181 modified); Andrade et al. (2011, ch. 356).*  0. posterior margin smoothly progressing to the retroarticular process, with glenoid fossa poorly defined  1. posterior margin delimited by a corner, and the glenoid fossa clearly delimited  2. posterior margin well developed, evidently high |
| 357 | **Mandibular glenoid fossa, participation of surangular in the articulation:**  *Buckley & Brochu (1999, ch. 102); Ortega et al. (2000, ch. 99 modified); Turner & Buckley (2008, ch. 156); Andrade et al. (2011, ch. 357).*  0. does not take part, or barely takes part on the lateral wall of the fossa  1. broadly participates in the glenoid fossa, forming approximately one third of its surface, with quadratojugal also broadly contributing to the quadrate condyle |
| 358 | **Retroarticular process, development:**  *Clark (1994, ch. 71 part);Andrade et al. (2011, ch. 358).*  0. absent or poorly developed  1. present and evidently projecting posterior to glenoid fossa |
| 359 | **Retroarticular process, length of the attachment surface for the adductor muscles relative to its width: (ORDERED)**  *Jouve et al. (2005, ch. 1 modified);Andrade et al. (2011, ch. 359).*  0. short, subequal  1. moderately elongated, evidently longer than wide  2. extremely elongate, more than twice its width |
| 360 | **Retroarticular process, orientation in lateral view: (ORDERED)**  *Norell & Clark (1990, ch. 7 modified); Clark (1994, ch. 71 part); Brochu (1999, ch. 50); Andrade et al. (2011, ch. 360).*  0. posteroventrally oriented  1. posteriorly oriented  2. posterodorsally oriented |
| 361 | **Retroarticular process, position of distalmost tip relative to the mandibular glenoid fossa:**  *Andrade et al. (2011, ch. 361).*  0. tip at the same level or below  1. tip clearly in a more dorsal plane than the glenoid fossa |
| 362 | **Retroarticular process, morphology and orientation in dorsal/posterior view:**  *based on Clark (1994, ch. 71 part); Andrade et al. (2011, ch. 362).*  0. surface poorly concave and facing dorsally, or at least lateral surface facing dorsallylaterodorsally and medial surface facing mediodorsally (if surface divided)  1. surface strongly concave, facing dorsomedially |
| 363 | **Retroarticular process, morphology of the surface for the attachment of adductor muscles:**  *Andrade et al. (2011, ch. 363).*  0. triangle shaped  1. ellipsoid, rectangular or spoon shaped  2. shovel shaped |
| 364 | **Retroarticular process, presence of a longitudinal anteroposteriorly oriented crest or ridge dividing the attachment surface for the adductor muscles:**  *Andrade et al. (2011, ch. 364).*  0. absent  1. present, dividing the surface into medial and lateral portions. |
| 365 | **Retroarticular process, position of the posteromedial wing:**  *Jouve et al. (2005, ch. 2); Jouve et al. (2006, ch. 179);Andrade et al. (2011, ch. 365).*  0. posteromedial wing dorsally situated, or at mid height on the retroarticular process  1. posteromedial wing ventrally situated on the retroarticular process |
| 366 | **Retroarticular process, position and orientation of the foramen aerum:**  *Norell (1988, ch. 16 modified); Brochu (1999, ch. 49); Andrade et al. (2011, ch. 366).*  0. foramen aerum medially oriented, opening at the medial margin of retroarticular process lamina  1. foramen aerum dorsally oriented, lateral from the medial margin of retroarticular process |

**Dentition and alveolar morphologies** (Ch. 367 – 418; 10.5% of characters)

| # | Description |
| --- | --- |
| 367 | **Dentition, relation between tooth rows on both sides of the skull:**  *Novas et al. (2009);Andrade et al. (2011, ch. 367).*  0. forming one continuous set of teeth, both in the cranium and mandible  1. forming two distinct sets, tooth rows at posterior set convergent rostrally and almost in touch each other, at mid-palate and mandible |
| 368 | **Posterior maxillary teeth, transverse section:**  *Buckley et al. (2000, ch. 116 modified); Ortega et al. (2000, ch. 104 modified); as in Andrade & Bertini (2008, ch. 135); Andrade et al. (2011, ch. 368).*  0. evident lateral compression affecting both edges of the crown, making both edges evident regardless of the presence/absence of carinae/keel  1. transverse section circular to subcircular, without significant lateral compression  2. transverse section 'teardrop-like' (=triangular), with asymmetric lateral compression occurring on the distal margin only |
| 369 | **Mid to posterior mandibular teeth, transverse section:**  *Buckley et al. (2000, ch. 116 modified); Ortega et al. (2000, ch. 104 modified); as in Andrade & Bertini (2008, ch. 146); Andrade et al. (2011, ch. 369).*  0. evident lateral compression affecting the entire crown, making evident both mesial and distal edges, regardless of the presence/absence of carinae/keel  1. transverse section circular to subcircular, without significant lateral compression  2. transverse section 'teardrop-like' (=triangular), with asymmetric lateral compression occurring on the mesial margin only |
| 370 | **Dentition, presence of facetted teeth:**  *Young & Andrade (2009, ch. 130);Andrade et al. (2011, ch. 370).*  0. absent  1. present, most crowns with at least the labial surface with three facets |
| 371 | **Dentition, presence of laminar teeth:**  *Andrade et al. (2011, ch. 371).*  0. absent  1. present, laminar teeth dominate dentition |
| 372 | **Dentition, presence of spatulated teeth:**  *Buckley et al. (2000, ch. 116 modified);Andrade et al. (2011, ch. 372).*  0. absent  1. present |
| 373 | **Anterior to mid dentition, general crown robustness:**  *Andrade et al. (2011, ch. 373).*  0. teeth slender, sharpening apically  1. teeth robust, "inflated", or bulbous apically |
| 374 | **Mid to posterior dentition, presence of pebbled ornamentation on tooth crown surface:**  *Andrade et al. (2011, ch. 374).*  0. absent  1. present, enamel ornamented with a peebled pattern |
| 375 | **Mid to posterior dentition, presence and morphology of ridged ornamentation on enamel surface of teeth:**  *Andrade & Bertini (2008a, ch. 123 modified); Andrade et al. (2011, ch. 375).*  0. enamel ornamentation absent or incipient  1. present, composed of basi-apical well-defined ridges, conspicuous and set apart to each other, never anastomosed  2. present, composed of numerous basi-apical well-defined ridges, conspicuous and set close to each other, rarely anastomosed, with anastomosis stronger apically  3. present, composed of numerous basi-apical low ridges, feeble and set close to each other, poorly anastomosed, with anastomosis stronger apically  4. present, composed of numerous basi-apical low ridges, feeble and set close to each other, anastomosed into a fabric of ridges distributed trough most of the crown |
| 376 | **Mid to posterior dentition, presence of accessory ridges on labial-lingual surfaces of crown:**  *Andrade et al. (2011, ch. 376).*  0. absent  1. present, basi-apical, evident and well-spaced, formed by enamel and dentine |
| 377 | **Mid to posterior dentition, number of cusps per tooth:**  *Gomani (1997, ch. 46 modified); Buckley et al. (2000, ch. 113 modified); Pol (2003, ch. 162 modified); Turner & Buckley (2008, ch. 188 modified); Andrade et al. (2011, ch. 377).*  0. each crown has single apical cusp, regardless of presence of accessory cusps in cingula  1. each crown has one main cusp aligned with smaller cusps, arranged in a single row  2. several cusps, unequal in size, arranged in more than one row  3. multiple small cusps, subequal in size, along edges of occlusal surface |
| 378 | **Carinae, presence of keel at the edge of tooth crown:**  *Andrade et al. (2011, ch. 378).*  0. absent  1. present |
| 379 | **Carinae (mid-posterior dentition), presence and morphology of denticles at crown edges:**  *Buckley et al. (2000, ch. 104 modified); Sereno et al. (2003, ch. 53 modified); Andrade & Bertini (2008a, ch. 132 revised); Andrade et al. (2011, ch. 379).*  *True denticles and false-ziphodont crenulations (=enamel ornamentation) as in Prasad &*  *Broin (2002); ziphomorph as in Andrade & Bertini (2008c).*  0. homogenous carina, serrated with cuneiform to ripple-like true denticles (ziphodont)  1. true denticles absent, crowns either with a smooth edge (non-ziphodont), or a homogenous carina where crenulations may appear as a result of superficial ornamentation (false-ziphodont)  2. heterogeneous carina, composed by tubercle-like true denticles (ziphomorph) |
| 380 | **Carinae (maxillae), distribution of denticles at crown edges:**  *based on Price (1950) and Pol (2003); Andrade & Bertini (2008a, ch. 132 modified); Andrade et al. (2011, ch. 380).*  0. mesial and distal crown edges with the same morphology, either with or without true denticles  1. mesial carina absent and distal carina present |
| 381 | **Carinae (mid-posterior mandible), distribution of denticles at crown edges:**  *Andrade & Bertini (2008a, ch. 132 modified);Andrade et al. (2011, ch. 381).*  0. mesial and distal crown edges with the same morphology, either with or without true denticles  1. mesial carina present and distal carina absent, with mid-posterior teeth ocluding as opposing blades |
| 382 | **Occlusion, relation between premaxillary and mandibular dentitions:**  *Andrade et al. (2011, ch. 382).*  0. either match the mandible or slightly cover it, as upper teeth overbite the dentary teeth  1. premaxilla widely overhangs the mandible, with premaxillary ventral margin covering the alveolar margin at anterior mandible |
| 383 | **Occlusion, relation between maxillary and mandibular series at mid dentition:**  *Andrade et al. (2011, ch. 383).*  0. in-line or interlocked  1. maxillary dentition overbites mandibular dentition |
| 384 | **Occlusion, relation between maxillary and mandibular series at the posterior dentition:**  *Andrade et al. (2011, ch. 384).*  0. in-line or interlocked  1. maxillary dentition overbites mandibular dentition |
| 385 | **Premaxillary teeth, presence of a hypertrophied tooth at penultimate or last alveolus:**  *Andrade & Bertini (2008a, ch. 125 modified);Andrade et al. (2011, ch. 385).*  0. all teeth subequal in size  1. one enlarged tooth, longer than the other premaxillary elements, usually not higher than the symphyseal depth  2. one fully hypertrophied tooth, much longer than the other premaxillary elements and at least as high as the symphyseal depth, also with a much larger cross-sectional area at crown base |
| 386 | **Premaxillary enlarged tooth, size relative to largest teeth at maxillae and mandible:**  *Andrade et al. (2011, ch. 386).*  0. premaxillary tooth smaller, with shorter crown and shorter-narrower alveoli, or subequal  1. premaxillary tooth larger than any given tooth at maxilla or mandible, with higher crown and longer-wider alveolus |
| 387 | **Premaxillary alveolar margin, orientation:**  *Sereno et al. (2001, 2003, ch. 68 modified); Turner & Buckley (2008, ch. 239 modified); Andrade et al. (2011, ch. 387).*  *Note that scorings here are based on Andrade et al. (2011), which strongly diverge from the original.*  0. vertical, dentition is procumbent or not  1. inturned, dentition is not procumbent |
| 388 | **Premaxillary alveolar margin, projection relative to the maxillary alveolar border:**  *based on Wu et al. (2001b); after Sereno et al. (2001, 2003, ch. 71);Andrade et al. (2011, ch. 388).*  0. absent, alveolar margin of premaxillae and maxillae continuous, usually in the same plane  1. present, premaxillary alveolar margin is ventrally offset |
| 389 | **Premaxillary alveolar margin, distinction relative to the maxillary alveolar border, in lateral view:**  *Andrade et al. (2011, ch. 389).*  0. alveolar margin is continuous, and maxillary dentition is no more than slightly offset ventrally  1. premaxillary and maxillary alveolar margins are separated by a extremely deep notch, with maxillary dentition distinctly offset ventrally |
| 390 | **Premaxillary alveoli, disposition in ventral view:**  *Sereno et al. (2001, ch. 69 modified); Turner & Buckley (2008, ch. 240 modified); Andrade et al. (2011, ch. 390).*  0. alveoli set in the alveolar margin to form an arched row, curved posteriorly from midline and poorly diverging from medial line, usually angled laterally at approximately 90 degrees or less  1. alveoli set in the alveolar margin to form a straight to poorly arched row, strongly diverging from medial line and angled laterally at approximately 120 degrees |
| 391 | **Last premaxillary tooth, relative position in the horizontal plane:**  *Sereno et al. (2001, 2003, ch. 70 modified); Turner & Buckley (2008, ch. 241 modified); Andrade et al. (2011, ch. 391).*  0. strongly anteromedial to first maxillary tooth  1. anterior to first maxillary tooth, or slightly altered relative to it  2. evidently anterolateral to first maxillary tooth (posterolateral) |
| 392 | **Maxillary dentition at anterior maxillae, presence of a hypertrophied caniniform tooth:**  *Andrade & Bertini (2008a, ch. 130 modified);Andrade et al. (2011, ch. 392).*  0. absent, no enlarged caniniform is present, with maxillary dentition usually isometric to sub-isometric  1. present as an enlarged tooth slightly larger then (but not contrasting with) neighboring teeth, with maxillary dentition anisometric  2. one hypertrophied tooth, much larger than neighboring teeth and contrasting in size with them, with maxillary dentition strongly anisometric |
| 393 | **Maxillary dentition, morphology:**  *Andrade & Bertini (2008a, ch. 130 modified);Andrade et al. (2011, ch. 393).*  0. all maxillary teeth caniniform, or last teeth lanceolate (isomorphic or sub-isomorphic)  1. acute caniniforms anteriorly, followed by blunter caniniform teeth  2. caniniform teeth anteriorly, followed by molariform teeth  3. most or all teeth molariform, but teeth 1-2 eventually weakly caniniform to conical |
| 394 | **Maxillary tooth row, extension relative to anterior border of suborbital fenestra:**  *Ortega et al. (2000, ch. 18 part);Andrade et al. (2011, ch. 394).*  0. does not reach the anteriormost border of the suborbital fenestra  1. reaches the anteriormost border of the suborbital fenestra  2. extends posterior to the anteriormost border of the suborbital fenestra |
| 395 | **Maxillary tooth row, position of last maxillary tooth relative to posterior border of suborbital fenestra:**  *Ortega et al. 2000, ch18 part);Andrade et al. (2011, ch. 395).*  0. far anterior to the posteriormost end of the suborbital fenestra  1. at the same relative position of the posterior border of the suborbital fenestra, or very close |
| 396 | **Maxillary/dentary teeth, implantation of anterior to middle elements, at maturity:**  *Ortega et al. (2000, ch. 19 modified); Turner & Buckley (2008, ch. 164 modified); Andrade et al. (2011, ch. 396).*  0. teeth set in fully isolated alveoli  1. at least part of the teeth set in a groove, not separated by septa |
| 397 | **Maxillary/dentary teeth waves, in non-tubular-snouted forms:**  *based on Clark (1994, ch. 79); as in Turner & Buckley (ch. 79 modified); Andrade et al. (2011, ch. 397).*  0. absent, no tooth size variation  1. one wave of teeth enlarged, at mid snout  2. enlarged teeth in two waves  3. one wave of teeth greatly enlarged, at the end of maxilla/dentary |
| 398 | **Symphyseal dentition, alignment of anteriormost alveoli relative to the medial line:**  *Andrade et al. (2011, ch. 398).*  0. alveoli 1-4 not transversally aligned at anterolateral margin  1. alveoli 1-2 transversally aligned, and following alveoli set posteriorly to them  2. alveoli 1-4 transversally aligned, so the fourth alveolus is lateral (or lateral and slightly posterior) to first alveolus, and following alveoli are posterior to them |
| 399 | **Symphyseal dentition, position of fifth alveoli in dorsal (buccal) view:**  *Andrade et al. (2011, ch. 399).*  0. distant, or at least both alveoli moderately apart  1. close to each other, next to the medial line of symphysis |
| 400 | **Symphyseal dentition, presence of a complete symphyseal tooth battery:**  **Andrade & Bertini 2008a, ch141);** *Andrade et al. (2011, ch. 400).*  0. absent  1. present, teeth from each pair closer to each other than to other teeth in the same hemimandible |
| 401 | **Symphyseal dentition, presence of highly procumbent teeth:**  *Andrade & Bertini (2008a, ch. 140 modified);Andrade et al. (2011, ch. 401).*  0. non-procumbent to mildly procumbent  1. first symphyseal pair highly procumbent, crowns nearly horizontal  2. pairs 1-2 highly procumbent, crowns nearly horizontal |
| 402 | **Symphyseal alveoli 1-2, confluence:**  *based on Brochu (1999, ch. 52); Andrade et al. (2011, ch. 402).*  0. well-separated, usually as much distant from each other as from other mandibular teeth  1. alveoli 1-2 confluent, separated by a thin alveolar wall, and clearly apart from neighboring alveoli |
| 403 | **Symphyseal alveoli 3-4, confluence:**  *Brochu (1999, ch. 52 modified, part);Andrade et al. (2011, ch. 403).*  0. well-separated, usually as much distant from each other as from other mandibular teeth  1. alveoli 3-4 confluent, separated by a thin alveolar wall, and clearly apart from neighboring alveoli |
| 404 | **Symphyseal alveoli 3-4, relative size:**  *Brochu (1999, ch. 52 modified, part);Andrade et al. (2011, ch. 404).*  0. nearly same size, or third alveolus larger  1. fourth alveolus larger than third |
| 405 | **Symphyseal alveoli 3-4, relative position:**  *Andrade et al. (2011, ch. 405).*  0. tooth 3 medial to tooth 4  1. tooth 3 anteromedial to tooth 4  2. teeth 3-4 set in tandem |
| 406 | **Symphyseal alveolus 1, relative position:**  *Andrade et al. (2011, ch. 406).*  0. not in line with alveoli 3-4, closer to the medial line of symphysis  1. in line with alveoli 3-4 |
| 407 | **Symphyseal alveolus 2, relative position:**  *Andrade et al. (2011, ch. 407).*  0. not in line with alveoli 3-4 and closer to the medial line  1. in line with alveoli 3-4, as close as these to the medial line  2. not in line with alveoli 3-4, at a more lateral position |
| 408 | **Dentary tooth opposite to premaxilla-maxilla contact, isometry:**  *based on Clark (1994, ch. 80); Andrade et al. (2011, ch. 408).*  0. subequal to other neighboring teeth  1. tooth is at least evidently enlarged, anisometric relative to other neighboring teeth |
| 409 | **Dentary tooth opposite to premaxilla-maxilla contact, length:**  *Clark (1994, ch. 80); Sereno et al. (2003, ch. 54); Andrade & Bertini (2008a, ch. 142);Andrade et al. (2011, ch. 409).*  0. small to medium sized, but length is no more than twice the length of other neighboring teeth  1. hypertrophied, at least twice longer than neighboring teeth |
| 410 | **Dentary tooth opposite to premaxillary-maxillary suture, occlusion:**  *Norell (1988, ch. 29); Brochu (1999, ch. 77 modified); Andrade et al. (2011, ch. 410).*  0. occludes either in notch at premaxilla and maxilla early in ontogeny, or lateral to premaxilla-maxilla suture, when the notch is absent or poorly defined  1. occludes in a pit between premaxilla and maxilla; no notch early in ontogeny  2. occludes medial to premaxilla-maxilla suture, but not in a pit or a notch |
| 411 | **Dentary tooth occluding against premaxillary-maxillary suture:**  *based on Norell (1988, ch. 29) and Clark (1994, ch. 80) and Brochu (1999, ch. 77); Andrade et al. (2011, ch. 411).*  0. third, or anterior  1. fourth  2. fifth, or posterior |
| 412 | **Maxillary dentition, area occupied by teeth and alveolar margin of maxilla, in palatal view:** *Andrade & Bertini (2008a, ch. 131 modified); Andrade et al. (2011, ch. 412).*  0. proportionally small teeth set in a narrow alveolar margin, marginal to palate/oral cavity  1. proportionally large teeth set in a wide alveolar margin, occupying large area at the maxillary ventral ramus/oral cavity |
| 413 | **Mandibular teeth 7-8, relation with the neighboring teeth:**  *Andrade et al. (2011, ch. 413).*  0. not particularly distinct  1. forming a distinct set, with alveoli closer to each other than to other teeth, and crown from tooth 7 much smaller than crown 8  2. teeth 7-8 are distant from each other, but alveoli 6-7 and 8-9 forming isolated sets, and alveoli 7-8 smaller than other alveoli |
| 414 | **Maxillary teeth, occurrence of bilateral paramesial rotation: (ORDERED)**  *Pol (2003, ch. 137 modified); Andrade & Bertini (2008a, ch. 133);Andrade et al. (2011, ch. 414).*  0. absent  1. bilateral paramesial rotation up to 30 degrees from the original plane  2. bilateral paramesial rotation clearly over 30 degrees from the original plane |
| 415 | **Middle and posterior mandibular teeth, occurrence of bilateral paramesial rotation:**  *Andrade & Bertini (2008a, ch. 144);Andrade et al. (2011, ch. 415).*  0. not oblique or slightly altered  1. oblique (more than 30 degrees). |
| 416 | **Middle and posterior teeth, occurrence of bilateral paradistal rotation:**  *Andrade et al. (2011, ch. 416).*  0. absent  1. bilateral paradistal rotation present, teeth obliquely implanted, with rotation of at least 30-40 degrees from the original plane |
| 417 | **Middle and posterior teeth, presence of cingula with accessory cusps:**  *Andrade & Bertini (2008a, ch. 149 modified);Andrade et al. (2011, ch. 417).*  0. absent  1. present, cingulum bearing a series small of cusps, set labial/lingual to the main body of crown |
| 418 | **Posterior teeth, presence of rings of undulating enamel on crown surface:**  *Gasparini et al. (2006, ch. 242);Andrade et al. (2011, ch. 418).*  0. absent  1. present |

**Axial post-cranial skeleton** (Ch. 419 – 437; 3.83% of characters)

| # | Description |
| --- | --- |
| 419 | **Vertebrae, presence of strong procoely:**  *based on Salisbury et al. (2006) and Norell & Clark (1990); Andrade et al. (2011, ch. 419).*  0. absent, vertebrae no more than feebly procoelic  1. present in all cervical, dorsal and proximal caudals, with degree of procoely progressively decreasing in distal caudals |
| 420 | **Presacral vertebrae, morphology of articular surfaces:**  *Clark (1994, ch. 92+93); Brochu (1999, ch. 18);Andrade et al. (2011, ch. 420).*  *See also Buscalioni & Sanz (1990), Ortega et al. (2000), Schwarz (2003).*  0. all amphiplatic or amphicoelic  1. presacral series includes at least gently procoelic vertebrae |
| 421 | **Presacral vertebrae, presence of a ventraly projecting laminar process (hypapophysis) ventral to the centrum:**  *Clark (1994, ch. 91 modified); Wu & Sues (1996, ch. 37 modified); Andrade & Bertini (2008a, ch. 158+159);Andrade et al. (2011, ch. 421).*  0. absent or incipient, but neither laminar nor projecting ventrally, no more than a sagittal ridge  1. present as fully projecting laminae |
| 422 | **Caudal vertebrae, morphology of articular surfaces of proximal elements:**  *Clark (1994, ch. 94 modified);Andrade et al. (2011, ch. 422).*  0. amphiplatic or amphicoelic  1. all at least gently procoelic, only with first caudal eventually biconvex |
| 423 | **Axis, proportional length of the main body of the centrum relative to its height:**  *Andrade & Bertini (2008a, ch. 151);Andrade et al. (2011, ch. 423).*  0. short, length and height of centrum subequal  1. long, centrum evidently longer than high |
| 424 | **Axis, development of neural spine laminae:**  *Andrade & Bertini (2008a, ch. 152);Andrade et al. (2011, ch. 424).*  0. poorly developed, limited to the posterior half of the neural arch  1. well developed, occupying the dorsal surface of the neural arch and projecting anteriorly and posteriorly to it, due to the presence of prespinal and postspinal laminae |
| 425 | **Axis, morphology of posterior half of neural spine:**  *Brochu (1999, ch. 3); Turner & Buckley (2008, ch. 258);Andrade et al. (2011, ch. 425).*  0. wide  1. narrow |
| 426 | **Axial hypapophysis, presence of deep fork:**  *Brochu (1999, ch. 19); Turner & Buckley (2008, ch. 259);Andrade et al. (2011, ch. 426).*  0. present  1. absent or feeble |
| 427 | **Third cervical vertebra (CIII), development of prezygapophysis:**  *Andrade & Bertini (2008a, ch. 155);Andrade et al. (2011, ch. 427).*  0. poorly developed, slightly projecting anterior to the vertebral centrum  1. well developed, clearly projecting anteriorly, beyond the vertebral centrum |
| 428 | **Anterior (postaxial) cervical vertebrae, development of neural spine laminae:**  *Clark (1994, ch. 90 modified); Pol (2003, ch. 90 modified); Andrade & Bertini (2008a, ch. 153 revised); Andrade et al. (2011, ch. 428).*  0. laminae absent or poorly developed, with neural spine rod-shaped or poorly flattened laterally  1. prespinal and postspinal laminae well developed, with neural spine occupying at least most of the dorsal surface of the neural arch |
| 429 | **Posterior cervical vertebrae, development of neural spine laminae:**  *Clark (1994, ch. 90 modified); Pol (2003, ch. 90 modified); Andrade & Bertini (2008a, ch. 156 revised); Andrade et al. (2011, ch. 429).*  0. laminae absent or poorly developed, with neural spine rod-shaped or only slightly flattened laterally  1. well developed, laminar, occupying at least most of the dorsal surface of the neural arch |
| 430 | **Anterior cervical vertebrae, structure of the base of neural spine:**  *Andrade & Bertini (2008a, ch. 154);Andrade et al. (2011, ch. 430).*  0. gracile base, with neural spine clearly distinct from the neural arch  1. robust base, with the development of spinozygapophyseal ridges |
| 431 | **Posterior cervical vertebrae, structure of the base of neural spine:**  *Andrade & Bertini (2008a, ch. 157);Andrade et al. (2011, ch. 431).*  0. gracile base, with neural spine clearly distinct from the neural arch  1. robust base, with the development of spinozygapophyseal ridges |
| 432 | **Sacral vertebrae, number:**  *Buscalioni & Sanz (1988, ch. 44 modified); Pol & Apesteguia (2005, ch. 115 modified); Andrade et al. (2011, ch. 432).*  0. two  1. three, being the third the first caudal |
| 433 | **Sacral vertebrae, orientation of the transverse processes:**  *Gasparini et al. (2006, ch. 255 modified); Young & Andrade (2009, ch. 82 modified); Andrade et al. (2011, ch. 433).*  0. horizontal  1. arched ventrally, at least in the first sacral |
| 434 | **Sacral vertebrae, relative position of lateral end of transverse process:**  *based on descriptions and data by Andrews (1913), Gasparini et al. (2006, ch. 255), Pierce & Benton (2006), and Young & Andrade (2009, ch. 82); Andrade et al. (2011, ch. 434).*  0. level with the vertebral centrum  1. ventral relative to the vertebral centrum, transverse processes of both sacrals lateroventrally directed |
| 435 | **Caudal vertebrae, relative height of neural spine:**  *based on Schwarz et al. (2006) and Schwarz-Wings et al. (2009); Andrade et al. (2011, ch. 435).*  0. larger spines are up to 2.5 times the height of vertebral body  1. average spines are 2.5-4 times the height of vertebral body |
| 436 | **Tail, vertebrae morphology near distal end:**  *Young & Andrade (2009, ch. 61);Andrade et al. (2011, ch. 436).*  0. non-hypocercal, distal vertebrae isomorphic to poorly heteromorphic  1. hypocercal, caudal series clearly heteromorphic, with a section of the distal vertebrae defining the lower lobe of a tail fin |
| 437 | **Atlantal ribs, presence of very thin medial laminae at anterior end:**  *Brochu (1999, ch. 16);Andrade et al. (2011, ch. 437).*  0. absent  1. present |

**Appendicular skeleton** (Ch. 438 – 471; 6.85% of characters)

| # | Description |
| --- | --- |
| 438 | **Scapulocoracoid synchondrosis, precocious closure during ontogeny:**  *Brochu (1999, ch. 24); Andrade et al. (2011, ch. 438)*  0. absent, synchondrosis closes very late in ontogeny  1. present, synchondrosis closes relatively early in ontogeny |
| 439 | **Scapula, symmetry:**  *Clark (1994, ch. 82); Brochu (1999, ch. 22); Ortega et al. (2000, ch. 120 modified); Lauprasert et al. (2007, ch. 66); Andrade et al. (2011, ch. 439)*  0. symmetrical, anterior and posterior edges similar in lateral view, with dorsal end poorly flared  1. asymmetrical, anterior edge strongly concave relative to posterior edge, with distal end strongly flared |
| 440 | **Coracoid, length relative to the length of scapula:**  *Clark (1994, ch. 83); Ortega et al. (2000, ch. 121); Lauprasert et al. (2007, ch. 67); Andrade et al. (2011, ch. 440)*  0. smaller, approximately half the length of the scapula  1. subequal |
| 441 | **Ilium, relative length of anterior and posterior processes:**  *Clark (1994, ch. 84); Lauprasert et al. (2007, ch. 68); Andrade et al. (2011, ch. 441)*  0. subequal, anterior and posterior processes similar in length  1. unequal, with anterior process relatively small, one quarter or less than the length of the posterior process |
| 442 | **Ilium, presence of indentation at the dorsal margin of iliac blade:**  *Brochu (1999, ch. 28 modified, part); Andrade et al. (2011, ch. 442)*  0. absent, dorsal edge convex or straight in lateral view  1. present as a shallow or modest dorsal indentation  2. present as a strong dorsal indentation ("wasp-waisted") |
| 443 | **Ilium, morphology of anterior process of iliac blade, in lateral view:**  *Brochu (1999, ch. 28 modified, part); Andrade et al. (2011, ch. 443)*  0. very narrow relative the main body of the iliac blade  1. rounded and moderately broad relative the main body of the iliac blade  2. very broad and deep, at least half the height of the main body of the iliac blade |
| 444 | **Ilium, presence of a distinct 'bulge' that fuses the anterior regions of the supraacetabular and dorsal iliac crests:**  *Ristevski et al. (2018, ch.444)*  0. anterior region of the supraacetabular crest does not fuse with the anterior margin of the iliac dorsal crest, as there is no anterior 'bulge'  1. anterior region of the crest bulges laterally (slightly overhanging the acetabular fossa), and is contiguous with the anterior margin of the iliac dorsal crest |
| 445 | **Ilium, postacetabular (=posterior) process, presence of constrictions (‘wasp-waisting’) on both the dorsal and ventral margins near the distal terminus:**  *Ristevski et al. (2018, ch. 445)*  0. absent  1. present |
| 446 | **Ischium, presence of pubic process:**  *Andrade et al. (2011, ch. 444) – reformulated from Clark (1994, ch. 86) and Andrews (1913).*  0. pubic process absent, or incipient and small, not restricting the participation of the pubis to the acetabulum  1. anterior process well developed, robust and with a round head, at least partially restricting the participation of pubis in the acetabulum |
| 447 | **Pubis, exclusion from acetabulum:**  *Andrade et al. (2011, ch. 445) – based on Andrews (1913) and Clark (1994, ch. 86).*  0. pubis not excluded, participating at least marginally of the anteroventral rim of acetabulum  1. pubis excluded, acetabulum composed exclusively by ischium and ilium |
| 448 | **Pubis, presence of exclusive proximal contact with ischium:**  *Andrade et al. (2011, ch. 446) – based on Andrews (1913) and Clark (1994, ch. 86).*  0. absent, pubis supported by both ilium and ischium  1. present, proximal head of pubis contacts only the ischium |
| 449 | **Pubis, expansion of distal end:**  *Clark (1994, ch. 85); Andrade et al. (2011, ch. 447).*  0. absent, pubis rod-like  1. present, paddle-like |
| 450 | **Limb bones, length relative to trunk, at maturity: (ORDERED)**  *Brochu (1999, ch. 33 modified); Andrade et al. (2011, ch. 448).*  0. limb bones relatively short  1. limb bones moderately long  2. limb bones very long |
| 451 | **Limb bones, general structure:**  *Brochu (1999, ch. 33 part); Andrade et al. (2011, ch. 449).*  0. limb bones robust  1. limb bones overall slender, but not weak  2. gracile |
| 452 | **Limb bones, relative length of forelimbs/hindlimbs:**  *Brochu (1999, ch. 33 part); Andrade et al. (2011, ch. 450).*  0. forelimb much shorter than hindlimb at maturity  1. forelimb slightly shorter than hindlimb at maturity  2. forelimb and hindlimb subequal in length at maturity |
| 453 | **Limb bones, general morphology of manus and pes:**  *Andrade et al. (2011, ch. 451).*  0. plantigrade or digitigrade  1. paddles |
| 454 | **Limb bones (forelimbs), proportional length of ulna relative to the humerus: (ORDERED)**  *Andrade et al. (2011, ch. 452).*  0. ulna clearly longer than humerus  1. ulna subequal to humerus (distal/proximal = 75-125%)  2. ulna clearly shorter than the humerus |
| 455 | **Limb bones (hindlimbs), proportional length of tibia relative to the femur: (ORDERED)**  *Young (2006, ch. 44 modified); Wilkinson et al. (2008, ch. 73 modified); Young & Andrade (2009, ch. 73 modified); Andrade et al. (2011, ch. 453 modified); Young et al. (2012, ch. 225 + 231 modified); Young et al. (2016, ch. 278 modified).*  0. tibia subequal to femur, or only slightly shorter (distal/proximal >74%)  1. length uneven, tibia evidently shorter than the femur (distal/proximal c. 50-74%)  2. length uneven, tibia evidently shorter than the femur (distal/proximal c. 40-50%)  3. length uneven, tibia evidently shorter than the femur (distal/proximal c. 30-40%)  4.length uneven, tibia evidently shorter than the femur (distal/proximal less than 30%) |
| 456 | **Humerus, relative orientation between the proximal and distal heads:**  *Ortega et al. (2000, ch. 181); Andrade et al. (2011, ch. 454).*  0. unaligned, each turned more than 30 degrees  1. mostly aligned, each turned no more than 30 degrees |
| 457 | **Femur, relative orientation between the proximal and distal heads:**  *Ortega et al. (2000, ch. 149), Andrade et al. (2011, ch. 455).*  0. femur with light torsion, proximal and distal articulation facets approximately at 30 degrees or less from each other  1. femur with evident torsion, proximal and distal articulation facets approximately at 60 degrees from each other |
| 458 | **Humerus, presence of common insertion for *M. teres major* and *M. dorsalis*:**  *Brochu (1999, ch. 29); Turner & Buckley (2008, ch. 261); Andrade et al. (2011, ch. 456).*  0. absent, separate scars can be distinguished dorsal to deltopectoral crest  1. present, insert with common tendon, with a single insertion scar |
| 459 | **Ulna, morphology of olecranon process:**  *Brochu (1999, ch. 27); Turner & Buckley (2008, ch. 260); Andrade et al. (2011, ch. 457)*  0. narrow and subangular  1. wide and rounded |
| 460 | **Proximal carpals, general morphology of radiale and ulnare**  *Clark (1986); Wu & Sues (1996, ch. 40); Ortega et al. (2000, ch. 129); Pol & Apesteguia (2005, ch. 110); Turner & Buckley (2008, ch. 110); Andrade et al. (2011, ch. 458).*  0. radiale and ulnare short, almost spherical  1. radiale and ulnare at least poorly elongated |
| 461 | **Proximal carpals, relative proportions of radiale:**  *Ortega et al. (2000, ch. 127); Andrade et al. (2011, ch. 459).*  0. slender, much longer than wide  1. broad, proximal width subequal to length |
| 462 | **Proximal carpals, length of radiale relative to length of metacarpals:**  *Andrade et al. (2011, ch. 460).*  0. radiale is shorter than metacarpals, or subequal  1. radiale is evidently longer than metacarpals |
| 463 | **Proximal carpals, relative length of radiale and ulnare:**  *Ortega et al. (2000, ch. 128); Andrade et al. (2011, ch. 461).*  0. radiale and ulnare subequal in length  1. radiale evidently longer than ulnare |
| 464 | **Proximal carpals, relative expansion of proximal and distal heads of radiale:**  *Buscalioni & Sanz (1988, ch. 54); Ortega et al. (2000, ch. 150); Pol & Apesteguia (2005, ch. 117); Andrade et al. (2011, ch. 462).*  0. almost equally expanded  1. proximal head wider than distal one |
| 465 | **Proximal carpals, presence of a facet of articulation in the radiale, for reception of the ulnare:**  *Pol (2005); Andrade et al. (2011, ch. 463).*  0. absent  1. present, facet evident, near its proximal end |
| 466 | **Femur, general shape:**  *Andrade et al. (2011, ch. 464).*  0. poorly sigmoid  1. strongly sigmoid |
| 467 | **Pes, relative length of digits III and IV:**  *Wilkinson et al. (2008, ch. 77); Young & Andrade (2009, ch. 77); Andrade et al. (2011, ch. 465); Young et al. (2012, ch. 229); Young et al. (2016, ch. 283).*  *Usually, digits are III-IV-II-I (descending order).*  0. digit III is longer than digit IV  1. digit IV is longer than digit III (digit IV elongated, helping to create a paddle) |
| 468 | **Calcaneum tuber, development:**  *Young & Andrade (2009, ch. 74); Andrade et al. (2011, ch. 466); Young et al. (2012, ch. 226); Young et al. (2016, ch. 279 - rephrased).*  0. well developed with a long neck (typically subequal in length to main body of calcaneum)  1. poorly developed with a short neck (less than half length of calcaneum main body, and projects out in one plane from the calcaneum main body) |
| 469 | **Metatarsal I, morphology of proximal end: (ORDERED)**  *Young & Andrade (2009, ch. 76); Andrade et al. (2011, ch. 467 modified); Young et al. (2012, ch. 228); Young et al. (2016, ch. 281).*  0. proximal end not enlarged (no more than 10% wider than any other metatarsal)  1. proximal end enlarged (20-30% wider)  2. proximal end moderately enlarged (45-55% wider)  3. proximal end greatly enlarged (>75% wider) |

**Osteoderms** (Ch. 472 – 492; 4.23% of characters)

| # | Description |
| --- | --- |
| 470 | **Ornamentation (osteoderms), type of sculpture:**  *(Ortega et al. 2000, ch111); Andrade et al. (2011, ch. 19).*  0. vermiform-dendritic pattern  1. pitted pattern |
| 471 | **Dermal armour, presence and distribution:**  *Clark (1994, ch. 100 modified); Brochu (1999, ch. 39 part); Andrade et al. (2011, ch. 468).*  0. absent  1. dorsal osteoderms present, but ventral osteoderms absent  2. dorsal and ventral osteoderms present |
| 472 | **Nuchal armour, relation of nuchal osteoderms with the remaining dorsal armour and skull:**  *Brochu (1999, ch. 38 modified, part); Andrade et al. (2011, ch. 469).*  0. large nuchal shields continuous from postoccipital region to trunk armour, with any given osteoderm contacting the anterior and posterior elements (except for the first postoccipital shield)  1. large nuchal shields continuous with trunk armour, but not reaching the postoccipital region  2. large nuchal shields discontinuous with dorsal trunk armour and absent from postoccipital region |
| 473 | **Nuchal armour, number and arrangement of nuchal shields:**  *Brochu (1999, ch. 38 modified and revised, part); Andrade et al. (2011, ch. 470).*  0. four paramedian nuchal shields, sided by two accessory shields, all enlarged relative to the remaining neck dermal armour  1. four paramedian nuchal shields enlarged relative to remaining neck shields, and no accessory shield enlarged  2. eight (or more) shields, arranged in two paramedian rows, enlarged relative to remaining neck shields, with no accessory shield enlarged  3. ten or more median osteoderms, combined with several lateral osteoderms, composing a distinct cervical shield |
| 474 | **Nuchal armour, morphology of nuchal shields relative to the remaining trunk dermal armour:**  *Brochu (1999, ch. 38 modified, part); Andrade et al. (2011, ch. 471).*  0. nuchal and dorsal trunk shields undifferentiated, morphology grading continuously  1. nuchal shields clearly differentiated from dorsal trunk shields by size and general  morphology (regardless of contact between nuchal and trunk series) |
| 475 | **Presacral dorsal armour, presence of accessory osteoderm columns that do not have a peg-like articulation with the paramedian column, and which are typically smaller in size than the paramedian column(s): (ORDERED)**  *Similar to the character in: Norell & Clark (1990, ch. 12 modified); Brochu (1999, ch. 37 modified); Ortega et al. (2000, ch. 107); Andrade et al. (2011, ch. 472 + 473); Young et al. (2016, ch. 290).*  0. absent (either has: two paravertebral medial columns, the gobiosuchid or notosuchian or dyrosaurid morphology)  1. present, a lateral accessory column on either of the paramedian columns  2. present, two lateral accessory columns on either of the paramedian columns |
| 476 | **Presacral dorsal armour, presence of accessory osteoderm column that has a peg-like articulation with the paramedian column (through a ‘lateral process’ derived from the anterolateral margin of the paramedian osteoderms):**  *Jouve et al. (2008, ch. 37 modified); Hastings et al. (2010, ch. 82 modified); Young et al. (2016, ch. 291).*  0. absent (either has: two paravertebral medial columns, the gobiosuchid or notosuchian or the advanced neosuchian morphology)  1. present, a lateral accessory column on either side of the paramedian columns, with articulations |
| 477 | **Presacral dorsal armour, presence of accessory osteoderm columns, anteriorly two lateral accessory columns which increase to four accessory columns in the trunk region: (NEW)**  *Ristevski et al. (2018, ch 477)*  0. absent (either has: two paravertebral medial columns, only two accessory columns, or the notosuchian morphology)  1. present |
| 478 | **Presacral dorsal armour, biserial or tetraserial dorsal shield:**  *Young et al. (2016, ch. 289).*  0. Biserial dorsal shield (one pair of paramedian osteoderm per row)  1. Tetraserial dorsal shield (two pairs of paramedian osterderms per row) |
| 479 | **Presacral dorsal armour, type of contact between elements in a row:**  *Clark (1994, ch. 98); Andrade et al. (2011, ch. 474).*  0. imbricated, any given anterior trunk osteoderm partially overlays its following element  1. sutured, osteoderms do not cover adjacent dermal elements, and are sutured if in contact |
| 480 | **Presacral dorsal armour, dimensions of the thoracic (paramedian) osteoderms:**  *Nesbitt (2011, ch. 407); Young et al. (2016, ch. 292).*  0. square shaped, length and width approximately equal  1. longer than wide  2. wider than long |
| 481 | **Presacral dorsal armour, transverse elongation of the thoracic (paramedian) osteoderms:**  *Ristevski et al. (2018, ch 481)*  0. transverse width of these osteoderms is either small or sub-equal to the anteroposterior length, or only slightly wider  1. considerably wider than long, such that the transverse width is approximately three times the anteroposterior length |
| 482 | **Presacral dorsal armour, surface of only the paravertebral osteoderms:**  *Andrade et al. (2011, ch. 476); Nesbitt (2011, ch. 404); Young et al. (2016, ch. 287).*  0. either weakly arched or mostly straight, forming a plain flat osteoderm, either keeled or not  1. osteoderm strongly curved, with convex surface, partially embracing the vertebrae from side to side |
| 483 | **Presacral dorsal armour, presence of an anterior process (= anterolateral process, stylofoveal process) to articulate with the anterior adjacent osteoderm, in medial dorsal elements:**  *Norell & Clark (1990, ch. 13 revised); Clark (1994, ch. 96); Brochu (1999, ch. 40 revised); Ortega et al. (2000, ch. 113 revised); Andrade et al. (2011, ch. 477); Young et al. (2012, ch. 233); Young et al. (2016, ch. 286).*  0. absent  1. present |
| 484 | **Presacral dorsal armour, presence of an anteroposteriorly directed keel on the dorsal surface of paramedial elements:**  *Buscalioni et al. (1992, ch. 22); Clark (1994, ch. 101 revised, part); Brochu (1999, ch. 35); Andrade et al. (2011, ch. 478); Young et al. (2016, ch. 298).*  0. absent on most/all paravertebral osteoderms  1. present along the entire (or almost all) the paravertebral osteoderms |
| 485 | **Presacral ventral osteoderms, form a carapace in the trunk region:**  *Nesbitt (2011, ch. 409 re-phrased); Young et al. (2016, ch. 294).*  0. absent  1. present |
| 486 | **Presacral ventral armour, presence of ventral collar scales:**  *Poe (1997); Brochu (1999, ch. 156); Andrade et al. (2011, ch. 479).*  0. absent, no shield enlarged relative to other ventral scales  1. present, forming a single row of enlarged scales  2. present, forming two parallel rows of enlarged scales |
| 487 | **Postsacral armour, distribution of ventral tail osteoderms:**  *Young et al. (2016, ch. 296).*  0. present  1. absent |
| 488 | **Postsacral armour, distribution when present:**  *Clark (1994, ch. 99 modified); Andrade et al. (2011, ch. 481).*  0. a pair of rows, covering the vertebral column  1. several rows, enclosing the tail surface |
| 489 | **Postsacral armour, presence of an anteroposteriorly directed keel on the dorsal surface of paramedial elements:**  *Clark (1994, ch. 101 revised part); Andrade et al. (2011, ch. 482).*  0. absent  1. present |
| 490 | **Appendicular armour, presence of osteoderms on the limbs (at least in part):**  *Nesbitt (2011, ch. 405); Young et al. (2016, ch. 288).*  0. absent  1. present |

**Soft tissue and physiology** (Ch. 493 – 496; 0.81% of characters)

| # | Description |
| --- | --- |
| 491 | **Tongue, presence of keratinised surface:**  *Brochu (1999, ch. 159); Andrade et al. (2011, ch. 483).*  0. absent  1. presence |
| 492 | **Functional lingual salt glands, presence:**  *Based on: Taplin (1985); Taplin & Grigg. (1989); Brochu (2007); Andrade et al. (2011, ch. 484).*  0. absent  1. present |
| 493 | **Internal enlarged cephalic (salt excretory) glands, presence:**  *Andrade et al. (2011, ch. 485).*  0. absent  1. present |
| 494 | ***M. caudofemoralis*, morphology:**  *Frey et al. (1989); Brochu (1999, ch. 160); Andrade et al. (2011, ch. 486).*  0. with single head  1. with double head (*longus* and *brevis*) |

**REFERENCES**

Allen ER. 2012. Analysis of North American goniopholidid crocodyliforms in a phylogenetic context. Master's thesis, University of Iowa.

Brinkmann W. 1989. Vorlaufige Mitteilung uber die Krokodilier-Faunen aus dem Ober-Jura (Kimmeridgium) der Kohlegrube Guimarota, bei Leiria (Portugal) und der Unter-Kreide (Barremium) von Uña (Provinz Cuenca, Spanien). *Documenta Naturae* **56**:1–28.Brinkmann W. 1992. Die Krokodilier-Fauna aus der Unter-Kreide (Ober-Barremium) von Uña (Provinz Cuenca, Spanien). *Berliner Geowissenschaftliche Abhandlungen* Reihe E **5**:1–123.

Buffetaut E, Ingavat R. 1980. A new crocodilian from the Jurassic of Thailand, *Sunosuchus thailandicus*n. sp. (Mesosuchia, Goniopholididae), and the palaeogeographical history of South-East Asia. *The Mesozoic Geobios* **13**: 879–889.

Buffetaut E. 1986. Une machoire de *Goniopholis* (Crocodylia, Mesosuchia) dans le Portlandien superieur du Boulonnais. *Mémoires de la Societé Académique de Boulogne-sur-mer* **1**:64–71.

Buscalioni AD, Frenegal MA, Bravo A, Poyato-Ariza FJ, Sanchiz B, Baez AM, Cambra Moo O, Martin Closas C, Evans SE, Marugan-Lobon J. 2008. The vertebrate assemblage of Buenache de la Sierra (Upper Barremian of Serrania de Cuenca, Spain) with insights into its taphonomy and palaeoecology. *Cretaceous Research* **29**:687–710.

Buscalioni AD, Martínez LA, Espílez E, Mampel L. 2013. European Goniopholididae from the Early Albian Escucha Formation in Ariño (Teruel, Aragón, Spain). *Revista española de paleontología* **28**(1):103-122.

Buscalioni AD, Sanz JL. 1987. Cocodrilos del Cretácico inferior de Galve (Teruel, España). *Estudios Geológicos* **43**(Extra): 23-43.

Erikson BR. 2016. A new skeleton of the neosuchian crocodyliform Goniopholis with new material from the Morrison Formation of Wyoming. *Science Museum of Minnesota, Monograph in paleontology* **10**: 1-29.

Halliday TJ, De Andrade MB, Benton MJ, Efimov MB. 2015. A re-evaluation of goniopholidid crocodylomorph material from Central Asia: Biogeographic and phylogenetic implications. *Acta Palaeontologica Polonica* **60**(2):291-312.

Karl HV, Groning E, Brauckmann C, Schwarz D, Knotschke N. 2006. The Late Jurassic crocodiles of the Langenberg near Oker, Lower Saxony (Germany), and description of related materials (with remarks on the history of quarrying the ‘‘Langenberg Limestone’’ and ‘‘Obernkirchen Sandstone’’). *Clausthaler Geowissenschaften* **5**:59–77.

Kuzmin IT, Skutschas PP, Grigorieva OI, Krasnolutskii SA. 2013. Goniopholidid crocodylomorph from the middle Jurassic Berezovsk quarry locality (western Siberia, Russia). *Proceedings of the Zoological Institute RAS* **317**(4):452–458.

Malafaia E, Dantas P, Ortega F, Escaso F, Gasulla JM, Ribeiro B, Barriga F, Gromicho I, García-Oliva M, Ramalheiro G, Santamaria J, Pimentel NL, Moniz C, Carvalho AGM. 2006. Análisis preliminar de la diversidad faunística en el yacimiento de Andrés (Jurásico Superior. Pombal, Portugal). *IV Encuentro de Jóvenes Investigadores en Paleontología, Salamanca. Libro de Resúmenes Salamanca*. 91–92.

Martin JE, Delfino M, Smith T. 2016. Osteology and affinities of Dollo’s goniopholidid (Mesoeucrocodylia) from the Early Cretaceous of Bernissart, Belgium. *Journal of Vertebrate Paleontology* **36**:6. DOI: 10.1080/02724634.2016.1222534.

Mazin JM, Billon-Bruyat JP, Pouech J, Hantzpergue P. 2006. The Purbeckian site of Cherves-de-Cognac (Berriasian, Early Cretaceous, southwest France): a continental ecosystem accumulated in an evaporitic littoral depositional environment. *In 9th International Symposium on Mesozoic Terrestrial Ecosystems and Biota, Abstracts and Proceedings Volume, Natural History Museum, London*. 84-88.

Mazin JM, Pouech J, Hantzpergue P, Lenglet T. 2008. The Purbeckian site of Cherves-de-Cognac (Berriasian, Early Cretaceous, SW France): a first synthesys. In: Mazin J., Pouech ., Hantzpergue ., Lacombe P. (Eds.). *Mid-Mesozoic Life and Environments*. Cognac (France*)*. 68–71.

Mazin JM, Pouech J. 2008. Crocodylomorph microremains from Champblanc (Berriasian, Cherves-de-Cognac, Charente, France). In: Mazin, J. M., Pouech, J., Hantzpergue, P., Lacombe, P. (Eds.), *Mid-Mesozoic Life and Environments*. Cognac (France). 65–67.

Mook CC. 1942. Skull characters of *Amphicotylus lucasii* Cope. *American Museum Novitates* **1165**: 1–5.

Ortega F, Moratalla JJ, Buscalioni AD, Sanz JL, Jiménez S, Valbuena J. 1996. Sobre la presencia de un cocodrilo fósil (Crocodylomorpha: Neosuchia: *Goniopholis sp.*) en la Cuenca de Cameros (Cretácico inferior: Vadillos-San Román de Cameros, La Rioja). *Zubia* **14**:113-120.

Pouech J, Mazin JM, Billon-Bruyat JP. 2006. Microvertebrate biodiversity from Cherves-de-Cognac (Lower Cretaceous, Berriasian: Charente, France). *Mesozoic Terrestrial Ecosystems*.96–100.

Pritchard AC, Turner AH, Allen ER, Norell M. 2013. Osteology of a North American goniopholidid (Eutretauranosuchus delfsi) and palate evolution in Neosuchia. *American Museum Novitates* **3783**: 1–56.

Ristevski J, Young MT, Andrade MB, Hastings AK. 2018. [A new species of *Anteophthalmosuchus* (Crocdoylomorpha, Goniopholididae) from the Lower Cretaceous of the Isle of Wight, United Kingdom, and a review of the genus](https://doi.org/10.1016%2Fj.cretres.2017.11.008). *Cretaceous Research* **84**:340–383

Ruiz-Omeñaca JI, Canudo JL. 2001. Dos yacimientos excepcionales con vertebrados continentales del Barremiense (Cretacico Inferior) de Teruel: Vallipon y La Cantalera. *Naturaleza Aragonesa* **7**:8–18.

Salisbury SW, Naish D. 2011. Crocodilians. In: Batten DJ (ed.).*English Wealden Fossils* . The palaeontological Association, London.305-369.

Salisbury SW, Willis PMA, Peitz S, Sander PM. 1999. The crocodilian *Goniopholis simus* from the Lower Cretaceous of North-western Germany. *Special Papers in Palaeontology* **60**:121–148.

Sanchez-Hernandez B, Benton MJ, Naish D. 2007. Dinosaurs and other fossil vertebrates from the Late Jurassic and Early Cretaceous of the Galve area, NE Spain. *Palaeogeography, Palaeoclimatology, Palaeoecology* **249**:180–215.

Sauvage HE. 1874. Memoire sur les dinosauriens et les crocodiliens des terrains jurassiques de Boulogne-sur-Mer. *Memoires de la Societe Geologique de France* **10**:1–58.

Sauvage HE. 1882. Synopsis des poissons et des reptiles des terrains jurassiques de Boulogne-sur-Mer. *Bulletin de la Societe geologique de France* **3**:524–547.

Schellhorn R, Schwarz-Wings D, Maisch MW, Wings O. 2009. Late Jurassic *Sunosuchus* (Crocodylomorpha, Neosuchia) from the Qigu Formation in the Junggar Basin (Xinjiang, China). *Fossil Record* **12**:59–69.

Schwarz D. 2002. A new species of *Goniopholis* from the Upper Jurassic of Portugal. *Palaeontology* **45**:185–208.

Schwarz-Wings D, Rees J, Lindgren J. 2009. Lower cretaceous mesoeucrocodylians from Scandinavia (Denmark and Sweden). *Cretaceous Research* **30**(5):1345–1355.

Smith DK, Allen ER, Sanders RK, Stadtman KL. 2010. Anew specimen of *Eutretauranosuchus* (Crocodyliformes; Goniopholididae) from Dry Mesa, Colorado. *Journal of Vertebrate Paleontology* **30**:1466–1477. DOI:10.1080/02724634.2010.501434

Tykoski RS, Rowe TB, Ketcham RA, Colbert, MW. 2002. *Calsoyasuchus valliceps*, a new crocodyliform from the Early Jurassic of Kayenta Formation of Arizona. *Journal of vertebrate Paleontology* **22**: 593–611.

Vullo R, Abit D, Ballère M, Billon-Bruyat J-P, Bourgeais R, Buffetaut E, Daviero-Gomez V, Garcia G, Gomez B, Mazin J-M, Morel S, Neraudeau D, Pouech J, Rage J-C, Schnyder J, Tong H. 2014. Palaeontology of the Purbeck-type (Tithonian, Late Jurassic) bonebeds of Chassiron (Oléron Island, western France). Comptes Rendus Palevol **12**:421–441.
